# The Mouse Gut Microbial Biobank expands the coverage of cultured bacteria

**DOI:** 10.1038/s41467-019-13836-5

**Published:** 2020-01-07

**Authors:** Chang Liu, Nan Zhou, Meng-Xuan Du, Yu-Tong Sun, Kai Wang, Yu-Jing Wang, Dan-Hua Li, Hai-Ying Yu, Yuqin Song, Bing-Bing Bai, Yuhua Xin, Linhuan Wu, Cheng-Ying Jiang, Jie Feng, Hua Xiang, Yuguang Zhou, Juncai Ma, Jun Wang, Hong-Wei Liu, Shuang-Jiang Liu

**Affiliations:** 1 State Key Laboratory of Microbial Resources, Institute of Microbiology, Chinese Academy of Sciences, No. 1 Beichenxi Road, Chaoyang District, Beijing, 100101 P. R. China; 2 Environmental Microbiology Research Center, Institute of Microbiology, Chinese Academy of Sciences, No. 1 Beichenxi Road, Chaoyang District, Beijing, 100101 P. R. China; 3 University of Chinese Academy of Sciences, Beijing, 100049 P. R. China; 4 State Key Laboratory of Mycology, Institute of Microbiology, Chinese Academy of Sciences, No. 1 Beichenxi Road, Chaoyang District, Beijing, 100101 P. R. China; 5 CAS Key Laboratory of Pathogenic Microbiology and Immunology, Institute of Microbiology, Chinese Academy of Sciences, No. 1 Beichenxi Road, Chaoyang District, Beijing, 100101 P. R. China; 6 Microbial Resources and Big Data Center, Institute of Microbiology, Chinese Academy of Sciences, No. 1 Beichenxi Road, Chaoyang District, Beijing, 100101 P. R. China

**Keywords:** Microbiome, Microbiome, Symbiosis, Symbiosis

## Abstract

Mice are widely used as experimental models for gut microbiome (GM) studies, yet the majority of mouse GM members remain uncharacterized. Here, we report the construction of a mouse gut microbial biobank (mGMB) that contains 126 species, represented by 244 strains that have been deposited in the China General Microorganism Culture Collection. We sequence and phenotypically characterize 77 potential new species and propose their nomenclatures. The mGMB includes 22 and 17 species that are significantly enriched in ob/ob and wild-type C57BL/6J mouse cecal samples, respectively. The genomes of the 126 species in the mGMB cover 52% of the metagenomic nonredundant gene catalog (sequence identity ≥ 60%) and represent 93–95% of the KEGG-Orthology-annotated functions of the sampled mouse GMs. The microbial and genome data assembled in the mGMB enlarges the taxonomic characterization of mouse GMs and represents a useful resource for studies of host-microbe interactions and of GM functions associated with host health and diseases.

## Abstract (web summary)

Here, the authors established and characterized the mouse gut microbial biobank (mGMB), which includes 244 strains and 126 species that enlarges previous mouse intestinal bacterial collections and represents a resource for studies using mouse models to investigate microbiome-associated health and disease.

## Introduction

The gut microbiota (GM), an emerging organ and the most complex ecosystem in hosts, is essential to human health, and can result in diseases when it becomes dysbiosis^1,2^. Health care and life management require an understanding of the microbiome associated with the human body. Due to ethical and safety considerations, many experimental studies of human–GM interactions must be carried out with animal models. As a frequently used model system, experimental mice have become a mainstay in GM studies^3^. Scientists have developed germ-free^4^, ASF (altered Schaedler flora)^5^, HFA (human flora-associated)^6^, genetically modified, and inducible disease mouse models^7–11^. One example is the *ob/ob* mouse (also known as the Lep^*ob/ob*^ and leptin-deficient mouse), a model for studying the interplay between GM and metabolic diseases such as metabolic syndrome, obesity, and diabetes^9^. Thus far, the understanding of mouse GM is very limited. According to our own analysis of published 16S rRNA gene amplicon datasets of mouse GMs^12–16^, ~90% of the operational taxon units (OTUs) of the mouse GMs could not be precisely assigned at the species level with the All-Species Living Tree database (version 132)^17^, as their corresponding taxa have not been cultured and identified. This seriously impedes the understanding and interpretation of the massive amount of metagenomic data of mouse GMs. In recent years, cultivation-dependent studies of human GMs have enabled the identification of hundreds of previously unknown bacteria inhabiting the human intestines^18–22^. However, these large-scale gut microbe cultivations and characterizations mainly focused on humans but scarcely on animal models. Several studies have demonstrated that human-originated microbes have problems colonizing and functioning in mouse guts^23–26^. Thus, the collection of cultured gut microbes from mouse models is imperative. A recent milestone work on mouse intestinal bacterial collection (miBC) was carried out by collecting gut microbes from diverse mice^12^. The miBC harbored 76 species, and recovered less than 10% of the mouse GM at the species level, leaving a gigantic space for the cultivation and further investigation of gut microbes. Consequently, researchers have frequently met serious difficulties when culture-dependent experiments are needed, such as causative studies or strain-specific interventions. To challenge these difficulties, extensive cultivation and characterization of gut microbes from mouse models are urgently needed.

## Results

### Bacterial isolation reveals previously uncultured taxa

The large-scale cultivation and identification of mouse gut microbes was performed following the simple workflow (Supplementary Fig. 1, steps 1–5.3), and the outcomes of each step are shown in the red dashed box in Supplementary Fig. 1. In brief, after the first three working steps, we obtained 1831 isolates that were grouped into 154 bacterial taxa based on the 16S rRNA gene identity and by applying a cutoff value of 98% for different taxa. As shown in Supplementary Fig. 2, only 51 out of 154 taxa were assigned to a previously described species (white background). The other 103 taxa could not be assigned to any known species (light-blue background), suggesting that they represent potential novel taxa. The 1831 isolates were then inoculated for large-scale cultivation, but 394 of them did not propagate during further cultivation (Supplementary Fig. 1, step 4). The remaining 1437 isolates belonged to 126 different taxa. The identity and 16S rRNA gene sequence of each isolate are documented in Supplementary Data 1. After strain cryopreservation (Supplementary Fig. 1, step 5.1), 244 strains representing the 126 cultured taxa were obtained and are available for public use. The draft genomes of 126 cultured taxa were then sequenced and made publicly accessible via NCBI, gcMeta, and NODE (Supplementary Fig. 1, step 5.2). The functional diversity of all 126 cultured taxa is displayed in the KO-based functional distances shown in Supplementary Fig. 3. Of the 126 taxa, 77 were potential novel taxa, according to the 16S rRNA sequence identity of the known species included in the NCBI 16S ribosomal RNA sequence database (Update date: 2019/07/08, number of sequences: 20,767). Two papers on cultured human gut microbial genomes were recently published^20,21^ while this paper was being reviewed, and we further validated the novelty of the 77 taxa by comparing their 16S rRNA sequences with the previously published human gut microbial genome collections of four different studies^18,20–22^. The results showed that three taxa of the 77 taxa (Taxon 55, Taxon 72, and Taxon 149) identified >98% of 16S rRNA gene sequence of the isolates from the two most recently published studies^20,21^, suggesting that they were previously cultured and sequenced. However, only genomes of these three taxa were reported, but not their morphology, physiological, and biochemical properties. In this work, the 77 taxa were polyphasically characterized by (1) phylogenetic analysis, (2) morphology observation, (3) phenotypic characterization via BIOLOG tests, and (4) genome analysis/comparison (Supplementary Fig. 1, step 5.3). As a result, 77 novel species were identified, and 43 new genera were recognized. Detailed descriptions of the 77 taxa and their proposed nomenclatures are provided in Supplementary Data 2 and Supplementary Figs. 5–81, which also include their phylogeny, morphology, and other phenotypical features. With these efforts, we constructed the largest-to-date mGMB comprising 244 strains representing 126 species from 80 genera, 28 families and 5 phyla, which have been deposited in China General Microbiological Culture Collection Center (CGMCC) for public use, and 126 draft genomes that are publicly accessible as well (Supplementary Data 3).

### The mGMB expands the diversity of the existing collection

**Fig. 1:**
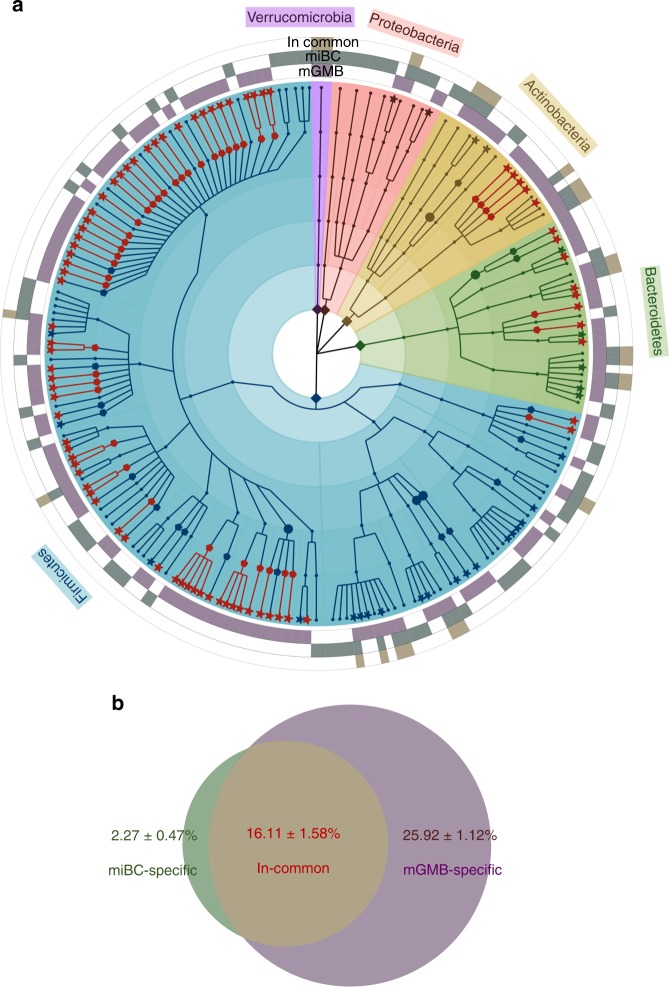
The taxonomic diversity and contribution of mGMB to the mouse intestinal bacterial collection. **a** The cladogram displays the taxonomic diversity of bacteria in the mGMB and miBC. The mGMB and miBC together have 180 bacterial species from 110 genera, 33 families, and 5 phyla. The background is color-coded according to phyla. All 104 species unique to the mGMB are marked with star symbols, and the 77 new species are indicated with red stars. All 62 genera uniquely represented by mGMB members are indicated with pentagon symbols, and the 43 newly identified genera are indicated with red pentagons. The five families unique to the mGMB are symbolized by solid circles. The 126 species in the mGMB are indicated by the first-level purple external ring (labeled as mGMB in panel **a**). The 76 species in the miBC are indicated by the second-level green external ring (labeled as miBC in panel **a**). The 22 species in both the mGMB and miBC are indicated by the third-level yellow external ring (labeled as in common in panel **a**). **b** The Venn diagram displays the read coverage of mGMB and miBC to the 16S rRNA gene amplicon dataset of mouse samples (*n* = 93) from miBC work^12^ at species level (sequence identity > 97%). The miBC-specific (2.27 ± 0.47%): the proportion of reads uniquely covered by the miBC; The mGMB-specific (25.92 ± 1.12%): the proportion of reads uniquely covered by the mGMB; The In-common (16.11 ± 1.58%): the proportion of reads shared by both collections. The coverage rate is present as mean ± SEM. Source data are provided as a Source Data file.

The first mouse intestinal bacterial collection (miBC) contained 76 cultured bacterial species^12^. As shown in the taxonomic cladogram (Fig. 1a), the construction of the mGMB greatly expanded the cultured mouse gut microbial repository by increasing the number of species from 76 to 180 and the number of genera from 48 to 110. The mGMB and miBC overlapped by 22 bacterial species. The constructed mGMB alone contributed 104 unique species, including 77 novel species and 43 novel genera. As shown in Fig. 1b, the 76 species of miBC alone covered 18.37 ± 1.55% (mean ± SEM) of the total reads of 16S rRNA gene amplicons of 93 samples used in miBC work^12^ at the species level (16S rRNA gene identity >97%), while the 126 species of mGMB covered 42.20 ± 1.29% of the total reads. There were 16.11 ± 1.58% reads shared by both collections. The two collections jointly covered 44.29 ± 1.40% of the total reads at the species level with 2.27 ± 0.47% and 25.92 ± 1.12% of the reads exclusively contributed by miBC and mGMB, respectively.

### The 77 new species in the mGMB are prevalent in mouse guts

**Fig. 2:**
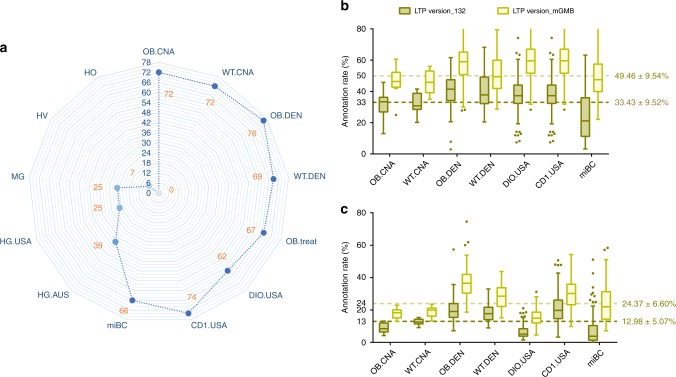
The 16S rRNA gene amplicon-based analysis of the prevalence of novel species in the mGMB. **a** Radar plot depicting the prevalence of 77 novel taxa within the host-associated microbiotas from diverse hosts. The numbers of novel species that got hits on OTUs in datasets were marked in orange. OB.CNA: the gut microbiota of *ob/ob* mice from China (*n* = 12), WT.CNA: the gut microbiota of C57BL/6J mice from China (*n* = 12), OB.DEN: the gut microbiota of *ob/ob* mice from Denmark (*n* = 239), WT.DEN: the gut microbiota of C57BL/6J mice from Denmark (*n* = 120), OB.tre = t: the gut microbiota of *ob/ob* mice from China treated with anti-metabolic-syndrome medicine SA-7 (*n* = 31), DIO.USA: the gut microbiota of diet-induced obese mice from the USA (*n* = 25), CD1.USA: the gut microbiota of outbred CD1 mice from the USA (*n* = 208), miBC: the gut microbiota of mice with different genetic backgrounds and housed in various facilities in Europe and America from miBC (*n* = 93), HG.AUS: the gut microbiota of humans from Australia (*n* = 300), HG.USA: the gut microbiota of humans from USA (*n* = 97), MG: the gut microbiota of rhesus monkeys from China (*n* = 160), HV: the microbiota of the human vagina (*n* = 20), HO: the microbiota of the human oral cavity (*n* = 66). **b**, **c** The novel taxa improved the annotation rate of the 16S rRNA gene amplicon data of murine GMs at the genus level (**b**) and at the species level (**c**). LTP version_132 (olive drab): data annotated using LTP database version 132, LTP version_mGMB (light yellow): data annotated using a customized LTP database by supplementing the 16S rRNA gene sequences of 77 novel species; data are shown in box-and-whiskers plot, center line: median, bounds of box: quartile, whiskers: Tukey extreme; the mean ± SEM of annotation rates using different databases were given in the panels and were statistically determined to be significantly different (*p* < 0.001) by *t* test. The *n* numbers represent the biologically independent samples. Source data are provided as a Source Data file.

The 77 new bacterial species in the mGMB originated from a specific mouse genotype (*ob/ob* C57BL/6J) purchased from one local vendor. To evaluate how widely these new taxa occur in mouse guts, we exploited publicly available 16S rRNA gene amplicon datasets of mouse GMs from this and previous studies (Supplementary Data 4). In total, 16S rRNA gene amplicon data of 740 mouse samples from six studies were collected from the NCBI SRA database and mined for the occurrence of these new taxa. As shown in Fig. 2a, the new taxa were identified in samples from wild-type C57BL/6J (69–72 out of 77 taxa), CD1 (74 out of 77 taxa), and various-background mice from the miBC study (66 out of 77 taxa), in addition to *ob/ob* mice (72–76 out of 77 taxa). Figure 2a and Supplementary Data 4 show that those new bacterial taxa were also prevalent in mice from various vendors and in obese mice induced by high-fat diets or treated with medical agents. Considering the high prevalence of these new taxa in mouse GMs, we upgraded the LTP database (version 132) with the 16S rRNA gene sequences of the type strains of the 77 new taxa (named LTP version_mGMB) and reannotated the amplicon datasets listed in Supplementary Data 4 with the upgraded database, LTP version_mGMB. As a result, the annotation rate of the sequence datasets dramatically increased to 49.46 ± 9.54% from 33.43 ± 9.52% of the total reads at the genus level (sequence identity > 95%) and to 24.37 ± 6.60% from 12.98 ± 5.07% at the species level (sequence identity > 97%) (Fig. 2b, c). Interestingly, we observed that 25 and 39 out of the 77 novel taxa were identified from human GMs in American and Australian studies (Fig. 2a), respectively. We also found that none of the 77 novel taxa were identified from human oral and vaginal microbiota, suggesting that those novel taxa might be gut-adapted bacterial populations.

### The mGMB largely defines the gut core- and pan-microbiota

**Fig. 3:**
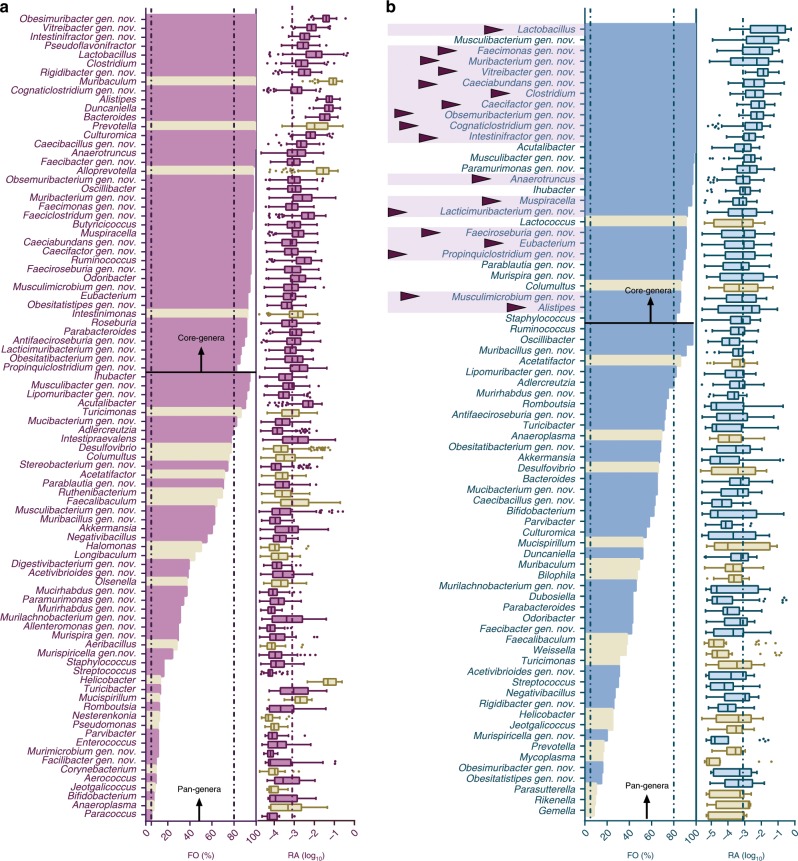
The mGMB defines the core- and pan-microbiota of mouse gut microbiota. **a** The mGMB coverage of the core- and pan-genera of the *ob/ob* mice. **b**, The mGMB coverage of the core- and pan-genera of the diverse-background mice. The bar chart shows the frequency of occurrence (FO) of each genus in the analyzed samples (definition: FO = 100% is defined when a genus is present in all samples, while FO = 0 is defined when a genus is present in none of the samples), *n* = 274 in (**a**), *n* = 93 in (**b**); the box-and-whiskers plot shows the relative abundance (RA) of each taxon, center line: median, bounds of box: quartile, whiskers: Tukey extreme. The RA is exhibited in the percentage value logarithm. Core-genera: genera with FO > 80% and an average RA > 0.1% (log _10_ (RA) > −3); pan-genera: genera with FO > 5%. The cutoff values for core- and pan-genera are marked with vertical dashed lines in the panel; purple/blue: genera covered by the mGMB; cream: genera not covered by the mGMB; purple triangle markers: core-genera for both the *ob/ob* and diverse-background mice^12^. The *n* numbers represent the biologically independent samples. Source data are provided as a Source Data file.

In addition to the 77 new bacterial species, an additional 49 previously described bacterial species were also cultured in this study and were collected in the mGMB. With those total of 126 species in the mGMB, we attempted to identify the mGMB coverage of the potential gut core- and pan-microbiota of mice. For this purpose, we collected the available 16S rRNA gene amplicon raw data of *ob/ob* mouse GMs (*n* = 274) from the NCBI SRA database and performed an integrated analysis of these data, as described in the “Methods” section. If we define the core-genera as those with an FO (frequency of occurrence) > 80% and an RA (relative abundance) > 0.1% and the pan-genera as those with an FO > 5%, 36 and 80 core- and pan-genera were recognized in the GMs of *ob/ob* mice, from a total of 129 annotated genera in the 274 analyzed samples. The pan-genera covered 99.8 ± 0.2% and the core-genera covered 92.3 ± 0.6% of all the annotated reads on average. As shown in Fig. 3a, the mGMB recovered 35 out of 40 core-genera and 68 out of the 90 pan-genera for the *ob/ob* mice. The mGMB coverage rate of the core- and pan-genera reached 88% and 75%, respectively.

Second, to determine whether the mGMB is generally representative of the GMs of diverse-background mice, we explored the core- and pan-genera of diverse-background mice reported by miBC^12^. By applying the above definition of core- and pan-genera, 74 genera were recognized as pan-genera and 28 as core-genera (Fig. 3b) for the diverse-background mice described in the miBC. As shown in Fig. 3b, the mGMB recovered 26 out of the 28 core-genera and 56 out of the 74 pan-genera. The coverage rates reached 93% and 76%, respectively. Moreover, of the 28 core-genera, 18 were also core in *ob/ob* mice, and all 18 of the shared core-genera were covered by the mGMB. The above results clearly indicated that the mGMB had good coverage of both the *ob/ob* and the diverse-background mice.

### The mGMB covers the major functionality of mouse GMs

**Fig. 4:**
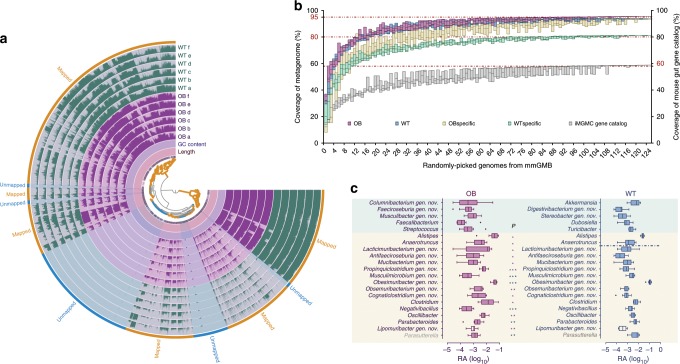
The metagenomic functions covered by the mGMB and the GM features associated with mouse phenotypes. **a** The mapping profile of metagenomic short reads of OB and WT to the mGMB pan-genome. OB: metagenomes of *ob/ob* mouse GMs (*n* = 6); WT: metagenomes of C57BL/6J mouse GMs (*n* = 6). The Anvi’o tree displays the hierarchical clustering of the mGMB pan-genome; WT layers a–f represent the detection of each split in WT; OB **a**–**f** represents the detection of each split in OB; the outermost yellow layer displays the splits in the pan-genome mapped by the metagenomic reads; the most outer blue layer displays the splits in the pan-genome unmapped to the metagenomic reads. **b** KO-based coverage of mouse gut microbiomes by genomes randomly picked from the mGMB. OB: the KEGG Ortholog (KO) pool of metagenomes of ob/ob mouse GMs (*n* = 6); WT: the KO pool of metagenomes of C57BL/6J mouse GMs (*n* = 6); OB-specific: KOs appearing specifically in OB; WT-specific: KOs appearing specifically in WT; iMGMC gene catalog: the integrated mouse gut metagenome catalog (iMGMC) comprising 4.6 million unique genes and 660 high-quality metagenome-assembled genomes^27^. Data are shown with box plot, center line: median, bounds of box: extreme. **c** The features of the gut microbiota at the genus level associated wild-type (C57BL/6J) and metabolic-syndrome (*ob/ob* C57BL/6J) mice. OB: the 16S rRNA gene amplicons of *ob/ob* mouse cecal samples (*n* = 12) used for previous bacterial isolation; WT: the 16S rRNA gene amplicons of the counterpart wild-type C57BL/6J mice (*n* = 12). Data are shown in a box-and-whiskers plot, center line: median, bounds of box: quartile, whiskers: Tukey extreme; statistical comparisons of the data between the two groups used the *t* test, ^∗^*P* < 0.05; ^∗∗^*P* < 0.01; ^∗∗∗^*P* < 0.001; purple: enriched in OB, blue: enriched in WT; mint-colored background: genera exclusively presenting in either the OB or WT groups; cream-colored background: genera presenting in both groups but with significant difference in RA; gray genus names: genera that were not recovered by the mGMB. The n numbers represent the biologically independent samples. *P*-values are provided as a Source Data file.

To assess the functional coverage of the mGMB for the mouse gut microbiome, we assembled the 126 draft genomes in the mGMB and sequenced the metagenomes of cecal samples from both the *ob/ob* (OB, *n* = 6) and wild-type mice (WT, *n* = 6). The mGMB pan-genome was generated by merging the 126 draft genomes from the mGMB. In total, 54.5 and 50.6 gB of metagenomic clean data were obtained for the OB and WT mouse cecal samples, respectively. Then, quality-filtered metagenomic reads of both the OB and WT samples were mapped to the mGMB pan-genome and the mapping profile was visualized with Anvi’o (Fig. 4a). The total length of the mGMB pan-genome was 548 Mb, and more than 80.1% of the DNA sequences of the mGMB pan-genome were mapped by the metagenomic reads from either WT or OB (the outermost yellow layer in Fig. 4a). An analysis of the mapping results with SAMtools revealed that on average, 24.9 ± 3.3% and 24.0 ± 1.9% of the metagenomic reads from OB and WT samples, respectively, were mapped to the mGMB pan-genome. The metagenomic reads of the OB and WT samples (*n* = 12) were further assembled for the prediction of open-reading frames and subsequent extraction of nonredundant unique gene catalogs as described in Methods. To demonstrate the coverage of the 126 draft genomes to the metagenomes at the gene level, we performed a BLASTp analysis of the nonredundant metagenomic genes against the mGMB pan-genome. The results revealed that 52% and 72% of the unique genes in the catalog were covered by the mGMB genomes when the cutoff values for amino acid sequence identity were set at 60% and 40%, respectively.

To further investigate the mGMB representativeness of known GM functions, the nonredundant metagenomic genes of the OB and WT samples (*n* = 12) and the mGMB genomes were annotated with the KO (KEGG Orthology) database. On average, 34.64 ± 0.34% of the metagenomic genes were annotated into known KOs. A profile of the presence/absence binary code (0/1) of KOs for each draft genome as well as the OB and WT metagenomes was generated. A cumulative analysis of the KO profiles (Supplementary Data 5) was conducted by random incremental selection of the genomes from the mGMB. As shown in Fig. 4b, the coverage rates for both OB and WT reached up to 80% and approached saturation when the sampling number increased to 16, indicating that the best-fitting 16 genomes from the mGMB might well represent the functionality of the mouse GMs. Finally, the 126 draft genomes covered 93% and 95% of the KO-based functionality of WT and OB mice, respectively. In addition to the metagenomic data from this study, we also revisited the biggest integrated mouse gut metagenome catalog (iMGMC) comprising 4.6 million unique genes and 660 high-quality metagenome-assembled genomes^27^. The same cumulative analysis was applied to the iMGMC. As depicted in Fig. 4b (the gray box), the 126 mGMB draft genomes covered over 59% of the KO-based functions of the iMGMC.

### GM features are associated with mouse phenotypes

In total, 3455 and 3521 KOs (Supplementary Data 5) were annotated for the gut metagenomes of the OB (*ob/ob* C57BL/6J with metabolic-syndrome-related phenotype) and WT (C57BL/6J) mice, respectively. The statistics showed that the compositions of the two KO pools from the OB and WT mice were significantly different (*P* = 0.003), although the two groups shared 3368 KOs (Supplementary Fig. 4a). There were 153 and 86 KOs uniquely present in the WT and OB mice, respectively, and 352 and 250 out of the shared 3368 KOs were enriched in OB and WT mice, respectively (Supplementary Fig. 4b). As shown in Fig. 4b, the mGMB represented OB-specific functions (93%) better than WT-specific functions (80%). With these functional features, we further explored how bacterial members of the mGMB reflect potential gut microbiota features at taxonomic levels.

The gut microbiota features associated with either C57BL/6J or *ob/ob* C57BL/6J mice were identified by comparing the 50 most abundant genera generated by an analysis of the 16S rRNA gene amplicon dataset from *ob/ob* (OB, *n* = 12) and wild-type mouse cecal samples (WT, *n* = 12) (Fig. 4c). The mGMB covered all the candidate genera, except the genus *Parasutterella*, which was not cultivated in this study. To further characterize the potential phenotype-associated features at the microbial species level, local BLAST analysis of the 16S rRNA gene sequences of species in the mGMB compared with the OTU sequences generated from the 16S rRNA gene amplicon datasets of OB (*n* = 12) and WT (*n* = 12) was performed. As shown in Table 1, the *t* test-based statistical analysis of the RA for each OTU hit revealed that 22 and 17 mGMB species were significantly enriched in OB and WT mice, respectively. The majority of the OB-enriched species came from *Firmicutes* (mainly from the orders *Clostridiales* and *Lactobacillales*), while most of the WT-enriched species belonged to *Bacteroidetes*.

**Table 1:** Relative abundances of mGMB bacteria in *ob/ob* and C57BL/6J mice.

Taxon number	Taxonomy	RA of WT	RA of OB	Significance		Taxon number	Taxonomy	RA of WT	RA of OB	Significance	
	*Akkermansiaceae*						*Lachnospiraceae*				
Taxon 151	*Akkermansia muciniphila*	7.05E–03	2.34E–04	***	WT	Taxon 43	*Obsemuribacterium intestinalis*	7.75E–04	3.33E–03	***	OB
	*Aerococcaceae*			ns		Taxon 61	*Caecifactor intestinalis*	1.36E–03	4.06E–03	ns	
Taxon 4	*Aerococcus urinaeequi*	0.00E+00	1.40E–04	ns		Taxon 62	*Paramurimonas intestinalis*	1.00E–03	1.50E–03	ns	
	*Bacillaceae*					Taxon 73	*Lacticimuribacterium intestinale*	8.53E–03	1.06E–02	ns	
Taxon 8	*Bacillus foraminis*	2.29E–06	1.15E–05			Taxon 74	*Obesitatistipes intestinalis*	1.17E–03	2.40E–03	ns	
Taxon 111	*Bacillus aerius*	9.18E–06	9.18E–06	ns		Taxon 78	*Caeciabundans intestinalis*	5.57E–04	5.30E–04	ns	
	*Bacteroidaceae*					Taxon 81	*Musculimicrobium intestinale*	2.56E-03	6.26E–04	***	WT
Taxon 33	*Bacteroides rodentium*	1.91E–02	1.25E–02	ns		Taxon 82	*Murispira intestinalis*	2.52E–05	2.02E–04	ns	
Taxon 53	*Bacteroides acidifaciens*	3.92E–02	2.58E–02	ns		Taxon 83	*Faecimonas intestinalis*	8.49E–05	9.41E–05	ns	
Taxon 54	*Bacteroides caecimuris*	3.38E–03	3.55E–03	ns		Taxon 85	*Parablautia intestinalis*	1.08E–04	9.41E–05	ns	
Taxon 141	*Bacteroides uniformis*	1.74E–04	7.57E–05	***	WT	Taxon 86	*Parablautia muris*	1.08E–04	1.61E–04	ns	
Taxon 152	*Bacteroides vulgatus*	1.02E–03	3.88E–04	*	WT	Taxon 87	*Intestipraevalens muris*	6.31E–03	4.60E–03	ns	
Taxon 153	*Bacteroides sartorii*	1.67E–02	1.29E–02	ns		Taxon 88	*Murispiricella intestinalis*	1.22E–03	9.08E–04	ns	
	*Bifidobacteriaceae*					Taxon 89	*Muspiracella faecis*	1.82E–03	1.36E–03	ns	
Taxon 17	*Bifidobacterium pseudolongum*	7.69E–04	2.89E–04	ns		Taxon 90	*Musculibacter intestinalis*	2.66E–04	9.34E–04	ns	
Taxon 71	*Bifidobacterium longum*	7.11E–05	3.21E–05	*	WT	Taxon 93	*Faecibacter intestinalis*	1.09E–03	1.85E–03	ns	
	*Clostridiaceae*					Taxon 94	*Muribacillus muris*	5.74E–05	1.72E–04	**	OB
Taxon 10	*Clostridium cocleatum*	7.96E–04	6.42E–04	*	OB	Taxon 99	*Roseburia muris*	3.52E–03	1.16E–02	*	OB
Taxon 42	*Butyricicoccus muris*	6.77E–04	1.51E–03	ns		Taxon 101	*Paramurimonas faecis*	1.00E–03	2.66E–03	ns	
Taxon 45	*Cognaticlostridium intestinale*	8.81E–04	4.28E–03	**	OB	Taxon 114	*Lactobacillus caviae*	2.64E–03	6.09E–03	*	OB
Taxon 47	*Propinquiclosttridium intestinale*	4.82E–04	2.04E–03	**	OB	Taxon 116	*Lactobacillus vaginalis*	2.64E–03	6.09E–03	*	OB
Taxon 50	*Clostridium faecis*	9.81E–03	6.86E–03	ns		Taxon 117	*Lactobacillus reuteri*	2.64E–03	6.09E–03	*	OB
Taxon 92	*Propinquiclostridium muris*	7.02E–04	3.53E–03	**	OB	Taxon 118	*Muribacterium intestinale*	1.40E–02	1.69E–02	ns	
Taxon 96	*Propinquiclostridium caecimuris*	4.57E–04	2.95E–03	*	OB	Taxon 119	*Lactobacillus intestinalis*	1.87E–02	2.11E–02	ns	
Taxon 103	*Faeciclostridum intestinalis*	1.86E–03	3.93E–03	ns		Taxon 120	*Caecibaculum intestinale*	3.18E–03	4.70E–03	ns	
Taxon 115	*Facilibacter intestinalis*	1.45E–02	7.07E–03	ns		Taxon 131	*Columultus intestinalis*	5.05E–05	4.07E–03	ns	
	*Clostridiales IncertaeSedisXIII*						*Muribaculaceae*				
Taxon 13	*“Ihubacter muris”*	1.62E–03	2.23E–03	ns		Taxon 29	*Duncaniella intestinalis*	2.00E–02	1.63E–02	**	WT
Taxon 40	*Murimicrobium intestinale*	2.98E–05	9.64E–05	ns		Taxon 52	*Caecibacillus intestinalis*	6.88E–04	4.57E–04	ns	
Taxon 104	*Murirhabdus intestinalis*	9.06E–04	9.61E–04	ns		Taxon 64	*Duncaniella muris*	6.62E–03	5.07E–03	ns	
Taxon 105	*Digestivibacterium intestinale*	1.62E–03	2.23E–03	ns		Taxon 72	*Obesimuribacter intestinalis*	5.05E–02	2.95E–02	**	WT
	*unclassified Clostridiales*						*Odoribacteraceae*				
Taxon 41	*Intestinifractor faecalis*	1.18E–02	9.03E–03	ns		Taxon 68	*“Culturomica muri”*	4.36E–05	8.05E–04	ns	
Taxon 60	*Intestinifractor caecimuris*	1.18E–02	9.01E–03	ns			*Oscillospiraceae*				
Taxon 76	*Pseudoflavonifractor muris*	3.05E–03	1.01E–02	*	OB	Taxon 123	*Rigidibacter intestinalis*	3.26E–03	3.83E–03	ns	
Taxon 130	*Intestinifractor faecis*	1.18E–02	1.00E–02	ns		Taxon 125	*Vitreibacter intestinalis*	3.20E–03	2.48E–03	ns	
Taxon 132	*Intestinifractor muris*	4.42E–03	2.44E–03	**	WT	Taxon 126	*Vitreibacter faecis*	7.10E–03	7.51E–03	ns	
Taxon 133	*Intestinifractor intestinalis*	1.18E–02	1.00E–02	ns		Taxon 127	*Vitreibacter muris*	8.42E–04	1.03E–03	ns	
	*Coriobacteriaceae*					Taxon 129	*Vitreibacter caecimuris*	2.82E–03	4.12E–03	ns	
Taxon 51	*Parvibacter caecicola*	6.88E–05	1.70E–04	ns			*Peptostreptococcaceae*				
	*Eggerthellaceae*					Taxon 55	*Romboutsia muris*	2.28E–03	2.45E-03	ns	
Taxon 19	*Stereobacter intestinalis*	4.86E–04	7.11E–05	**	WT	Taxon 106	*Peptoclostridium difficile*	6.88E–06	1.61E-05	ns	
Taxon 22	*Adlercreutzia mucosicola*	3.23E–04	4.96E–04	ns			*Propionibacteriaceae*				
Taxon 23	*Mucirhabdus intestinalis*	3.23E–04	3.62E–04	ns		Taxon 145	*Cutibacterium acnes*	9.18E–06	1.93E–04	ns	
Taxon 25	*Stereobacterium intestinale*	6.88E–06	1.86E–04	ns			*Rikenellaceae*				
Taxon 142	*Adlercreutzia muris*	2.84E–04	2.71E–04	ns		Taxon 67	*Alistipes muris*	3.04E–03	1.39E–03	***	WT
Taxon 143	*Adlercreutzia caecimuris*	2.84E–04	4.22E–04	ns			*Ruminococcaceae*				
Taxon 144	*Adlercreutzia faecis*	2.84E–04	4.22E–04	ns		Taxon 11	*Mucibacterium intestinale*	5.02E–04	1.50E–03	**	OB
	*Enterococcaceae*					Taxon 12	*Anaerotruncus muris*	2.07E–03	4.15E–03	ns	
Taxon 5	*Enterococcus gallinarum*	3.44E–05	2.00E–04	ns		Taxon 37	*Acutalibacter faecis*	1.17E–03	3.23E–03	***	OB
Taxon 6	*Enterococcus faecalis*	3.44E–05	4.36E–05	ns		Taxon 38	*Lipomuribacter intestinalis*	4.29E–04	1.07E–03	**	OB
Taxon 108	*Enterococcus asini*	3.44E–05	3.90E–05	ns		Taxon 39	*Lipomuribacter faecis*	4.29E–04	9.27E–04	**	OB
Taxon 109	*Enterococcus xiangfangensis*	3.44E–05	3.90E–05	ns		Taxon 56	*Anaerotruncus colihominis*	5.05E–05	5.05E–05	ns	
	*Erysipelotrichaceae*					Taxon 75	*Acetivibrioides intestinalis*	5.04E–03	9.86E–03	ns	
Taxon 59	*Dubosiella newyorkensis*	9.68E–04	2.06E–05	**	WT	Taxon 95	*Ruminococcus muris*	9.81E–03	6.92E–03	ns	
Taxon 66	*Turicibacter muris*	2.37E–03	1.72E–04	***	WT	Taxon 121	*Acetivibrioides faecis*	2.17E–03	2.38E–03	ns	
Taxon 122	*Obesitatibacterium intestinale*	4.29E–04	1.03E–03	*	OB	Taxon 139	*Acutalibacter intestinalis*	1.17E–03	3.04E–03	***	OB
	*Eubacteriaceae*					Taxon 140	*“Negativibacillus muris”*	1.99E–03	5.30E–04	***	WT
Taxon 97	*Eubacterium contortum*	9.66E–04	2.55E–04	ns			*Staphylococcaceae*				
Taxon 98	*Eubacterium muris*	2.12E–03	2.66E–04	***	WT	Taxon 110	*Staphylococcus warneri*	3.44E–05	2.06E–05	ns	
	*Lactobacillaceae*						*Tannerellaceae*				
Taxon 1	*Lactobacillus murinus*	3.47E–03	1.22E–02	***	OB	Taxon 34	*Parabacteroides distasonis*	5.01E–03	1.44E–03	**	WT
Taxon 14	*Faeciroseburia intestinalis*	9.41E–05	4.43E–04	**	OB	Taxon 77	*Parabacteroides goldsteinii*	7.46E–04	7.80E–04	ns	
Taxon 15	*Antifaeciroseburia intestinalis*	3.49E–04	2.20E–03	**	OB	Taxon 149	*Parabacteroides muris*	5.01E–03	2.13E–03	*	WT
Taxon 2	*Lactobacillus taiwanensis*	1.73E–02	2.23E–02	ns		Taxon 154	*Parabacteroides intestinalis*	5.01E–03	1.37E–03	**	WT
Taxon 3	*Lactobacillus johnsonii*	1.74E–02	3.64E–02	*	OB		*Streptococcaceae*				
						Taxon 107	*Streptococcus acidominimus*	0.00E+00	1.84E–05	ns	

The statistical analysis of data between OB and WT groups used the *t* test, and the test significance is marked with asterisks: ^∗^*P* < 0.05; ^∗∗^*P* < 0.01; ^∗∗∗^*P* < 0.001, (ns) not significant. The taxon enriched in OB group is marked with OB next to its significant marks, while taxon enriched in WT is marked with WT next to its significant marks

## Discussion

This work aimed to construct a mouse gut microbial biobank (named mGMB in this study) that is publicly available to the academic and medical communities, and to promote culture-dependent studies of GMs in experimental mouse models. To achieve this goal, we worked extensively and laboriously on bacterial isolation and cultivation with *ob/ob* C57BL/6J mice. Using the in-house platform for the isolation, cultivation, and taxonomic characterization of gut microbes, we obtained 1437 cultured bacterial isolates of 126 different species. In our own experience, quite a few of the cultured gut microbial species, especially the slowly growing ones, were rather difficult to revive after cryopreservation in either freeze-dried or glycerol stock. We believe that the physiological status of bacterial cells as well as the procedures used during lyophilization have a great influence on bacterial viability, yet we have not determined the exact key factors. To secure cell viability after preservation in the CGMCC, we adopted a redundant-preservation strategy in this study, and as many as five replicates were used for each bacterial species. Finally, 244 bacterial cellular stocks, representing 126 different species of the mGMB, were successfully deposited into the CGMCC for public use, and their genomes are accessible on various public databases (NCBI, NODE, and gcMeta). In contrast to the mouse gut microbiome study, many large-scale cultivations of human gut microbes were performed^18–22^. Unfortunately, only a small proportion of those cultured gut microbes were further taxonomically characterized and nominated^12,22^. The majority remained taxonomically undefined and unnominated, and there are no 16S rRNA gene sequences representing those microbes in the NCBI or EzBioCloud database^28^. Consequently, these ever-cultured microbes will be repeatedly claimed as new when they are cultured again in later studies. Therefore, in this study, we proposed a simplified protocol (step 5.3, Supplementary Fig. 1) and performed taxonomic characterization and proposed nomenclature for the 77 novel species according to the International Bacteriological Code of Nomenclature^29,30^. Although this study has significantly increased the mouse gut cultured microbial repository, additional work is still needed to further improve the cultivability of GMs. There were 38 taxa that were detected in the initial 96-well plates (Supplementary Fig. 2), but failed to propagate when we attempted to transfer them to the same culture media for large-scale cultivation. Based on the results from BIOLOG tests, we found that four carbon sources (i.e., l-rhamnose, d-fructose, d-galacturonic acid, and d-glucosaminic acid) supported the growth of all 77 new species in the mGMB. Thus, the provision of those carbon sources would possibly promote the recovery of previously uncultured gut microbes.

Both the 77 new species and their genome resources will be very valuable for causative studies of microbe–host interactions and for understanding of metagenomics data. For example, (1) a recent study using bacterial strain P4 from mGMB revealed that *Parabacteroides distasonis* improved host obesity via the modulation of succinate production and secondary bile acid conversion^31^; (2) we also expanded the LTP database^17^ by including the 77 new taxa, and created a customized LTP_mGMB database. As described in the “Results” section, the annotation rate of mouse GM data was improved to ~50% at the genus level and over 24% at the species level, while it was only ~12% at the species level and 33% at the genus level without the customized LTP version_mGMB. In addition, this study supported the concept of core communities and core genomes of GM, which are mostly represented by the identified core species/genera of the mGMB: the top 16–20 genomes covered over 80%, and the entire group of 126 genomes covered over 95% of the KO-based metagenomic functions at the KO level for both OB and WT mice. At the gene level, over 52% and 72% of the predicted genes from the metadata were covered by mGMB genomes when the cutoff values for amino acid sequence identity were set at 60% and 40%, respectively (i.e., 40% is the threshold identity value for the Structural Classification of Proteins (SCOP) database^32,33^, while 60% is the minimum amino acid sequence identity for function conservation^34,35^). In contrast, only 20–30% of the DNA sequence reads of the OB and WT metagenomic data were mapped to the pan-genome represented by the 126 genomes of mGMB. This low coverage (20–30%) at the nucleic acid sequence level and high coverage rate (95%) at the functional KO level remind us that functional redundancy of different DNA sequences must occur universally in GMs. Our function-annotation-based analysis supported the above statement. Actually, high functional redundancy in the microbial ecosystem, including GM, is a well-adopted strategy for resisting against and recovering from temporary disturbances^36–39^.

We found that the *ob/ob* and wild-type C57BL/6J mice shared many gut microbial taxa in this and previous studies^13,40,41^. We also identified phenotype-associated features of GM in *ob/ob* and wild-type C57BL/6J mice in this study. Previous studies of culture-independent metagenomics revealed less-diverse gut microbial communities in mice with metabolic syndrome, a genetic feature of the *ob/ob* mice, compared with controls^42,43^. In addition to the compositional diversity, we observed that the abundances of species are a feature associated with either *ob/ob* or wild-type C57BL/6J mice. The variations in the abundance of these species between the OB and WT groups might hint at phenotype-associated or even metabolic-syndrome-related features. Based on our results and previous findings that quantitative ratio changes of pivotal taxa in the gut microbial community lead to host metabolic problems^44–46^, we propose that both species diversity and population sizes should be monitored for GM homeostasis, which is important for host health. Fecal transplantations with model mice had validated findings that GM played important roles in maintaining healthy metabolism in the host^47,48^; however, the specific microbial contributors are still unidentified. The above 14 bacterial species that had reduced population size in the *ob/ob* mouse GM might be potential contributors to maintaining healthy metabolism, and they might also be as bacterial resources from the mGMB for the formulate of defined preparations for bacterial transplantation. We believe that increasing the number of bacterial strains in the mGMB demonstrates roles in host–microbe interactions.

## Methods

### Sample collection and experimental animal care

Eight-week male wild-type C57BL/6J (*n* = 12) and *ob/ob* (*n* = 12) mice were purchased from the Experimental Animal Center, Chinese Academy of Medical Sciences and euthanized by means of neck dislocation. The animal experiment complied with all ethical regulations for animal testing and research. All experimental procedures were performed in accordance with the Guide for the Care and Use of Laboratory Animals and approved by the Institute of Microbiology, Chinese Academy of Sciences (IMCAS) Ethics Committee. To prevent contamination, the intact cecum was removed from mice and carefully processed in anaerobic workstation to obtain the cecal contents. The cecal contents from *ob/ob* mice (*n* = 12) were used for bacteria isolation. The cecal contents from wild-type C57BL/6J (*n* = 12) and *ob/ob* (*n* = 12) mice resulted in high-throughput metagenomic sequencing. The cecal samples were immediately used for bacterial isolation, and those used for metagenomic sequencing were stored at −80 °C until use.

### Culture media

The broth of MGAM (also known as modified Gifu anaerobe media)^19^ and YCFA media^18^ used in this study were modified by supplementation of 10% rumen liquid^22^. The solid media (agar plate) was the commercially available Wilkins Chalgren (DSMZ medium 339), or the MGAM medium^19^ supplemented with 10% rumen liquid and 5% sheep blood and 1.5% agar^22^.

### Sample treatment and bacterial isolation

Twelve cecal samples from *ob/ob* mice were separated into two groups, one was pretreated with 70% ethanol for 4 h at anaerobic condition^18^. All the cecal samples were suspended in PBS buffer with 0.1% cysteine by vortex, and the large insoluble particles in suspension were removed using cell strainer (BD Falcon, USA). The suspension was further diluted into different concentrations, and 100 μl of each dilution was plated onto agar plates for incubation at 37 °C under an atmosphere of 85% N_2_, 5% CO_2_, and 10% H_2_. The single colonies appearing on the agar plates after incubation for 2, 4, 8, 16, 24, 30, and 45 days were picked. To avoid repeated collection of the same colonies at different times, once a colony was picked, it was circled using a marker accordingly on the back of the Petri dish. The picked colonies were then inoculated into 96-well plates containing 200 μl of broth media in each well. The 96-well plates containing isolates were incubated at 37 °C under an atmosphere of 85% N_2_, 5% CO_2_, and 10% H_2_ for 2–7 days in terms of the growth rate of isolates. Then, 50 μl of the media in each well was collected by centrifugation at 13000 rpm per min for 1 min, and the pellet was lysed with 5 μl of NaOH/SDS lysis buffer (Amresco, USA). The lysed solution was then further diluted by adding 150 μl of deionized water. Two microliters of the above dilution were used for PCR-based amplification of 16S rRNA gene sequences with KOD Fx DNA polymerase (TOYOBO, Japan) using a KOD-recommended PCR program (primers: 27 F: 5′-AGAGTTTGATCCTGGCTCAG-3′; 1492 R: 5′-GGTTACCTTGTTACGACTT-3′). The PCR-amplified 16S rRNA gene sequences were identified using Sanger sequencing by company (TIANYI HUIYUAN Ltd., China). The cultures in the tested wells containing only one species of bacterial cells were enlarged and cultured by inoculation in anaerobic tubes containing 5 ml of liquid media and streaking on agar media plates for further preservation and characterization.

### Selection and cryopreservation of bacterial strains

To ensure that at least 1 strain for each species could be properly recovered after long-term storage in CGMCC, we used a redundant-strain-preservation strategy. The selection criteria of redundant strains were as the following: (1) for the taxa having no less than 5 cultured isolates, we selected 5 isolates to process for the long-term preservation (lyophilization and glycerol stock), (2) for those taxa having less than five cultured isolates, we used all the isolated strains (<5) for further preservation. All the selected isolates were inoculated on agar plates and incubated until the single colonies appearing on the plates. All the colonies on agar plates were collected using cell scraper and suspended in 15% glycerol and 85% bovine serum solution and stored at −80 °C. The glycerol storage of bacteria could be revived again after cryopreservation. About 100 μl of bacteria-containing glycerol was pipetted onto the agar plate and streaked evenly using inoculation loops. Strain information, including culture conditions, is available online at http://www.cgmcc.net/english/mgmb. Detailed morphology, Biolog results, and genomic data can be found within the supplementary information.

### Polyphasic taxonomy analyses of bacterial isolates

The physiological and biochemical features of bacterial isolates were determined with ANI MicroPlates (BIOLOG, the USA), by following the manufacturer’s instruction. Bacterial cell morphology was observed using scanning electron microscope SU010 (Hitachi, Japan) and transmission electron microscope JEM-1400 (JOEL, Japan). Cell motility was examined with light microscopy Axiostar plus 156 (ZEISS, Germany). The phylogeny of isolates was preliminary determined by sequencing their full length of 16S rRNA genes with primers 27F and 1492R and calculating evolutionary distances to close neighbors. For those isolates that showed separated linages, 16S rRNA gene identity was lower than 98% to any known bacterial species, their genomes were sequenced. For each new taxon, the phylogenetic tree was constructed using MEGA 7^49^ with the 16S rRNA gene sequences of the type strains from the phylogenetically close neighboring genus and species.

### Genome sequencing, processing, and DNA data analysis

The genomes of 126 bacterial species in the mGMB were sequenced. Genomic DNAs were extracted using DNeasy Blood & Tissue Kit (QIAGEN, Germany) and sequenced using an Illumina HiSeq 4000 system (Illumina, USA) at the Beijing Genomics Institute (Shenzhen, China). Raw reads of low quality from paired-end sequencing were discarded, and the filtered reads were assembled using SOAPdenovo software v2.04^50^. Gene prediction was performed by glimmer3^51^ with Hidden Markov models. Function annotation was conducted by Blast alignment to the KEGG (Kyoto Encyclopedia of Genes and Genomes) database^52^. The Genome-to-Genome Distance Calculator 2.1 (GGDC)^53^ was used for digital DNA:DNA hybridization (dDDH) of draft genome with its phylogenetically closest genome. The Average Nucleotide Identity (ANI) of genome to genome was calculated using JSpeciesWS^54^. The percentage of conserved proteins (POCP) between each genome and its phylogenetically closest genome was calculated using BLASTp^55^ and was used for taxonomy at genus level. The analysis of 40 single-copy phylogenetic marker genes was performed using specl (http://vm-lux.embl.de/)^56^. The principal coordinate analyses (PCoA) of the functional diversity between genomes were done as described by Bai et al.^57^. The pan-genome of 126 genomes in mGMB was constructed using the Anvi’o v5.0^58^ following the Anvi’o User Tutorial.

### The description and determination criteria of novel taxa

The descriptions of new taxa are based on the analysis of each type strain by performing the following four-aspect analysis of bacterial features: (1) phylogenetic analysis: the 16S rRNA gene sequence identity and the 16S rRNA gene-based phylogenetic tree is recognized as important criterion in taxonomic classification of novel taxa. The phylogenetic tree was constructed by using the neighbor-joining method to depict the phylogenetic distribution and taxonomic relation of each potential new species and its closely related specie; (2) genomic analysis: for each novel taxa, the genome-based analysis includes the calculation of ANI, dDDH, and POCP between each novel taxon and its phylogenetically closest genome and the grouping of each novel taxon based on the 40 single-copy phylogenetic marker genes; (3) physiological analysis: the ANI MicroPlates profiles of type strains provided mainly physiological and biochemical descriptions of each taxon; (4) morphological analysis: according to the microscopy, the shape, size, and presence/absence of flagellum, pilus, or capsule was characterized for each taxon cell and compared with the neighbor species/genus.

For the delineation of new species, there are three golden standards: (1) the 16S rRNA sequence identity <98%, (2) the ANI < 95%, and (3) the dDDH value < 70%. Any taxon meeting the above three criteria simultaneously was defined as new species.

For the delineation of new genera, there are no commonly acknowledged specific standards. Generally, if a new species coincided with at least three out of the following five situations, we considered it as a new genus: (1) the 16S rRNA sequence identity was ≤95%; (2) the new species is clustered on a seperate clade on the phylogenetic tree and the distance between the novel taxon and its neighbor species is greater than that of either two type species from different genus on the phylogenetic tree; (3) the POCP value was <50%; (4) the SpecI grouping analysis suggested that the input genome might originate from a novel genus; (5) there was significant difference in morphology and physiology.

### The 16S rRNA gene sequencing, data collection, and analysis

To understand the gut microbiota compositions of the wild-type (C57BL/6J) and the *ob/ob* mice, the 16S rRNA gene amplicons of *ob/ob* (OB, *n* = 12) and of the wild-type (WT, *n* = 12) mouse cecal samples were amplified from metagenomic DNAs with QIAGEN DNA Stool Mini Kit (QIAGEN, Germany) following the standard protocol as recommended^59^. The V3–V4 regions of 16S rRNA gene were targeted using the primers F341 (5′-CCTACGGGRSGCAGCAG-3′) and R806 (5′-GGACTACVVGGGTATCTAATC-3′) with the barcode, and amplified using SequalPrep™ Long PCR Kit following standard protocol. Amplicons after 30-cycle PCR amplification were then used for the generation of sequencing libraries using Ion Plus Fragment Library Kit (Thermo Fisher, the USA) following the manufacturer’s recommendations. The library was sequenced on an Ion S5 TM XL platform (Thermo Fisher, USA). We got 1,786,648 raw sequence reads in total for the 24 samples, and 74,444 ± 8295 raw reads for each sample on average. The adapters, barcode, low-quality reads, and chimera in the raw data were further filtered using Cutadapt^60^ to achieve 1,682,108 high-quality clean reads in total and 70,088 ± 7681 clean reads for each sample on average. All clean data were further processed using the 64-bit Usearch software^61^ v11 in accordance with the recommended uparse-based pipeline (https://drive5.com/usearch/manual/uparse_pipeline.html)^62^. The OTUs (operational taxonomic units) were clustered at 97% sequence identity, and all the singletons were removed to prevent spurious OTUs. The analysis delivered 1,491,725 quality-controlled and chimera-filtered reads (57,374 ± 7872 per sample) that clustered into 1017 OTUs (568 ± 43 OTUs per sample). The OTUs were annotated with the LTP (Living tree program) database version 132^17^ and the customized LTP_vmGMB database. The LTP_vmGMB database was constructed by supplementation of LTP v132 with 77 16S rRNA gene sequences of the novel species obtained from this study. An OTU table was constructed to include the information of abundance and annotation for each OTU.

The raw data of the 16S rRNA gene amplicons from previous studies were collected from NCBI SRA database (data accessions are available in Supplementary Data 4 and “Data availability” section) for integrated analysis in this study. Those raw data were processed with the same pipeline described in the previous paragraph to obtain the standardized OTU tables. For the pan- and core-genera classification, the relative abundance (RA) of each genus was calculated by dividing the sum of total reads by the genus abundance, while the frequency of occurrence (FO) of each genus was calculated by dividing the total number of analyzed samples by the number of samples containing such genus (i.e., when a genus presenting in all samples, its FO = 100%, while a genus presenting in none of the samples, the FO = 0). The pan-genera were defined with threshold values of FO > 80% and RA > 0.1%, and the core-genera were defined with criterion of FO > 5%. The coverages of 16S rRNA gene amplicons of mGMB or 77 novel species at OTU level were achieved by local BLASTn^63^ analysis of metagenomics OTU sequences against the 16S rRNA sequences of mGMB or the novel species with identity >97%.

### Metagenome sequencing, processing, and analysis

Metagenomic DNAs of *ob/ob* (*n* = 6) and C57BL/6J mice (*n* = 6) cecal samples were extracted as described above. A total amount of 1 μg of DNA per sample was used as input material for sample preparations. Sequencing libraries were generated using NEBNext^®^ Ultra™ DNA Library Prep Kit for Illumina (NEB, USA), and index codes were added to attribute sequences to each sample. Briefly, the DNA sample was fragmented by sonication to a size of 350 bp; then DNA fragments were end-polished, A-tailed, and ligated with the full-length adapter for Illumina sequencing. After amplification, PCR products were purified (AMPure XP system) and libraries were analyzed for size distribution by Agilent2100 Bioanalyzer and quantified using real-time PCR. The clustering of the index-coded samples was performed on a cBot Cluster Generation stem according to the manufacturer’s instructions. After cluster generation, the library preparations were sequenced on an Illumina HiSeq platform and paired-end reads were generated. Raw data were conducted using Readfq V8 (https://github.com/cjfields/readfq) to acquire clean data. The clean data were blast to the host database using Bowtie2.2.4 software^64^ to filter the reads that are of host origin, and then were assembled with MEGAHIT software v1.1.2^65^. The assembled scaffolds with length >500 bp were used for ORF prediction using MetaGeneMark^66^. The predicted ORFs were then used for generating the nonredundant genes with CD-HIT software v4.5.8^67^, and the clean reads was mapped to the nonredundant unique genes using Bowtie v2.2.4^64^ to finally generate the nonredundant unique gene catalogs containing gene-abundant information. The pan-genome of mGMB was assembled by merging 126 genomes with CD-HIT software v4.5.8^67^ and then indexed into a database using Bowtie v2.2.4^64^. The short reads mapping to the pan-genome were performed with Bowtie v2.2.4^64^ and the statistical summarization of mapping results was performed using SAMtools v0.1.19^68^. Further analysis and visualization of the mapping results were conducted with the Anvi’o v5.0^58^ following the Anvi’o user tutorial for metagenomic workflow. We analyze the coverage of mGMB pan-genome to the nonredundant gene catalog of metagenomes. The genes in mGMB pan-genome were transferred into amino acid sequences using Prokka v1.13.3^69^. Then the BLASTp analysis of nonredundant unique genes (amino acid sequences) was performed against the mGMB pan-genomes, with a defined parameter of -outfmt 7 -evalue 0.00001 -qcov_hsp_perc 50. The coverage rate of mGMB genomes to the metagenomic gene catalog was calculated twice with two different cutoff values of the amino acid sequence identity-60% and 40%, respectively. Such calculations were based on the fact that 40% was the threshold identity value of Structural Classification of Proteins (SCOP)^32,33^, while 60% was the minimum amino acid sequence identity for function conservation^34,35^. All above analyses were performed using default parameters of the software unless otherwise stated.

Function annotation was conducted by BLAST alignment to the KEGG (Kyoto Encyclopedia of Genes and Genomes) database^52^. The distributional specificity of KOs between *ob/ob* and wild-type samples was analyzed by comparison of the KO abundance with *t* test. For calculation of coverage (%), the KO profiles of metagenomic and bacterial genomic data were tabularized in the form of presence/absence binary code (0/1). The cumulative curve was constructed using custom scripts written in R.

### Statistical analysis

All analyses were performed using IBM SPSS Statistics 20. All the box–whisker plots and bar charts were generated using Graphpad Prism v6^70^. Comparison of two groups of data was statistically evaluated with *t* test unless otherwise stated. *P* < 0.05 was considered being statistically significant. All results were expressed in the form of mean ± SEM unless indicated otherwise. The boxplots showed the median values and whiskers extending to include all the valid data denoted by Turkey test. All figures showed data from at least three biological replicates.

### Reporting summary

Further information on research design is available in the Nature Research Reporting Summary linked to this article.

## Supplementary information

Supplementary Information

Description of Additional Supplementary Files

Supplementary Data 1

Supplementary Data 2

Supplementary Data 3

Supplementary Data 4

Supplementary Data 5

Reporting Summary

## Data Availability

The datasets generated and analyzed in this study are available as the following: the raw data of 16S rRNA gene amplicons are deposited in NCBI SRA (Accession: SRR8077557-80). All the genomic and metagenomic data obtained in this study are available at NODE with the project accession OEP000211, NCBI under Project PRJNA486904, and gcMeta under Project NMDC10010898. The GeneBank IDs of the 16S rRNA gene sequences of all taxa in mGMB are MK287622–MK287775 and MN081616–MN081733. The other datasets analyzed in this study were available at NCBI with accessions of PRJNA486904, PRJNA400789, PRJNA418420, PRJNA417284, PRJNA474117, PRJEB11650, PRJNA393083, PRJNA388263, PRJNA508548, PRJNA453406, PRJDB4202, and at iMGMC. The source data underlying Figs. 1, 2, 3, 4, and Table 1 are provided as a Source Data file.

## Source data

Source Data
