# Supplementary material for: The Mouse Gut Microbial Biobank expands the coverage of cultured bacteria
Source: Nat Commun. 2020 Jan 7;11:79. doi: 10.1038/s41467-019-13836-5 (PMC6946648; doi:10.1038/s41467-019-13836-5)
Supplement: Supplementary file 1 — Supplementary Information [file 41467_2019_13836_MOESM1_ESM.pdf]

# The Mouse Gut Microbial Biobank expands the coverage of cultured bacteria

Liu et al.

# Supplementary Figures

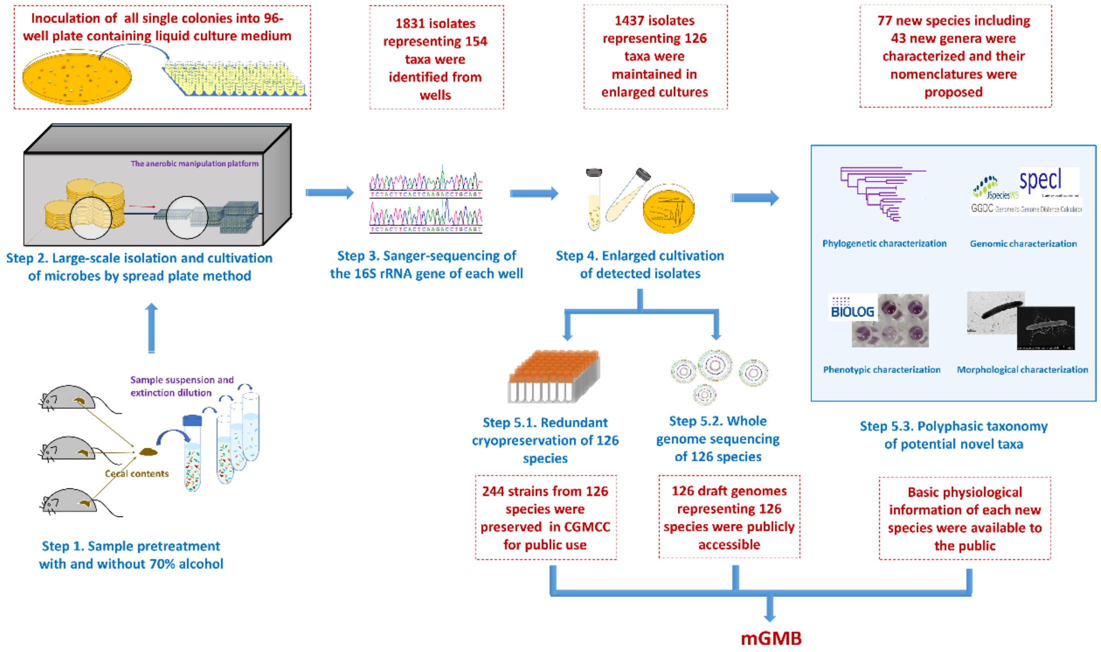


**Supplementary Figure 1. The workflow for the large-scale cultivation and characterization of mouse gut microbes.** The working steps were numbered from Step 1 to 5.3. The main outcomes of each step were shown on top of the panel and in red dashed box.

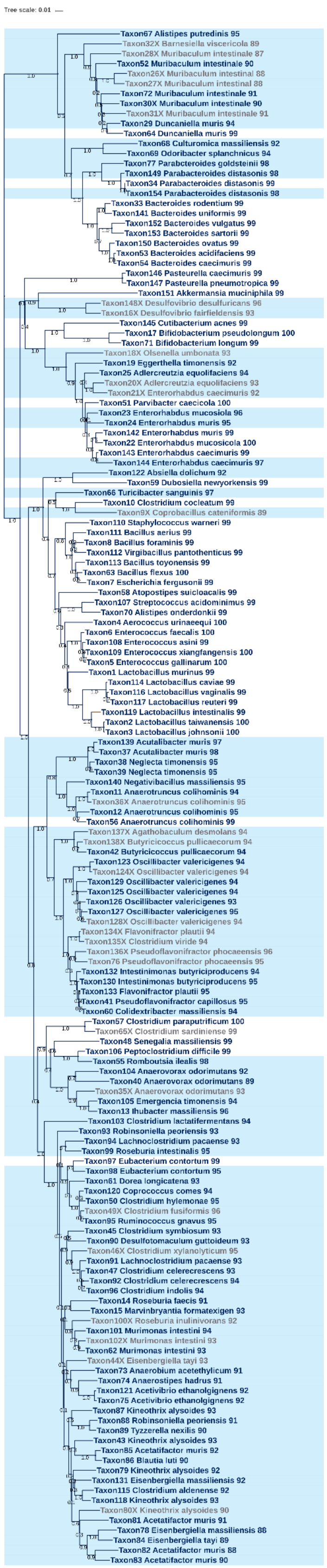


**Supplementary Figure 2. The Neighbour-joining phylogenetic tree of 154 isolated taxa based on 16S rRNA gene sequences.** The 154 bacterial taxa were clustered from 1831 isolates based on the 16S rRNA gene identity and by applying a cut-off value of 98% for different taxa. The closely related neighbor species of each taxon and its 16S rRNA gene sequence identity was listed next to the taxon number. Bootstrap value was 1000. Bar: 0.01 substitutions per nucleotide position.

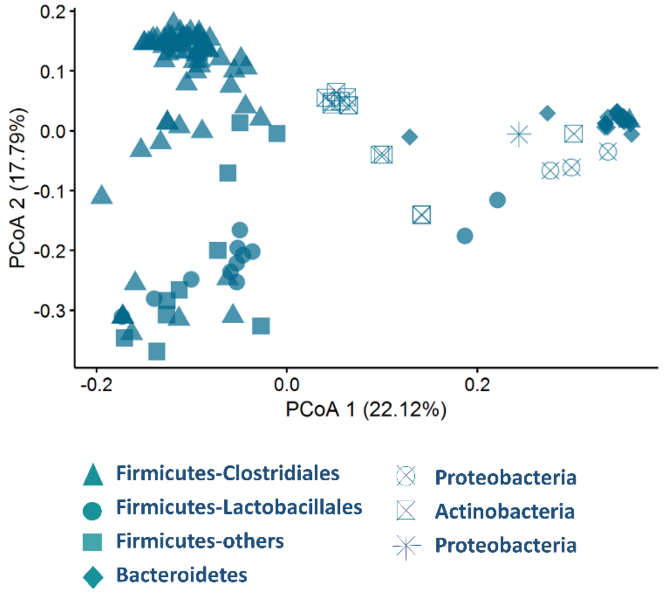


**Supplementary Figure 3. The PCoA plot of genomic diversity within the 126 cultured taxa.** Each Point represents a KO (KEGG ORTHOLOGY) profile of a genome. Symbols are explained the panel.

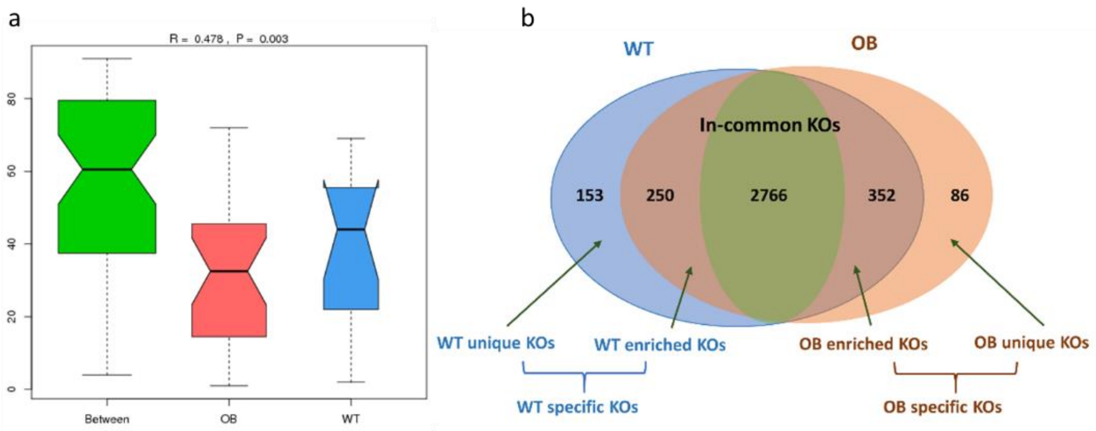


**Supplementary Figure 4. The genotype-associated difference of metagenomic functions.** OB: KEGG Ortholog (KO) pool of ob/ob mouse GMs (n=6); WT: KO pool of C57BL/6 mouse GMs (n=6). **a,** the KO-based ANOSIM analysis of difference between WT and OB. *P* values were obtained by t-test, box-and-whiskers plot: centre: median, bounds of box: quartile, whiskers: extreme. **b,** the Venn diagram indicating the distribution of annotated KOs in OB and WT. Blue ellipse: KO pool for WT; Orange ellipse: KO pool for OB; Green ellipse: in-common KOs for both groups.

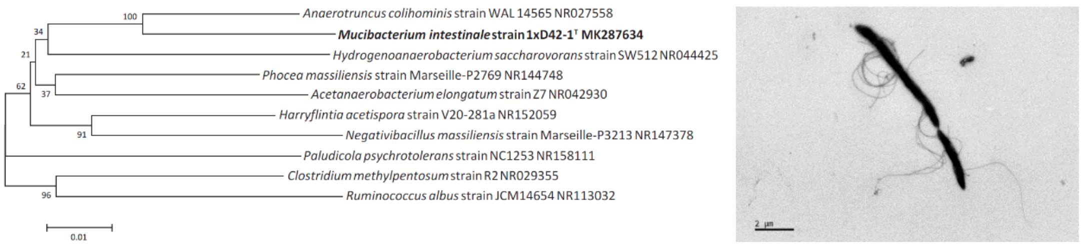


Supplementary Figure 5. The Neighbour-joining phylogenetic tree based on 16S rRNA gene sequences (left, a) and the TEM-based TEM-based cell morphologic images (right, b) of strain D42-8$^{T}$. GenBank accession numbers are given in parentheses. Percentages of bootstrap support are shown at branch nodes. Bootstrap value was 1000. Bar: 0.01 substitutions per nucleotide position.

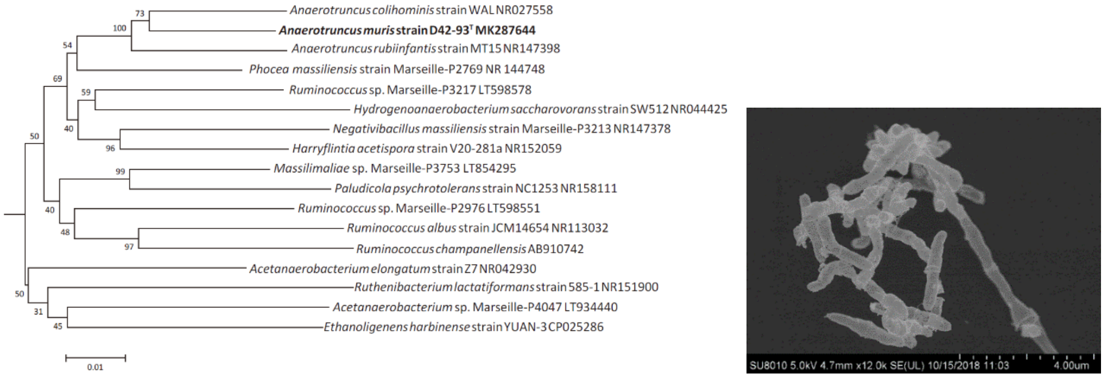


Supplementary Figure 6. The Neighbour-joining phylogenetic tree (left, a) based on 16S rRNA gene sequence and TEM-based cell morphologic images (right, b) of strain D42-93$^{T}$. GenBank accession numbers are given in parentheses. Percentages of bootstrap support are shown at branch nodes. Bootstrap value is 1000. Bar: 0.01 substitutions per nucleotide position.

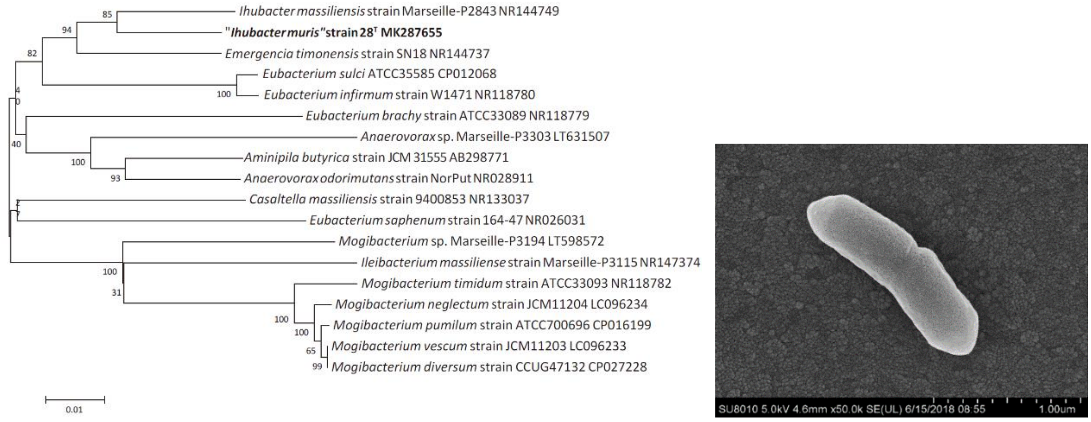


Supplementary Figure 7. The Neighbour-joining phylogenetic tree (left, a) based on 16S rRNA gene sequence and TEM-based cell morphologic images (right, b) of strain 28$^{T}$. GenBank accession numbers are given in parentheses. Percentages of bootstrap support are shown at branch nodes. Bootstrap value is 1000. Bar: 0.01 substitutions per nucleotide position.

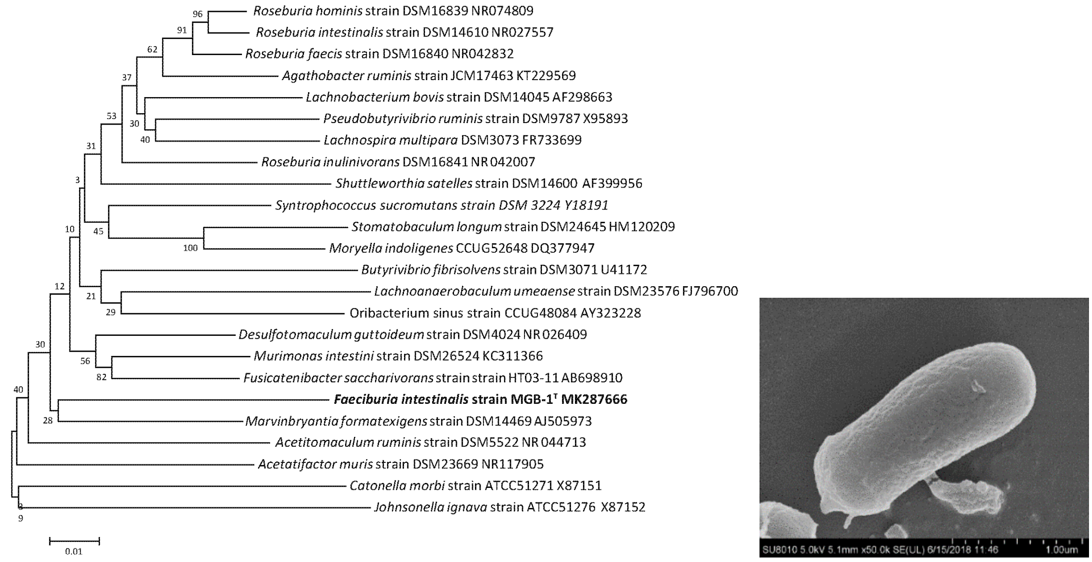


Supplementary Figure 8. The Neighbour-joining phylogenetic tree based on 16S rRNA gene sequences (left, a) and the TEM-based cell morphologic images (right, b) of strain MGB-1$^T$. GenBank accession numbers are given in parentheses. Percentages of bootstrap support are shown at branch nodes. Bootstrap value was 1000. Bar: 0.01 substitutions per nucleotide position.

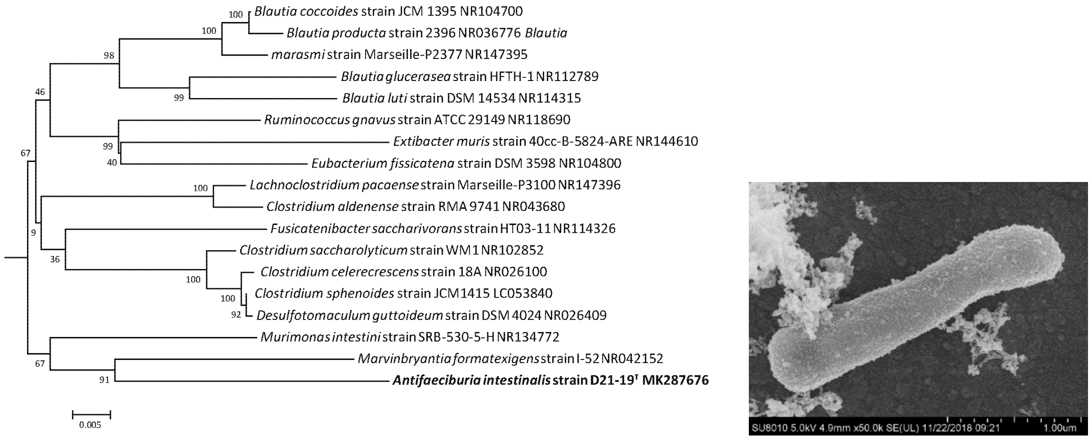


Supplementary Figure 9. The Neighbour-joining phylogenetic tree based on 16S rRNA gene sequences (left, a) and the TEM-based cell morphologic images (right, b) of strain D21-19$^T$. GenBank accession numbers are given in parentheses. Percentages of bootstrap support are shown at branch nodes. Bootstrap value was 1000. Bar: 0.01 substitutions per nucleotide position.

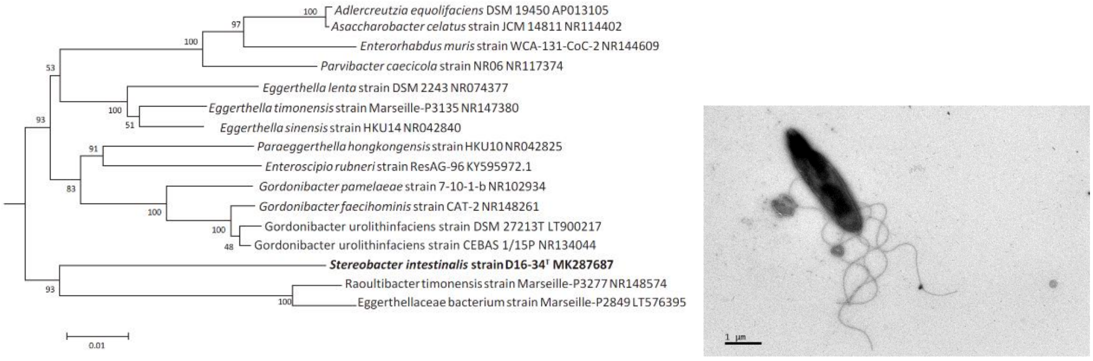


Supplementary Figure 10. The Neighbour-joining phylogenetic tree based on 16S rRNA gene sequences (left, a) and the TEM-based cell morphologic images (right, b) of strain D16-34$^T$.

GenBank accession numbers are given in parentheses. Percentages of bootstrap support are shown at branch nodes. Bootstrap value was 1000. Bar: 0.01 substitutions per nucleotide position.

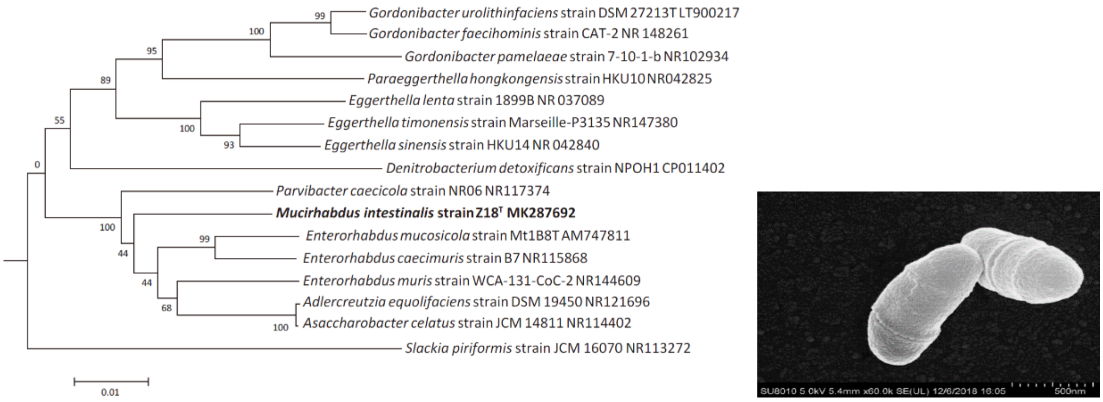


Supplementary Figure 11. The Neighbour-joining phylogenetic tree based on 16S rRNA gene sequences (left, a) and the TEM-based cell morphologic images (right, b) of strain P55$^{T}$. GenBank accession numbers are given in parentheses. Percentages of bootstrap support are shown at branch nodes. Bootstrap value was 1000. Bar: 0.01 substitutions per nucleotide position.

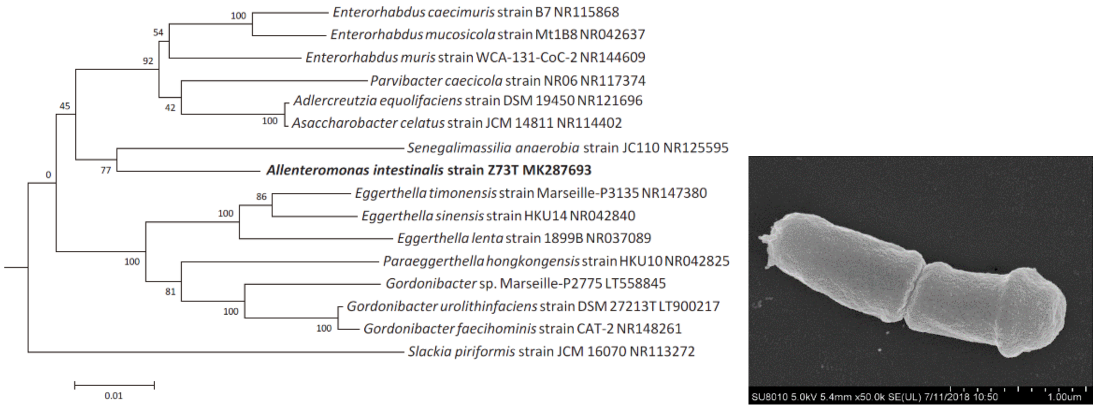


Supplementary Figure 12. The Neighbour-joining phylogenetic tree based on 16S rRNA gene sequences (left, a) and the TEM-based cell morphologic images (right, b) of strain Z73$^{T}$. GenBank accession numbers are given in parentheses. Percentages of bootstrap support are shown at branch nodes. Bootstrap value was 1000. Bar: 0.01 substitutions per nucleotide position.

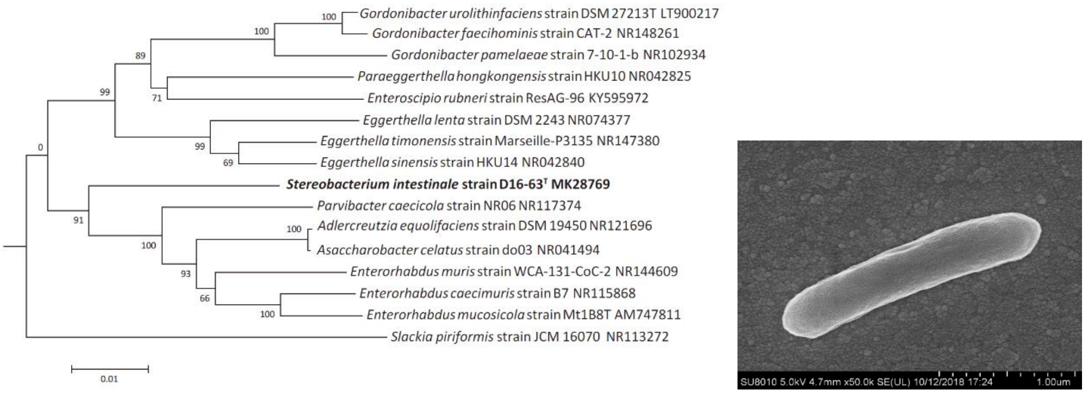


Supplementary Figure 13. The Neighbour-joining phylogenetic tree based on 16S rRNA gene sequences (left, a) and the TEM-based cell morphologic images (right, b) of strain D16-63$^{T}$.

GenBank accession numbers are given in parentheses. Percentages of bootstrap support are shown at branch nodes. Bootstrap value was 1000. Bar: 0.01 substitutions per nucleotide position.

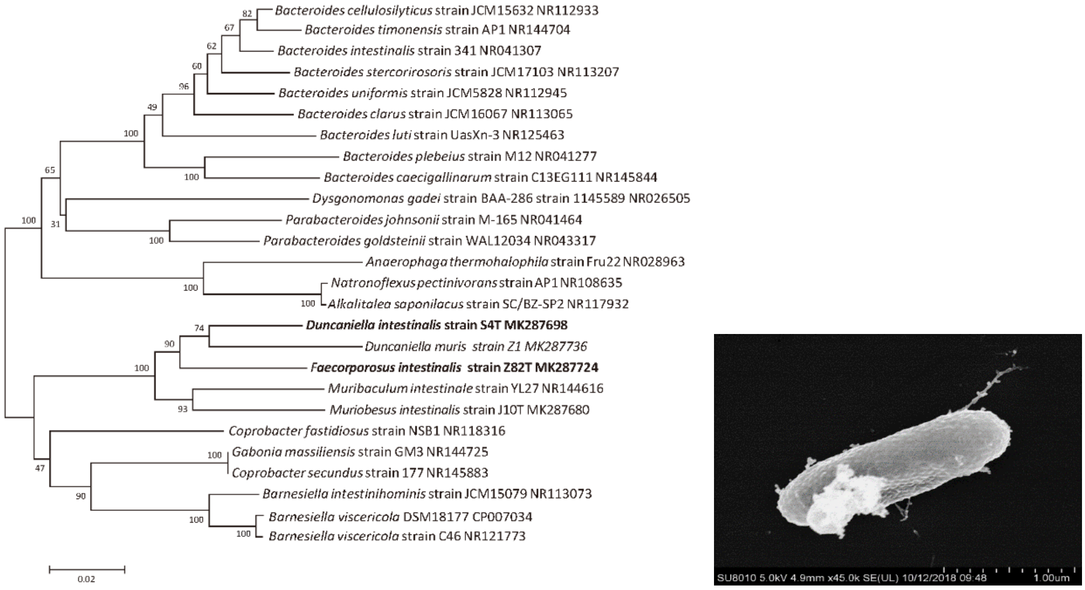


Supplementary Figure 14. The Neighbour-joining phylogenetic tree based on 16S rRNA gene sequences (left, a) and the TEM-based cell morphologic images (right, b) of strain S4$^T$. GenBank accession numbers are given in parentheses. Percentages of bootstrap support are shown at branch nodes. Bootstrap value was 1000. Bar: 0.01 substitutions per nucleotide position.

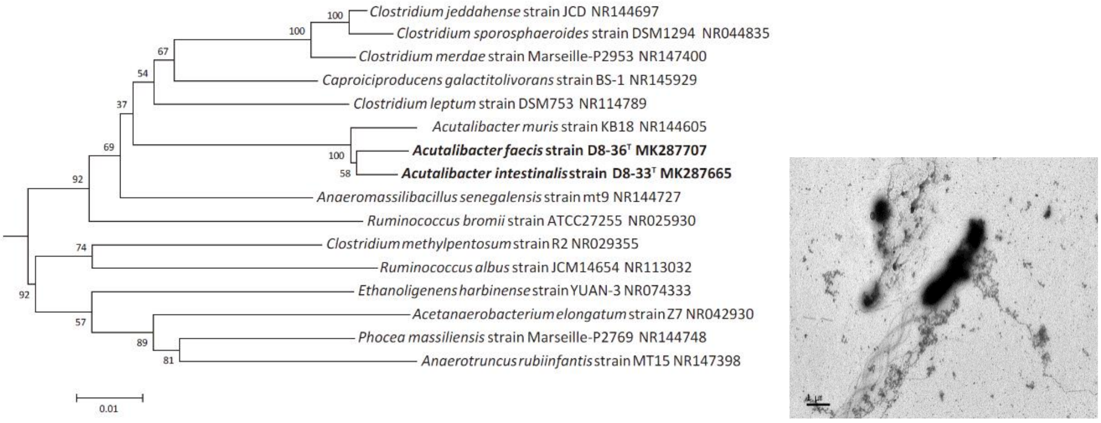


Supplementary Figure 15. The Neighbour-joining phylogenetic tree (left, a) based on 16S rRNA gene sequence and TEM-based cell morphologic images (right, b) of strain D8-36$^T$. GenBank accession numbers are given in parentheses. Percentages of bootstrap support are shown at branch nodes. Bootstrap value is 1000. Bar: 0.01 substitutions per nucleotide position.

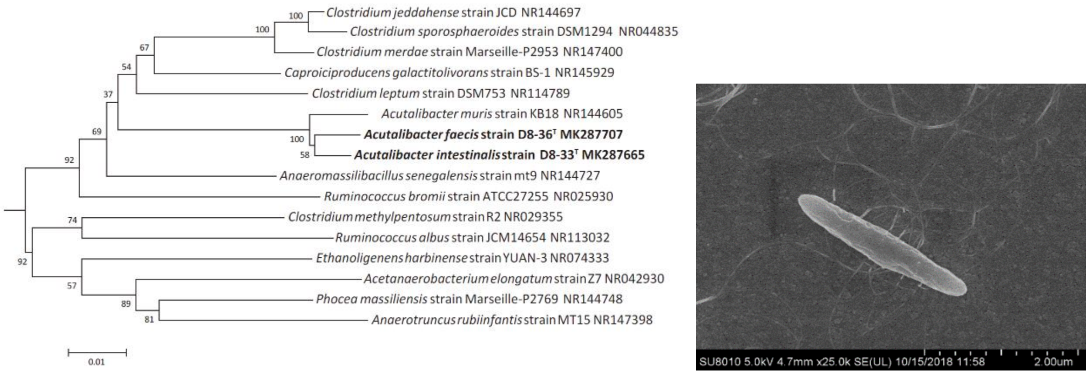


Supplementary Figure 16. The Neighbour-joining phylogenetic tree (left, a) based on 16S rRNA gene sequence and TEM-based cell morphologic images (right, b) of strain D8-33$^{T}$. GenBank accession numbers are given in parentheses. Percentages of bootstrap support are shown at branch nodes. Bootstrap value is 1000. Bar: 0.01 substitutions per nucleotide position.

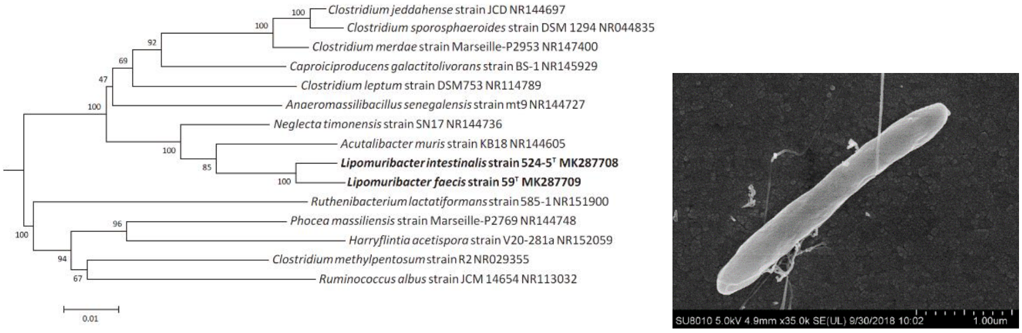


Supplementary Figure 17. The Neighbour-joining phylogenetic tree based on 16S rRNA gene sequences (left, a) and the TEM-based cell morphologic images (right, b) of strain 524-5$^{T}$. GenBank accession numbers are given in parentheses. Percentages of bootstrap support are shown at branch nodes. Bootstrap value was 1000. Bar: 0.01 substitutions per nucleotide position.

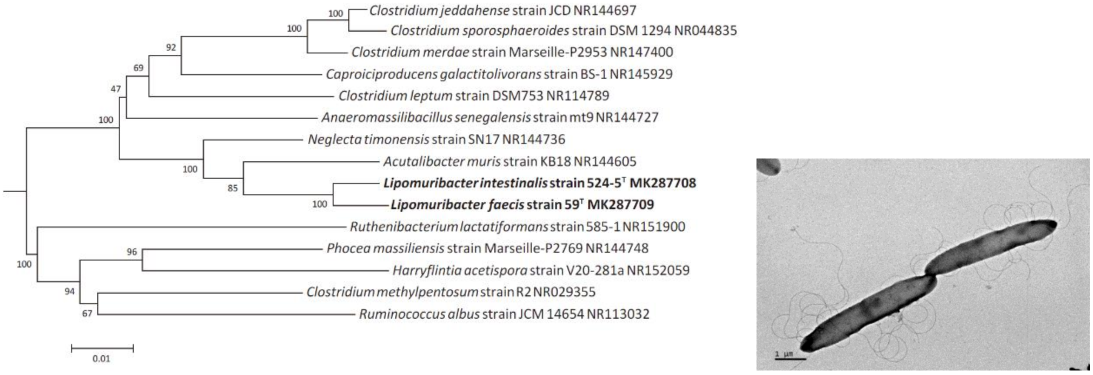


Supplementary Figure 18. The Neighbour-joining phylogenetic tree (left, a) based on 16S rRNA gene sequence and TEM-based cell morphologic images (right, b) of strain 59$^{T}$. GenBank accession numbers are given in parentheses. Percentages of bootstrap support are shown at branch nodes. Bootstrap value is 1000. Bar: 0.01 substitutions per nucleotide position.

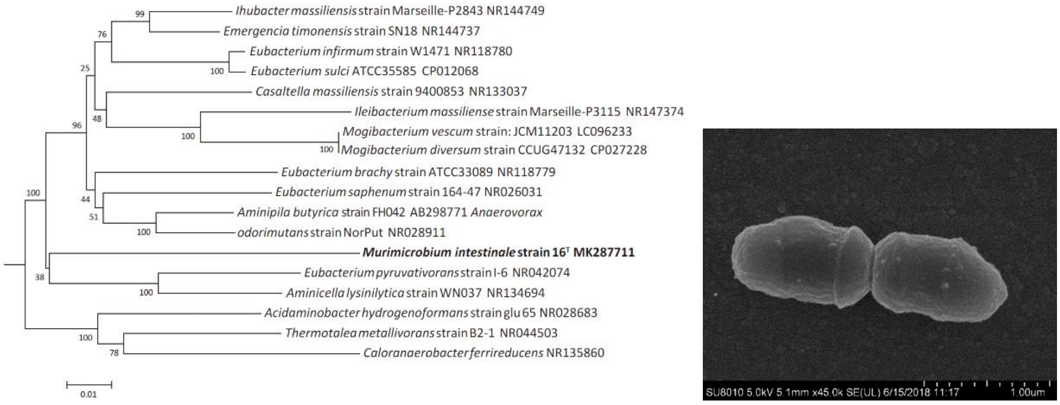


Supplementary Figure 19. The Neighbour-joining phylogenetic tree based on 16S rRNA gene sequences (left, a) and the TEM-based cell morphologic images (right, b) of strain 16$^{T}$. GenBank accession numbers are given in parentheses. Percentages of bootstrap support are shown at branch nodes. Bootstrap value was 1000. Bar: 0.01 substitutions per nucleotide position.

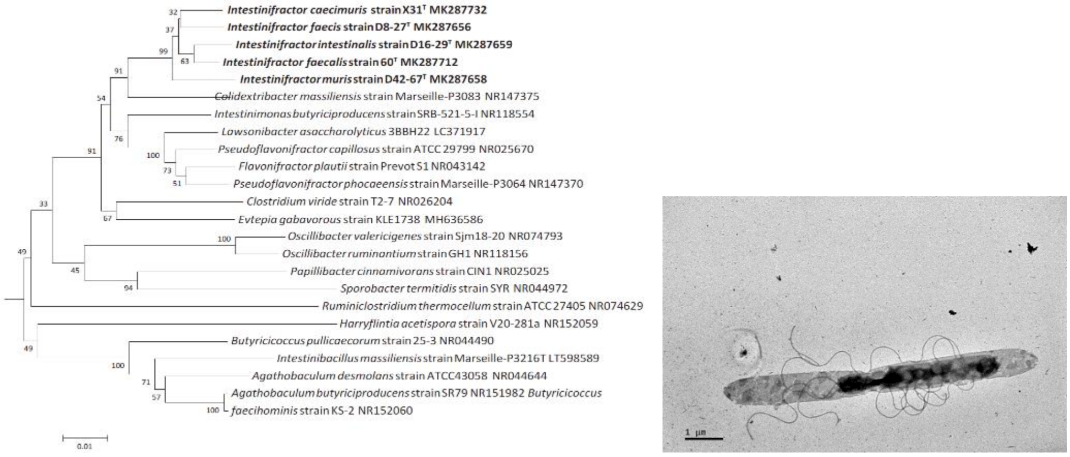


Supplementary Figure 20. The Neighbour-joining phylogenetic tree based on 16S rRNA gene sequences (left, a) and the TEM-based cell morphologic images (right, b) of strain D16-29$^{T}$. GenBank accession numbers are given in parentheses. Percentages of bootstrap support are shown at branch nodes. Bootstrap value was 1000. Bar: 0.01 substitutions per nucleotide position.

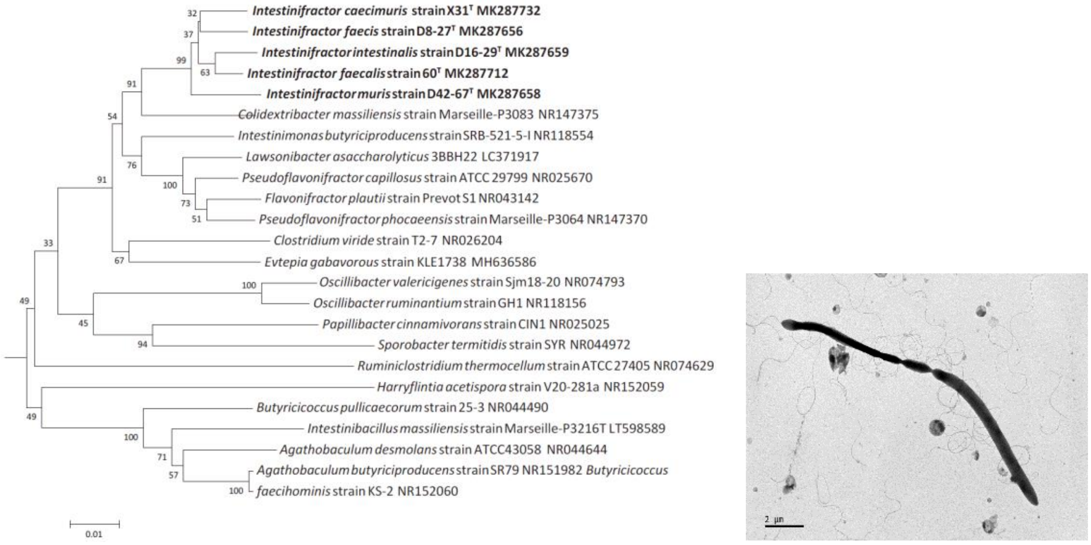

Supplementary Figure 21. The Neighbour-joining phylogenetic tree (left, a) based on 16S rRNA gene sequence and TEM-based cell morphologic images (right, b) of strain 60$^{T}$. GenBank accession numbers are given in parentheses. Percentages of bootstrap support are shown at branch nodes. Bootstrap value is 1000. Bar: 0.01 substitutions per nucleotide position.

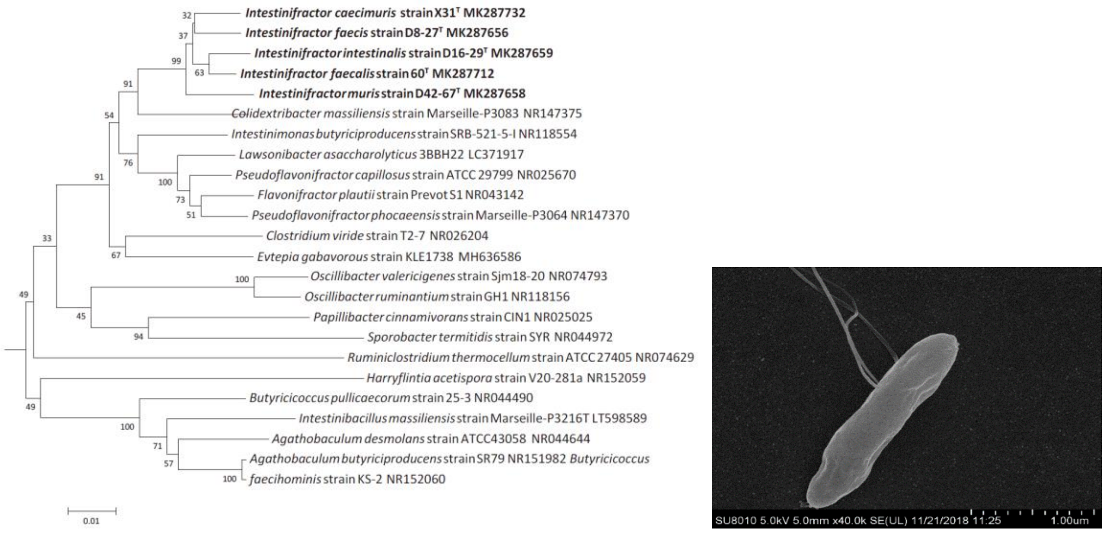


Supplementary Figure 22. The Neighbour-joining phylogenetic tree (left, a) based on 16S rRNA gene sequence and TEM-based cell morphologic images (right, b) of strain X31$^{T}$. GenBank accession numbers are given in parentheses. Percentages of bootstrap support are shown at branch nodes. Bootstrap value is 1000. Bar: 0.01 substitutions per nucleotide position.

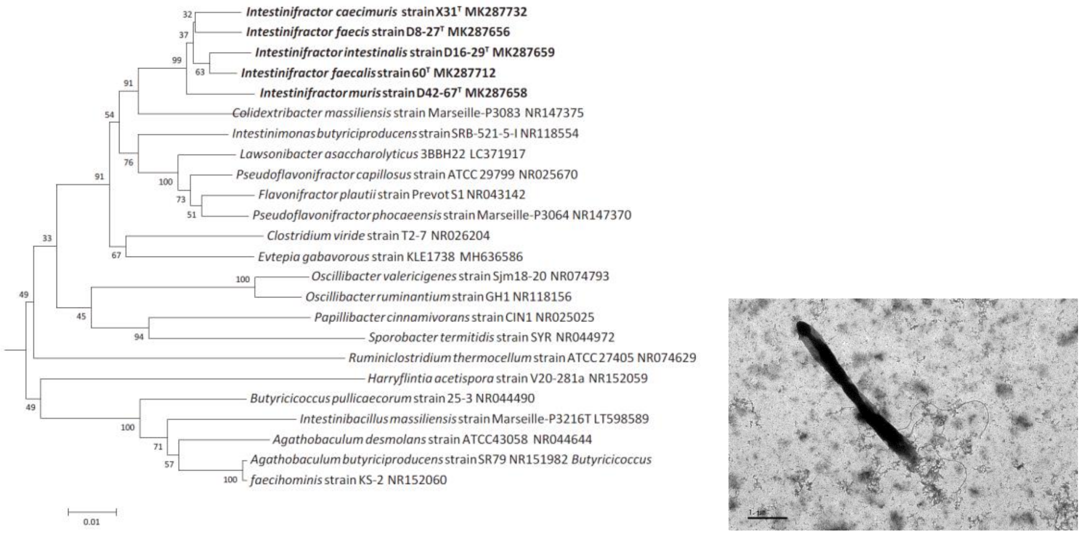


Supplementary Figure 23. The Neighbour-joining phylogenetic tree (left, a) based on 16S rRNA gene sequence and TEM-based cell morphologic images (right, b) of strain D8-27$^{T}$. GenBank accession numbers are given in parentheses. Percentages of bootstrap support are shown at branch nodes. Bootstrap value is 1000. Bar: 0.01 substitutions per nucleotide position.

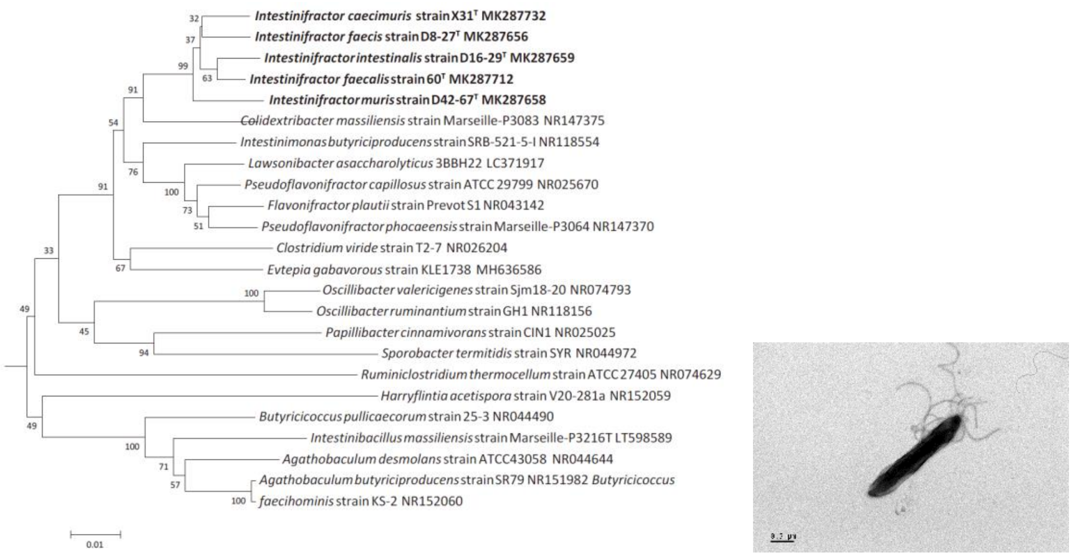


Supplementary Figure 24. The Neighbour-joining phylogenetic tree (left, a) based on 16S rRNA gene sequence and TEM-based cell morphologic images (right, b) of strain D42-67$^{T}$. GenBank accession numbers are given in parentheses. Percentages of bootstrap support are shown at branch nodes. Bootstrap value is 1000. Bar: 0.01 substitutions per nucleotide position.

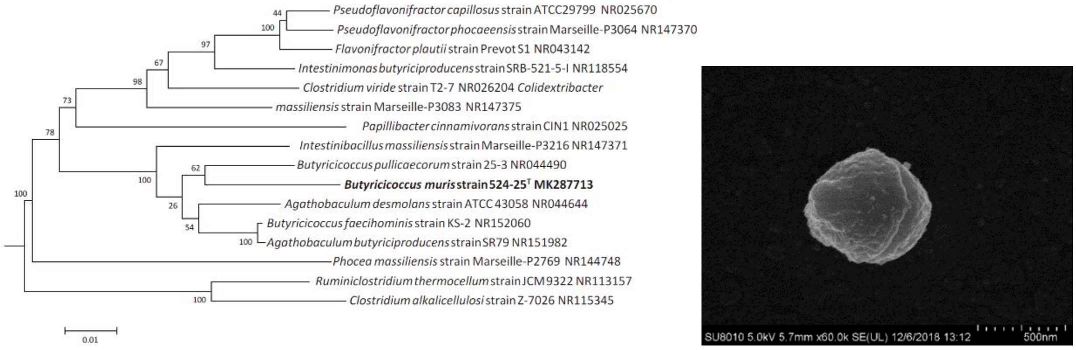


Supplementary Figure 25. The Neighbour-joining phylogenetic tree (left, a) based on 16S rRNA gene sequence and TEM-based cell morphologic images (right, b) of strain 524-25$^{T}$. GenBank accession numbers are given in parentheses. Percentages of bootstrap support are shown at branch nodes. Bootstrap value is 1000. Bar: 0.01 substitutions per nucleotide position.

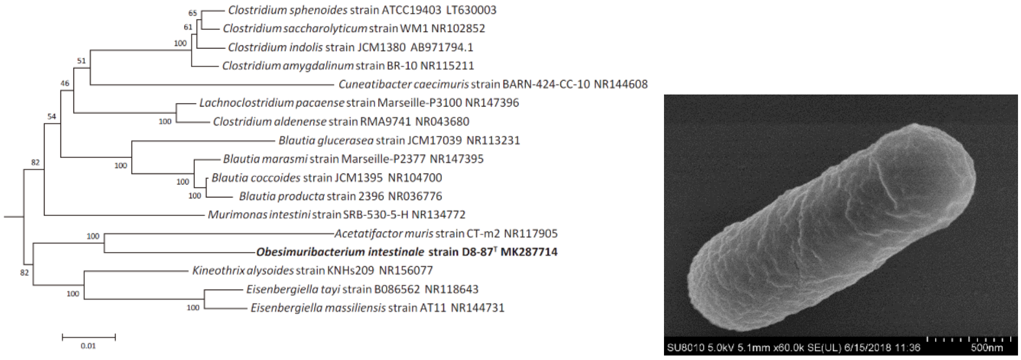


Supplementary Figure 26. The Neighbour-joining phylogenetic tree based on 16S rRNA gene sequences (left, a) and the TEM-based cell morphologic images (right, b) of strain D8-87$^{T}$. GenBank accession numbers are given in parentheses. Percentages of bootstrap support are shown at branch nodes. Bootstrap value was 1000. Bar: 0.01 substitutions per nucleotide position.

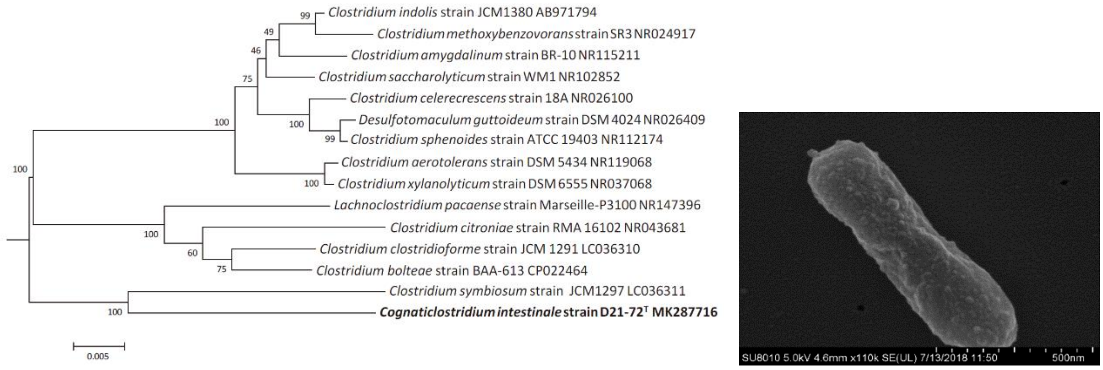


Supplementary Figure 27. The Neighbour-joining phylogenetic tree based on 16S rRNA gene sequences (left, a) and the TEM-based cell morphologic images (right, b) of strain D21-72$^{T}$. GenBank accession numbers are given in parentheses. Percentages of bootstrap support are shown at branch nodes. Bootstrap value was 1000. Bar: 0.005 substitutions per nucleotide position.

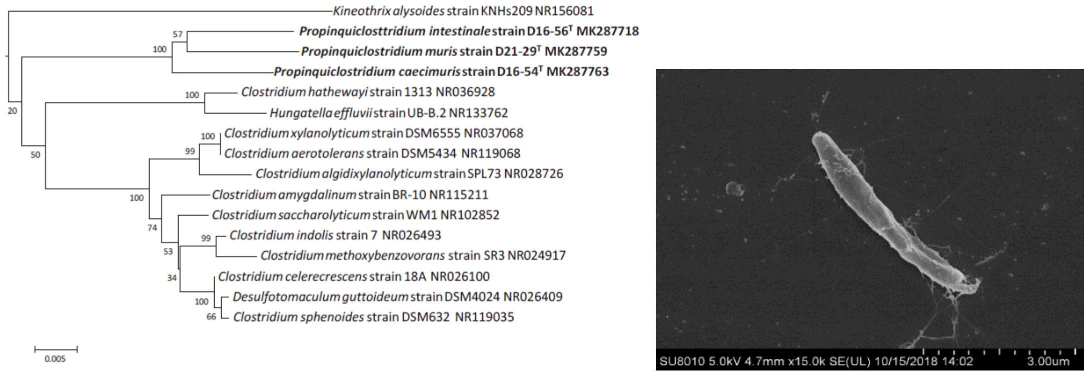


Supplementary Figure 28. The Neighbour-joining phylogenetic tree based on 16S rRNA gene sequences (left, a) and the TEM-based cell morphologic images (right, b) of strain D16-56$^{T}$. GenBank accession numbers are given in parentheses. Percentages of bootstrap support are shown at branch nodes. Bootstrap value was 1000. Bar: 0.005 substitutions per nucleotide position.

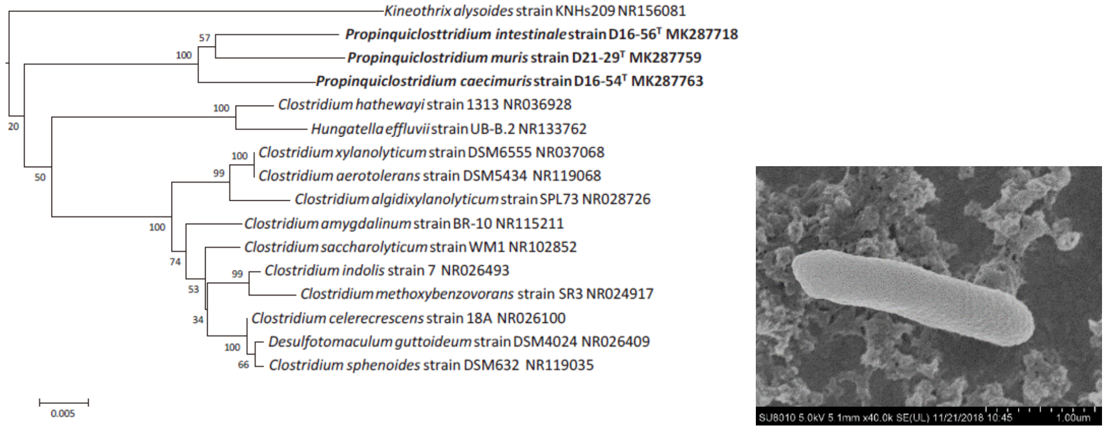


Supplementary Figure 29. The Neighbour-joining phylogenetic tree (left, a) based on 16S rRNA gene sequence and TEM-based cell morphologic images (right, b) of strain D21-29$^{T}$. GenBank accession numbers are given in parentheses. Percentages of bootstrap support are shown at branch nodes. Bootstrap value is 1000. Bar: 0.01 substitutions per nucleotide position.

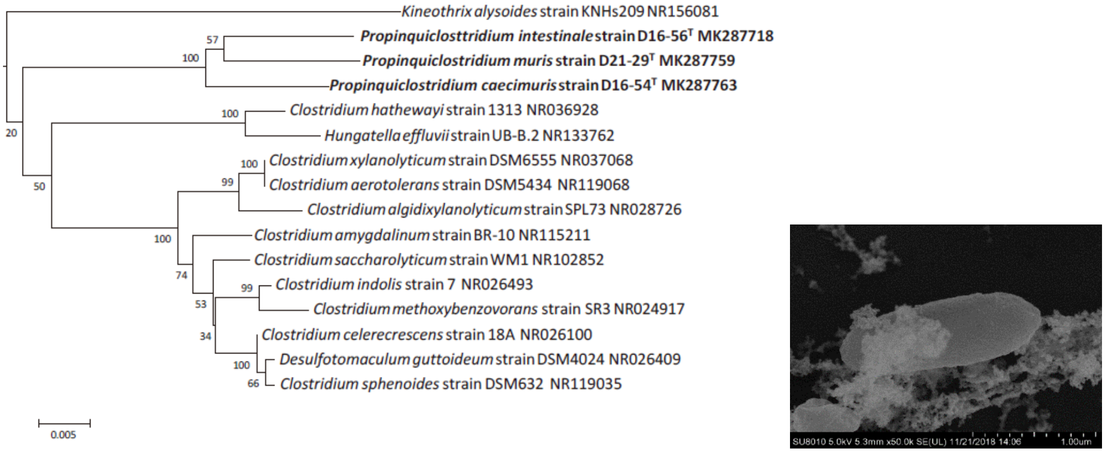


Supplementary Figure 30. The Neighbour-joining phylogenetic tree (left, a) based on 16S rRNA gene sequence and TEM-based cell morphologic images (right, b) of strain D16-54$^{T}$. GenBank accession numbers are given in parentheses. Percentages of bootstrap support are shown at branch nodes. Bootstrap value is 1000. Bar: 0.005 substitutions per nucleotide position.

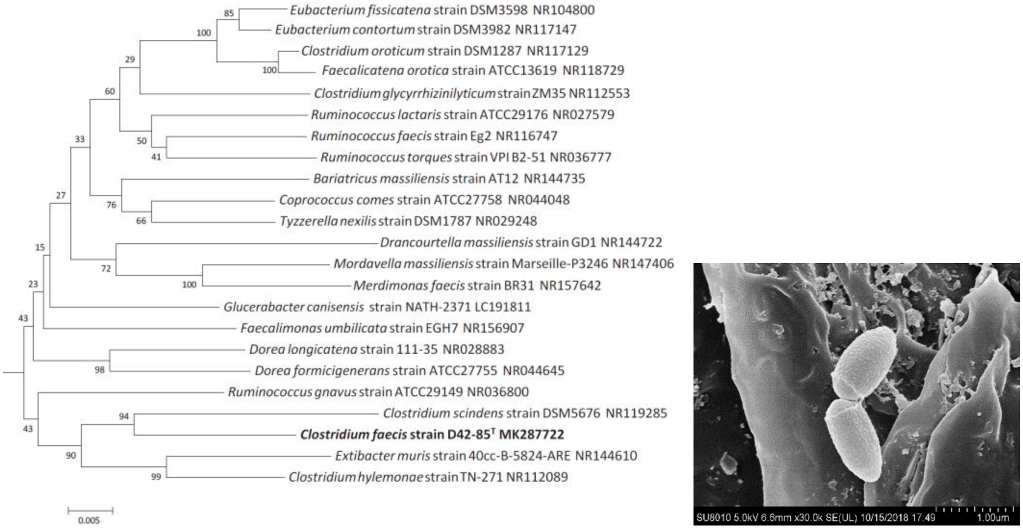


Supplementary Figure 31. The Neighbour-joining phylogenetic tree based on 16S rRNA gene sequences (left, a) and the TEM-based cell morphologic images (right, b) of strain D42-85$^{T}$. GenBank accession numbers are given in parentheses. Percentages of bootstrap support are shown at branch nodes. Bootstrap value was 1000. Bar: 0.005 substitutions per nucleotide position.

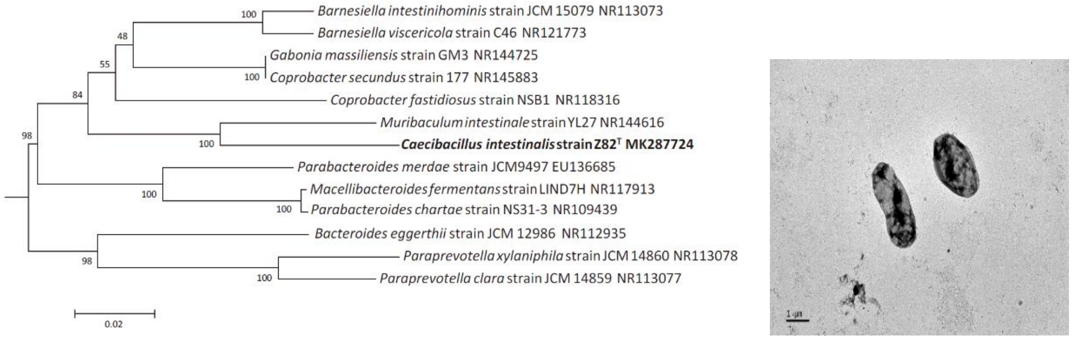


Supplementary Figure 32. The Neighbour-joining phylogenetic tree based on 16S rRNA gene sequences (left, a) and the TEM-based cell morphologic images (right, b) of strain Z82$^{T}$. GenBank

accession numbers are given in parentheses. Percentages of bootstrap support are shown at branch nodes. Bootstrap value was 1000. Bar: 0.002 substitutions per nucleotide position.

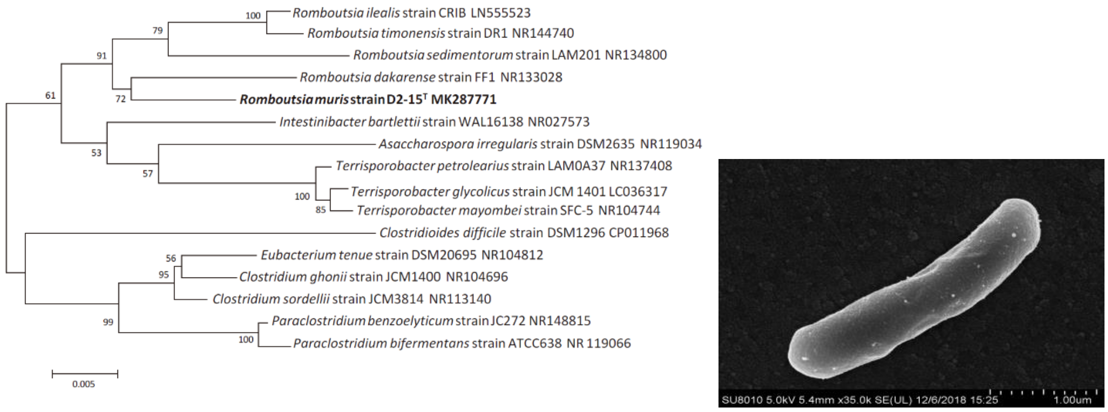


Supplementary Figure 33. The Neighbour-joining phylogenetic tree (left, a) based on 16S rRNA gene sequence and TEM-based cell morphologic images (right, b) of strain D2-15$^T$. GenBank accession numbers are given in parentheses. Percentages of bootstrap support are shown at branch nodes. Bootstrap value is 1000. Bar: 0.005 substitutions per nucleotide position.

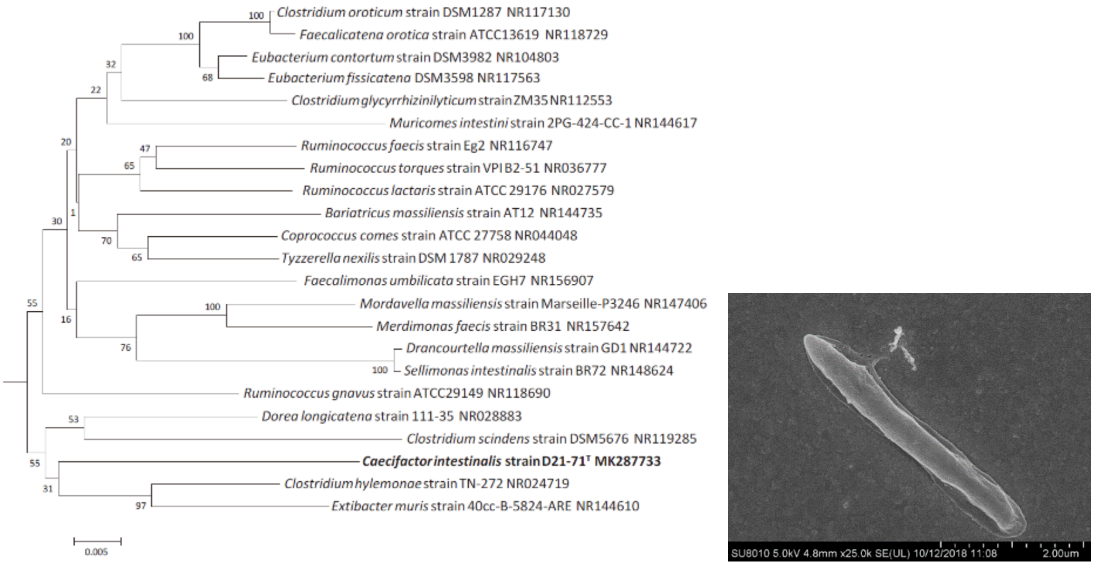


Supplementary Figure 34. The Neighbour-joining phylogenetic tree based on 16S rRNA gene sequences (left, a) and the TEM-based cell morphologic images (right, b) of strain D21-71$^T$. GenBank accession numbers are given in parentheses. Percentages of bootstrap support are shown at branch nodes. Bootstrap value was 1000. Bar: 0.005 substitutions per nucleotide position.

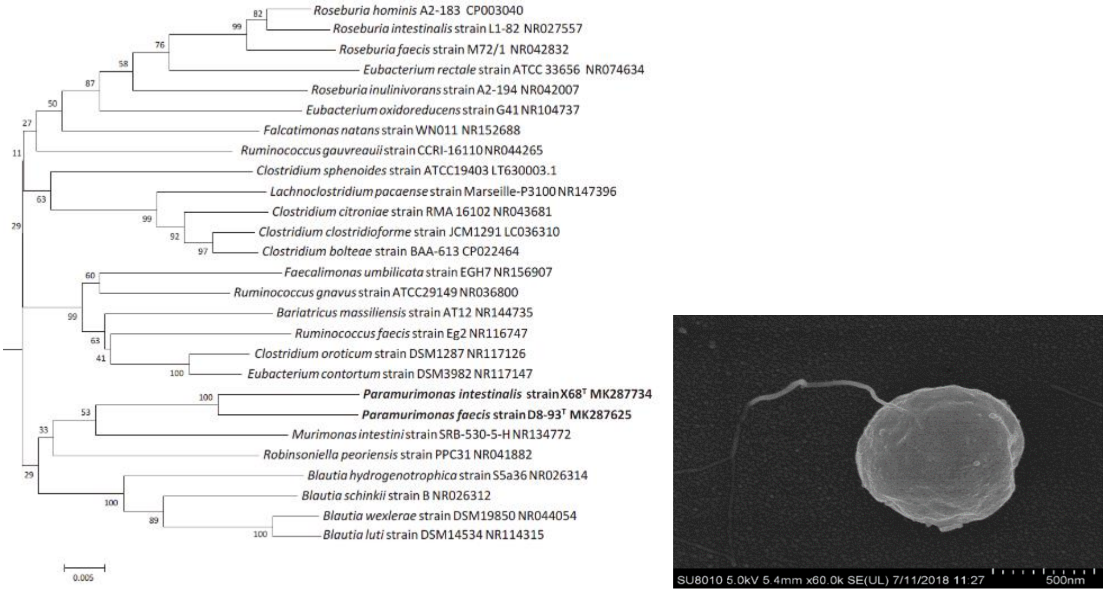


Supplementary Figure 35. The Neighbour-joining phylogenetic tree based on 16S rRNA gene sequences (left, a) and the TEM-based cell morphologic images (right, b) of strain X68$^T$. GenBank accession numbers are given in parentheses. Percentages of bootstrap support are shown at branch nodes. Bootstrap value was 1000. Bar: 0.005 substitutions per nucleotide position.

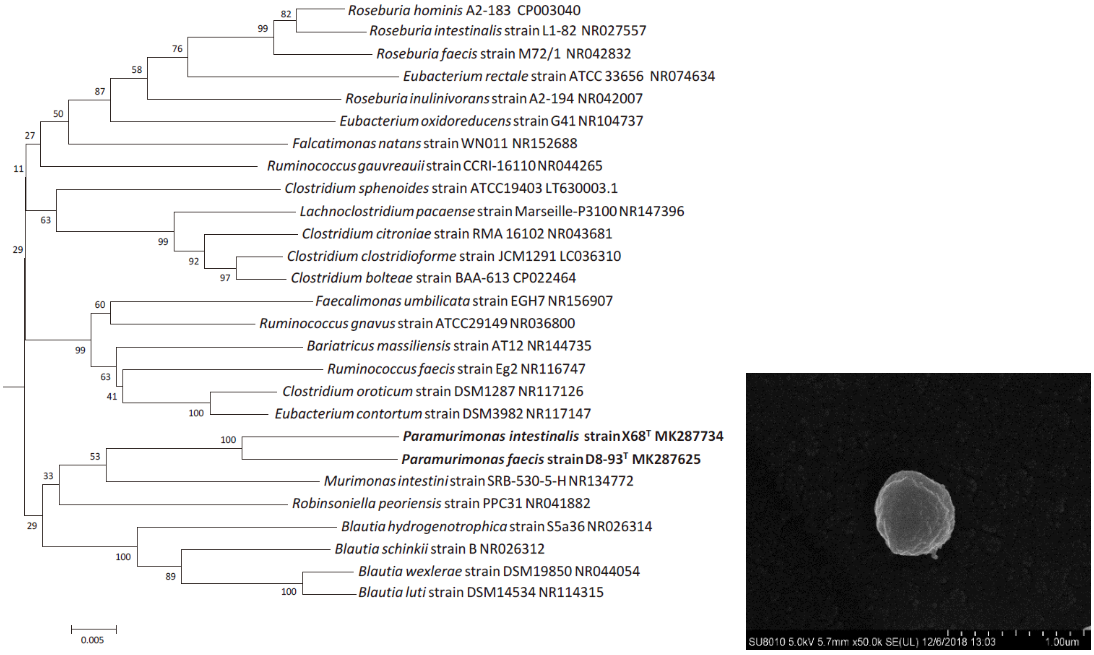


Supplementary Figure 36. The Neighbour-joining phylogenetic tree (left, a) based on 16S rRNA gene sequence and TEM-based cell morphologic images (right, b) of strain D8-93$^T$. GenBank accession numbers are given in parentheses. Percentages of bootstrap support are shown at branch nodes. Bootstrap value is 1000. Bar: 0.005 substitutions per nucleotide position.

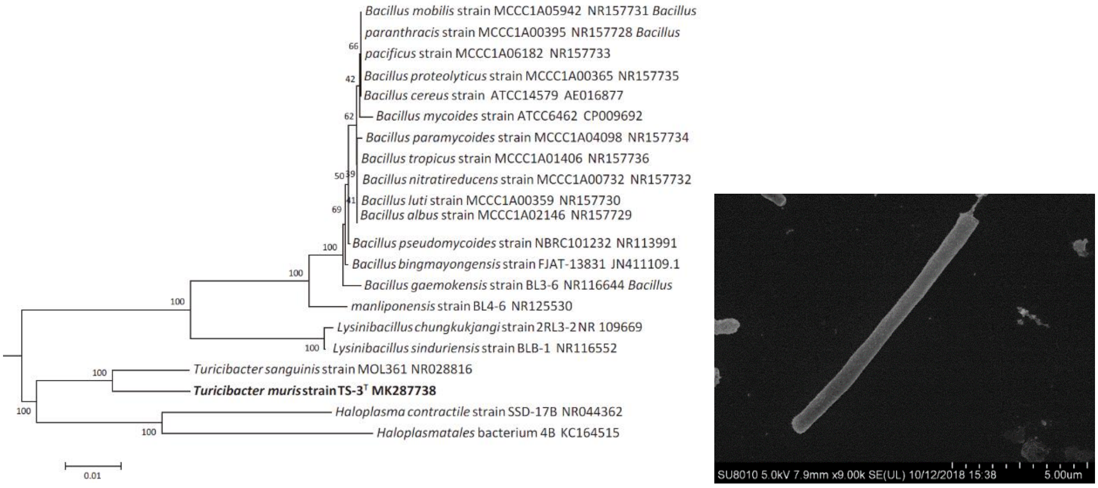


Supplementary Figure 37. The Neighbour-joining phylogenetic tree (left, a) based on 16S rRNA gene sequence and TEM-based cell morphologic images (right, b) of strain TS-3$^T$. GenBank accession numbers are given in parentheses. Percentages of bootstrap support are shown at branch nodes. Bootstrap value is 1000. Bar: 0.01 substitutions per nucleotide position.

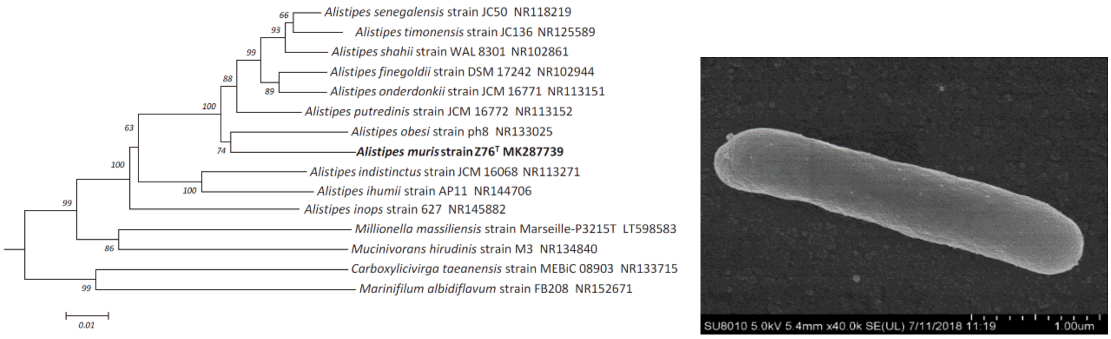


Supplementary Figure 38. The Neighbour-joining phylogenetic tree (left, a) based on 16S rRNA gene sequence and TEM-based cell morphologic images (right, b) of strain Z76$^T$. GenBank accession numbers are given in parentheses. Percentages of bootstrap support are shown at branch nodes. Bootstrap value is 1000. Bar: 0.01 substitutions per nucleotide position.

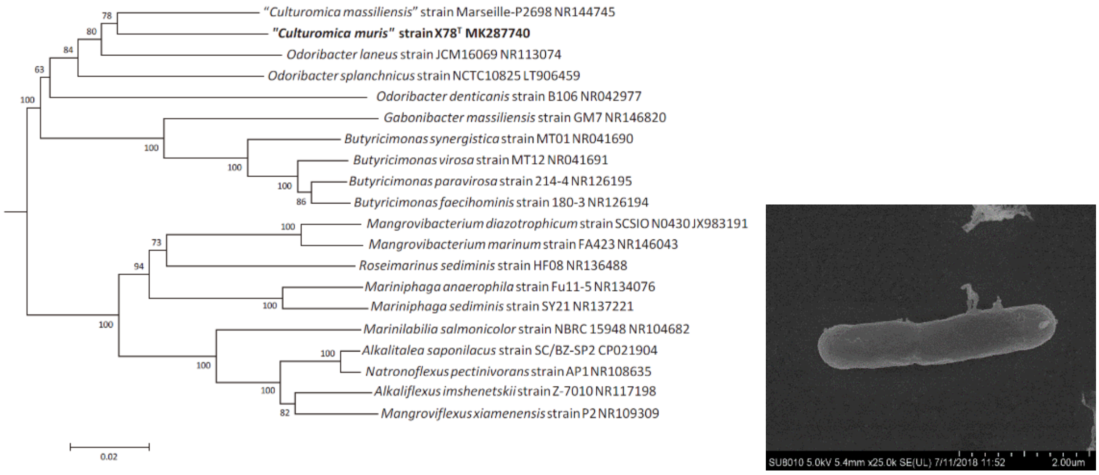


Supplementary Figure 39. The Neighbour-joining phylogenetic tree based on 16S rRNA gene sequences (left, a) and the TEM-based cell morphologic images (right, b) of strain X78$^T$. GenBank

accession numbers are given in parentheses. Percentages of bootstrap support are shown at branch nodes. Bootstrap value was 1000. Bar: 0.002 substitutions per nucleotide position.

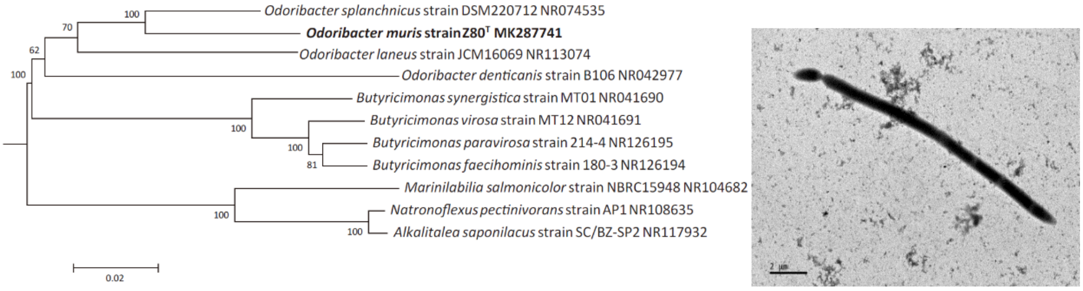


Supplementary Figure 40. The Neighbour-joining phylogenetic tree (left, a) based on 16S rRNA gene sequence and TEM-based cell morphologic images (right, b) of strain Z80$^T$. GenBank accession numbers are given in parentheses. Percentages of bootstrap support are shown at branch nodes. Bootstrap value is 1000. Bar: 0.002 substitutions per nucleotide position.

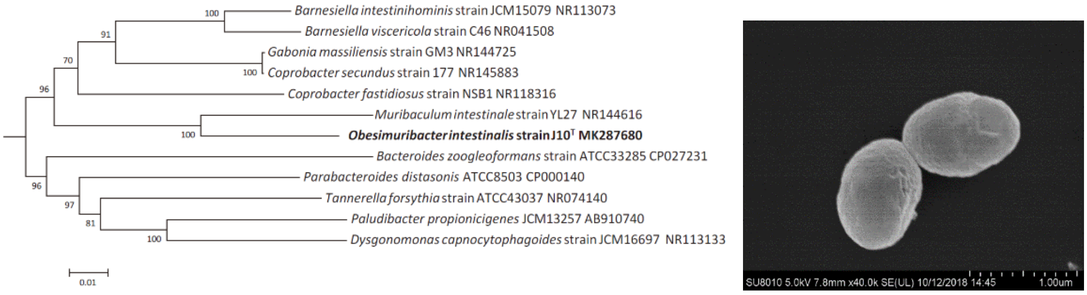


Supplementary Figure 41. The Neighbour-joining phylogenetic tree based on 16S rRNA gene sequences (left, a) and the TEM-based cell morphologic images (right, b) of strain J10$^T$. GenBank accession numbers are given in parentheses. Percentages of bootstrap support are shown at branch nodes. Bootstrap value was 1000. Bar: 0.01 substitutions per nucleotide position.

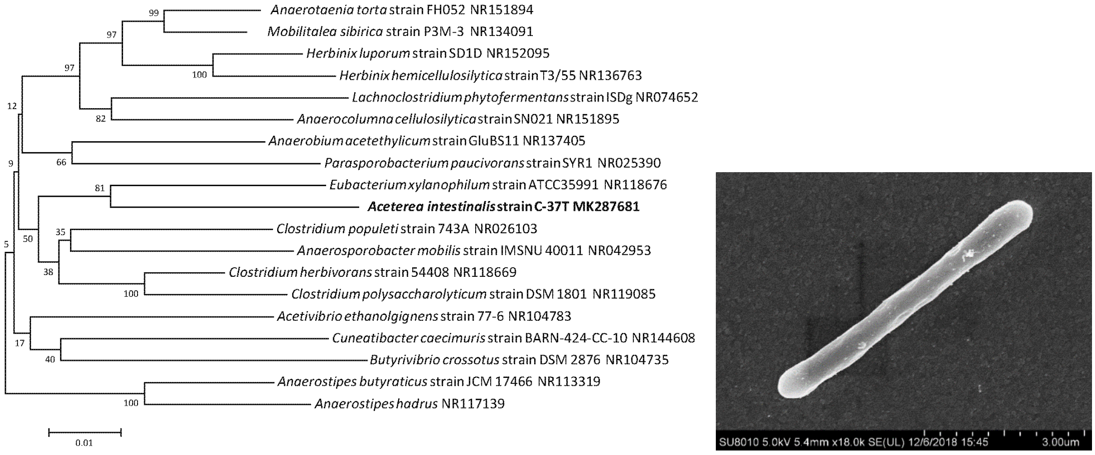


Supplementary Figure 42. The Neighbour-joining phylogenetic tree based on 16S rRNA gene sequences (left, a) and the TEM-based cell morphologic images (right, b) of strain C-37$^T$. GenBank accession numbers are given in parentheses. Percentages of bootstrap support are shown at branch nodes. Bootstrap value was 1000. Bar: 0.01 substitutions per nucleotide position.

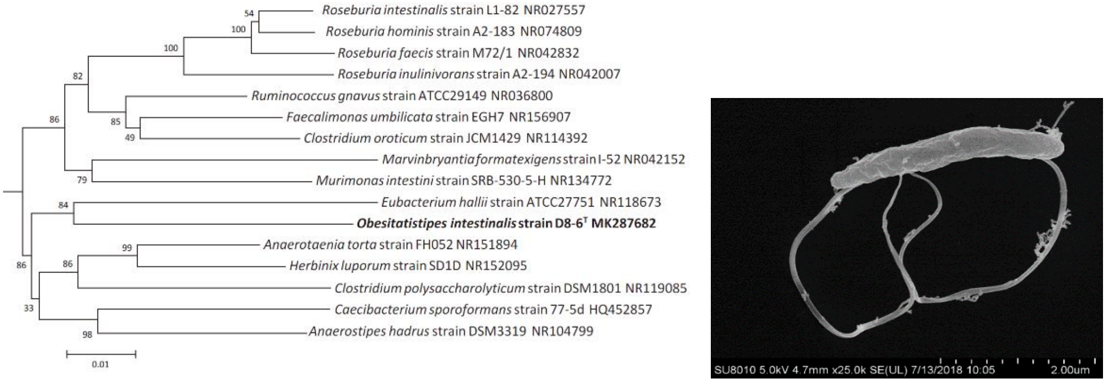


Supplementary Figure 43. The Neighbour-joining phylogenetic tree based on 16S rRNA gene sequences (left, a) and the TEM-based cell morphologic images (right, b) of strain D8-6$^{T}$. GenBank accession numbers are given in parentheses. Percentages of bootstrap support are shown at branch nodes. Bootstrap value was 1000. Bar: 0.01 substitutions per nucleotide position.

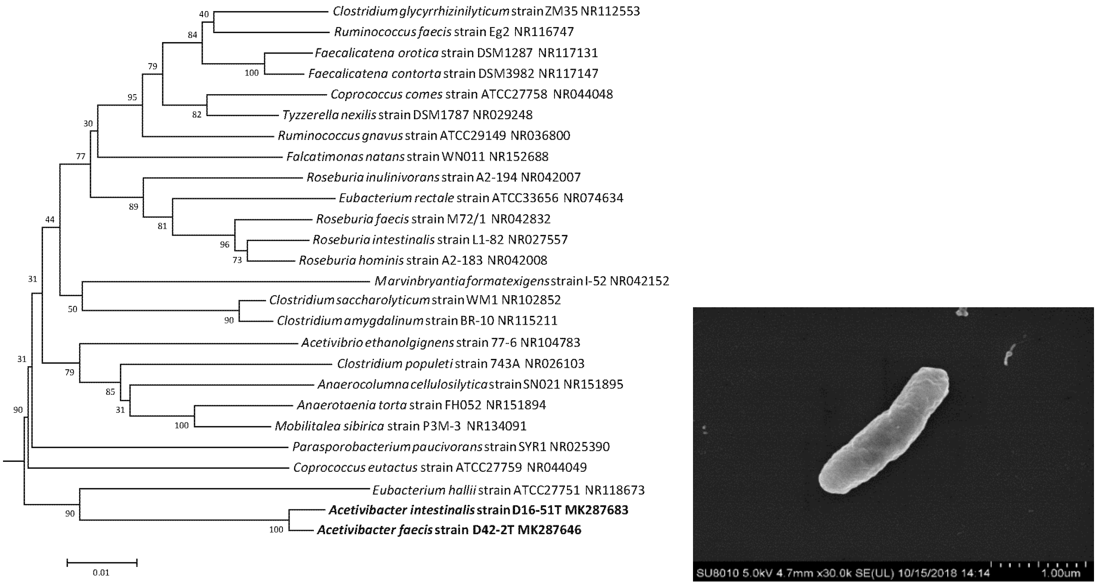


Supplementary Figure 44. The Neighbour-joining phylogenetic tree based on 16S rRNA gene sequences (left, a) and the TEM-based cell morphologic images (right, b) of strain D16-51$^{T}$. GenBank accession numbers are given in parentheses. Percentages of bootstrap support are shown at branch nodes. Bootstrap value was 1000. Bar: 0.01 substitutions per nucleotide position.

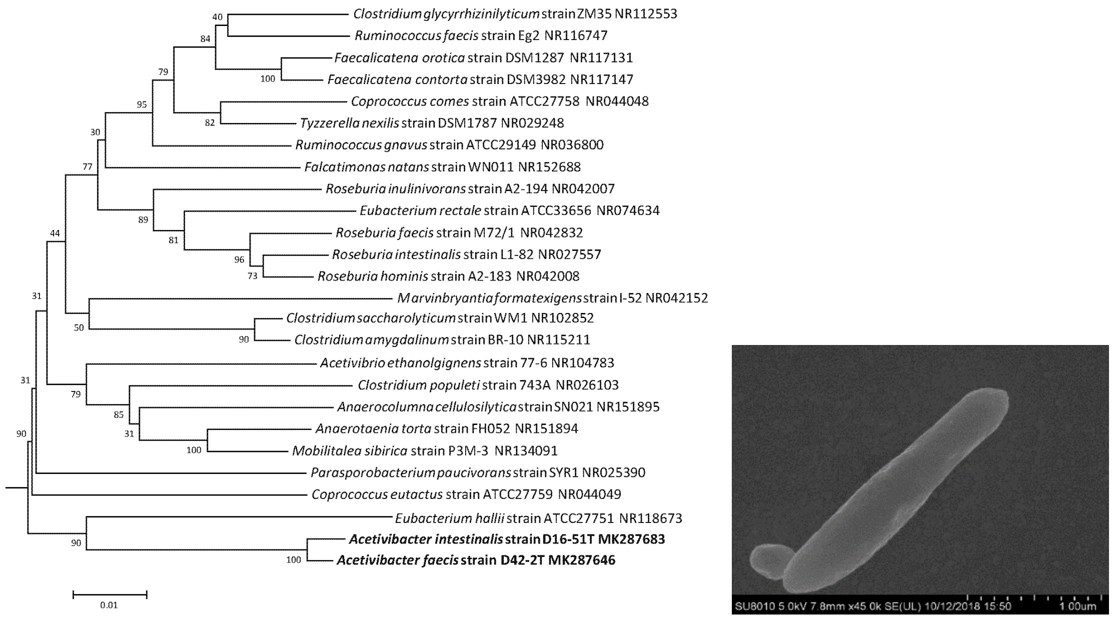


Supplementary Figure 45. The Neighbour-joining phylogenetic tree (left, a) based on 16S rRNA gene sequence and TEM-based cell morphologic images (right, b) of strain C-56$^{T}$. GenBank accession numbers are given in parentheses. Percentages of bootstrap support are shown at branch nodes. Bootstrap value is 1000. Bar: 0.01 substitutions per nucleotide position.

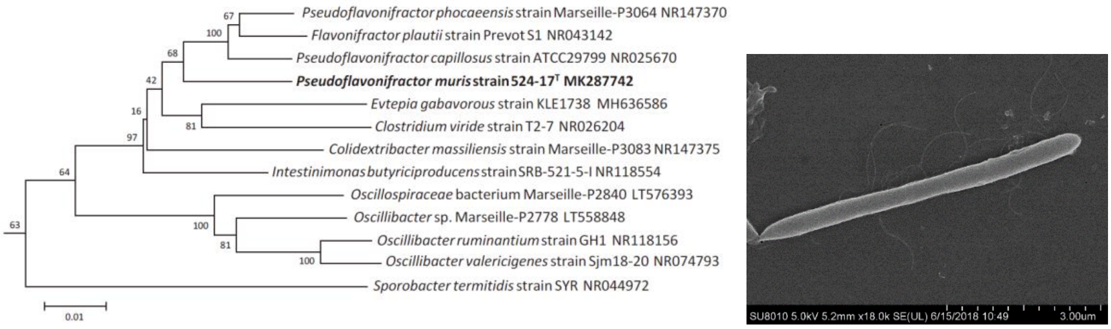


Supplementary Figure 46. The Neighbour-joining phylogenetic tree based on 16S rRNA gene sequences (left, a) and the TEM-based cell morphologic images (right, b) of strain 524-17$^{T}$. GenBank accession numbers are given in parentheses. Percentages of bootstrap support are shown at branch nodes. Bootstrap value was 1000. Bar: 0.01 substitutions per nucleotide position.

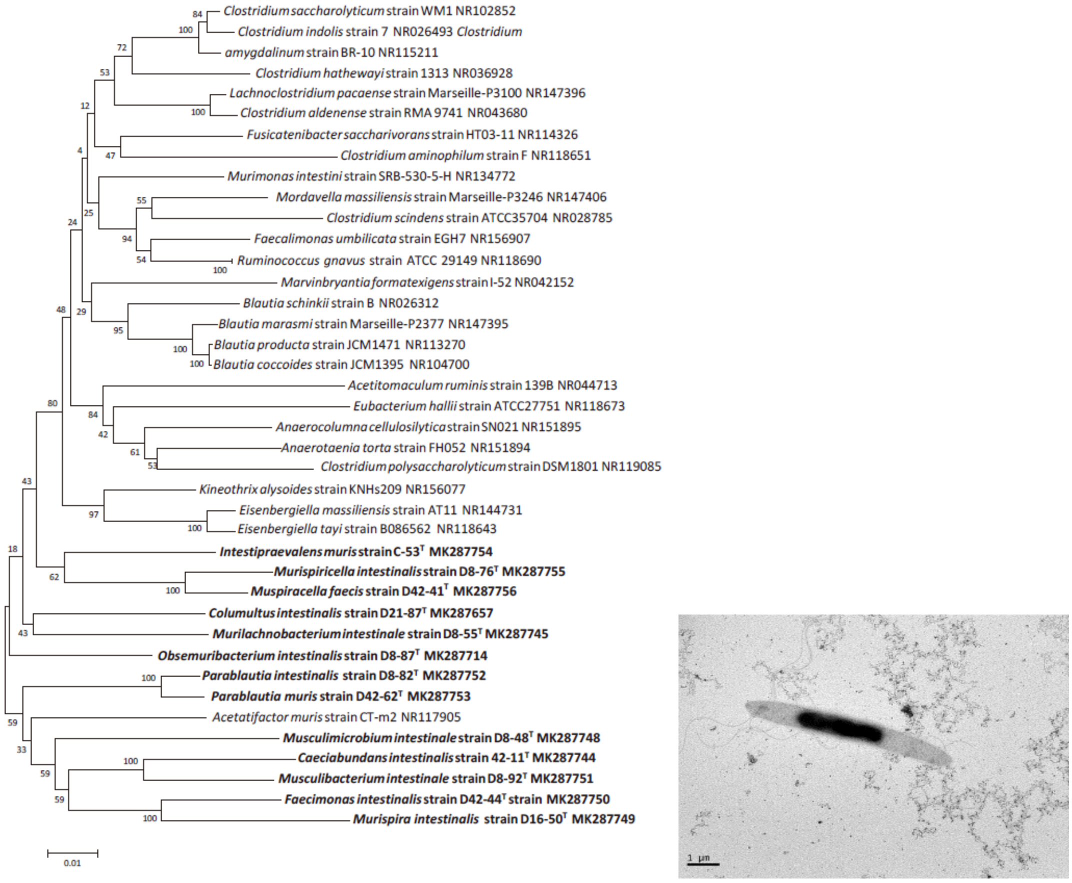


Supplementary Figure 47. The Neighbour-joining phylogenetic tree based on 16S rRNA gene sequences (left, a) and the TEM-based cell morphologic images (right, b) of strain 42-11$^{T}$. GenBank accession numbers are given in parentheses. Percentages of bootstrap support are shown at branch nodes. Bootstrap value was 1000. Bar: 0.01 substitutions per nucleotide position.

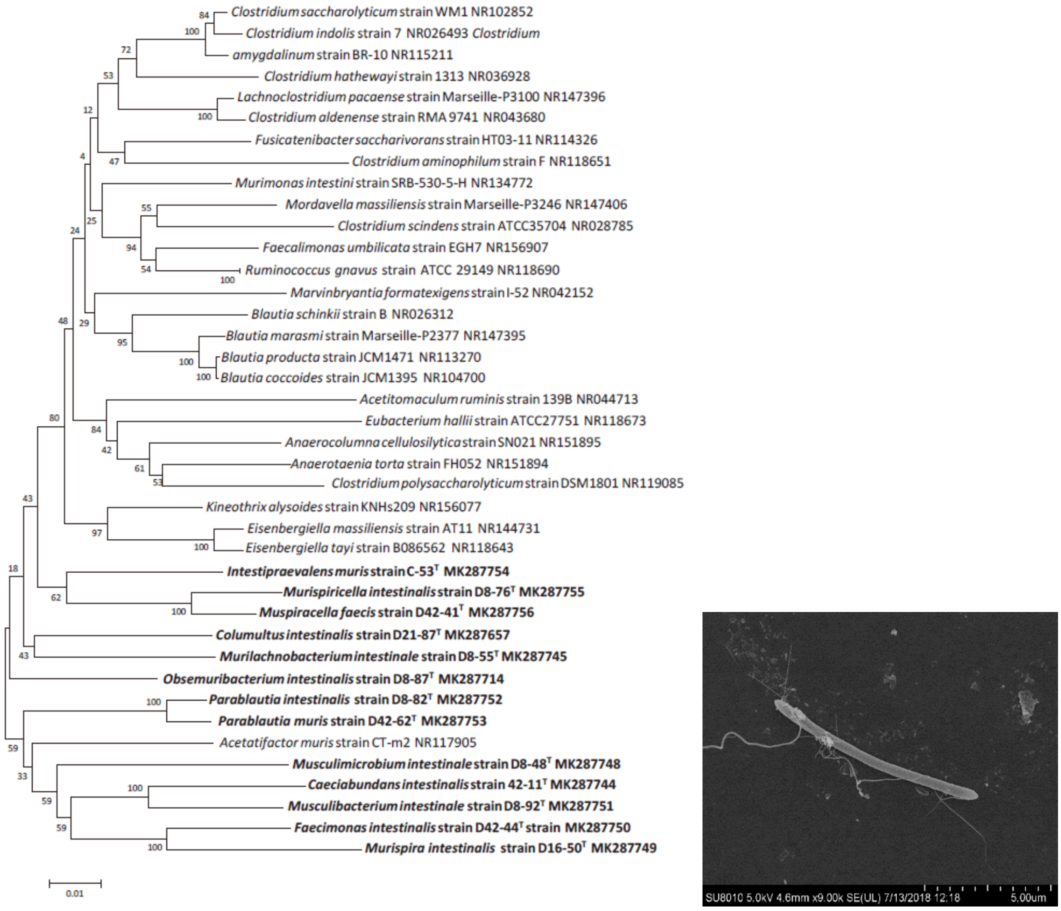


Supplementary Figure 48. The Neighbour-joining phylogenetic tree based on 16S rRNA gene sequences (left, a) and the TEM-based cell morphologic images (right, b) of strain D8-55$^{T}$. GenBank accession numbers are given in parentheses. Percentages of bootstrap support are shown at branch nodes. Bootstrap value was 1000. Bar: 0.01 substitutions per nucleotide position.

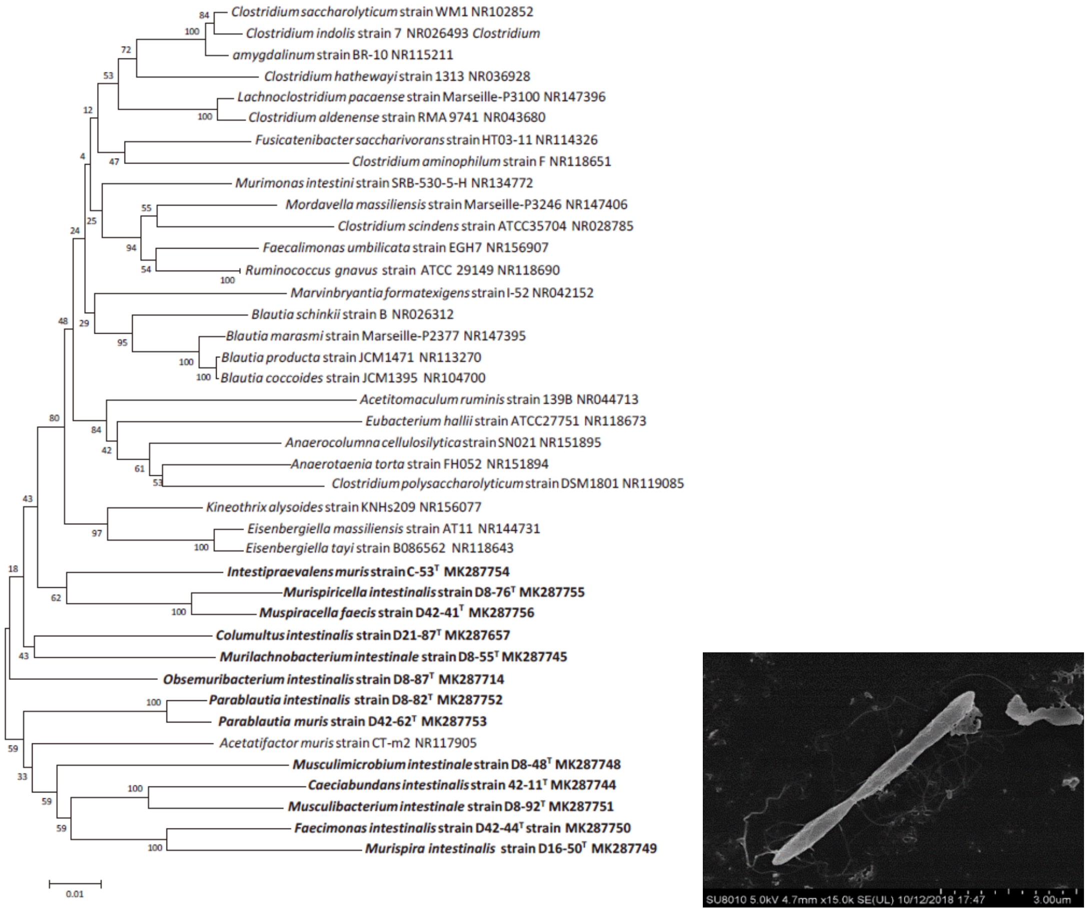


Supplementary Figure 49. The Neighbour-joining phylogenetic tree based on 16S rRNA gene sequences (left, a) and the TEM-based cell morphologic images (right, b) of strain D8-48$^T$. GenBank accession numbers are given in parentheses. Percentages of bootstrap support are shown at branch nodes. Bootstrap value was 1000. Bar: 0.01 substitutions per nucleotide position.

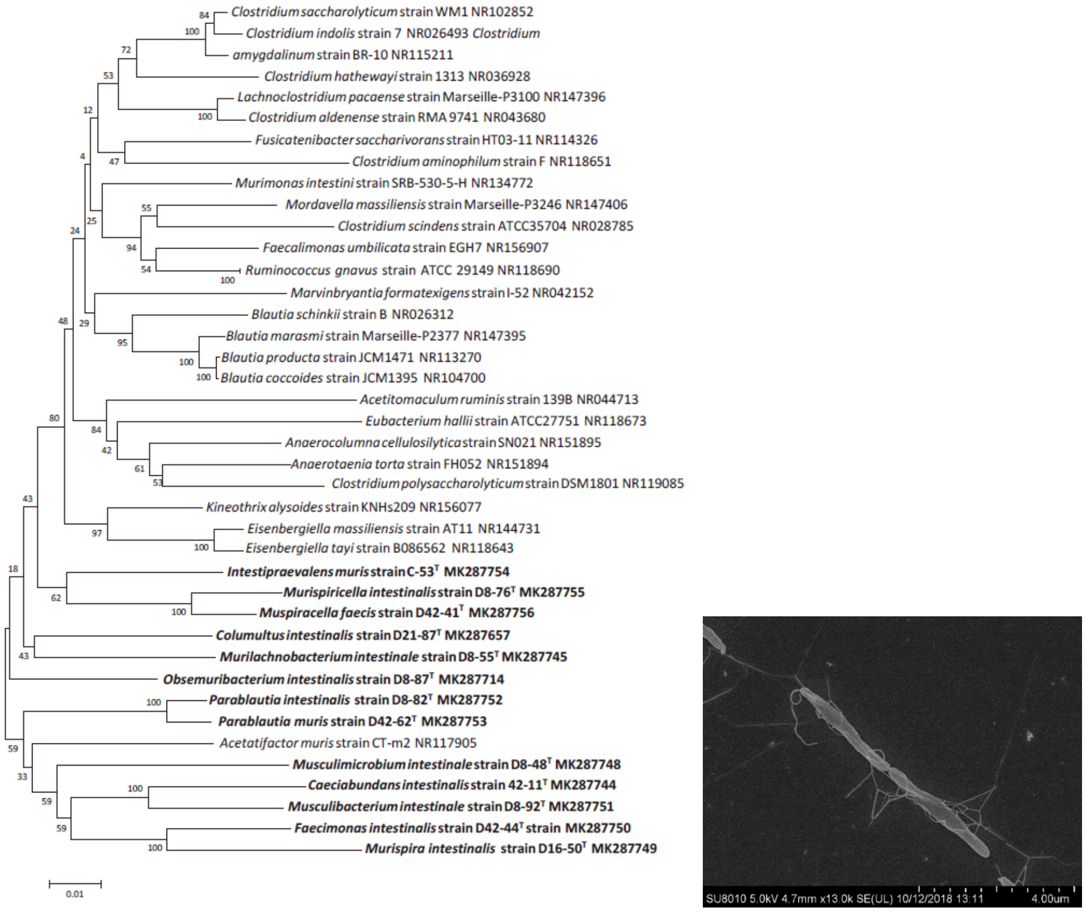


Supplementary Figure 50. The Neighbour-joining phylogenetic tree based on 16S rRNA gene sequences (left, a) and the TEM-based cell morphologic images (right, b) of strain D16-50$^{T}$. GenBank accession numbers are given in parentheses. Percentages of bootstrap support are shown at branch nodes. Bootstrap value was 1000. Bar: 0.01 substitutions per nucleotide position.

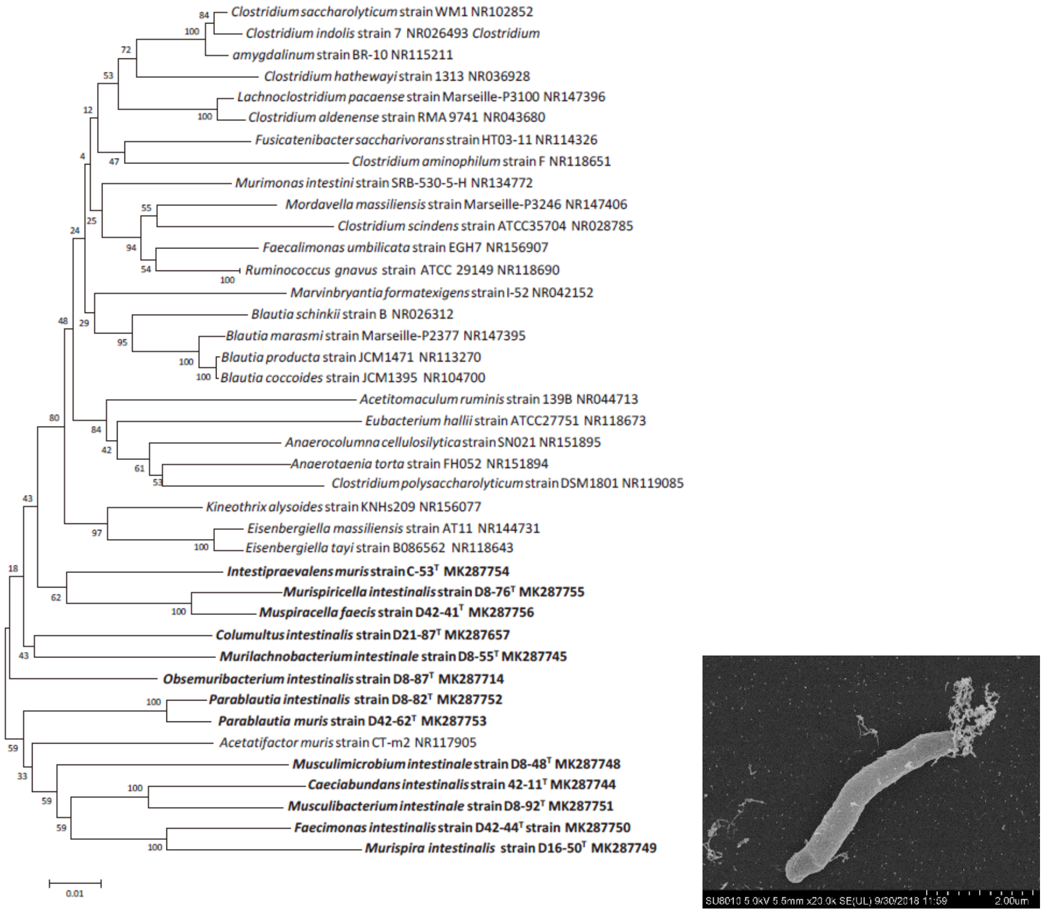


Supplementary Figure 51. The Neighbour-joining phylogenetic tree based on 16S rRNA gene sequences (left, a) and the TEM-based cell morphologic images (right, b) of strain D42-44$^T$. GenBank accession numbers are given in parentheses. Percentages of bootstrap support are shown at branch nodes. Bootstrap value was 1000. Bar: 0.01 substitutions per nucleotide position.

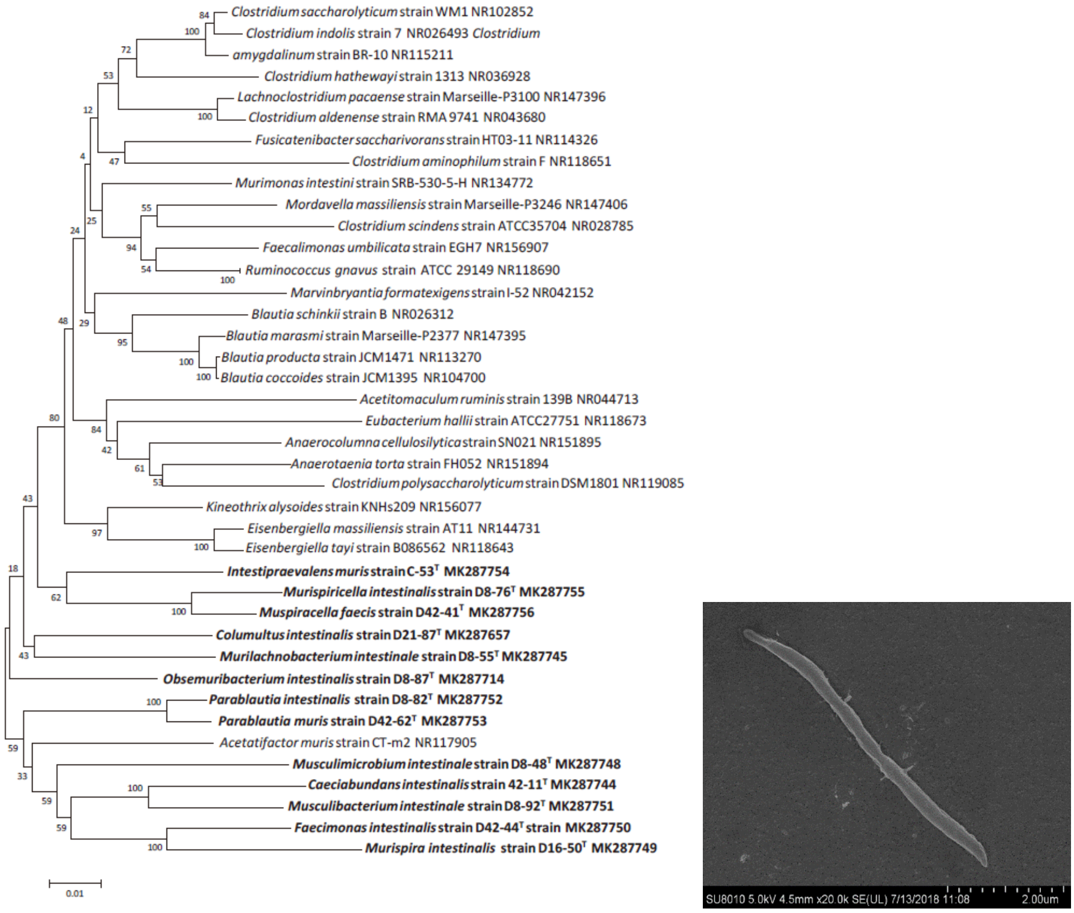


Supplementary Figure 52. The Neighbour-joining phylogenetic tree based on 16S rRNA gene sequences (left, a) and the TEM-based cell morphologic images (right, b). of strain D8-92$^{T}$. GenBank accession numbers are given in parentheses. Percentages of bootstrap support are shown at branch nodes. Bootstrap value was 1000. Bar: 0.01 substitutions per nucleotide position.

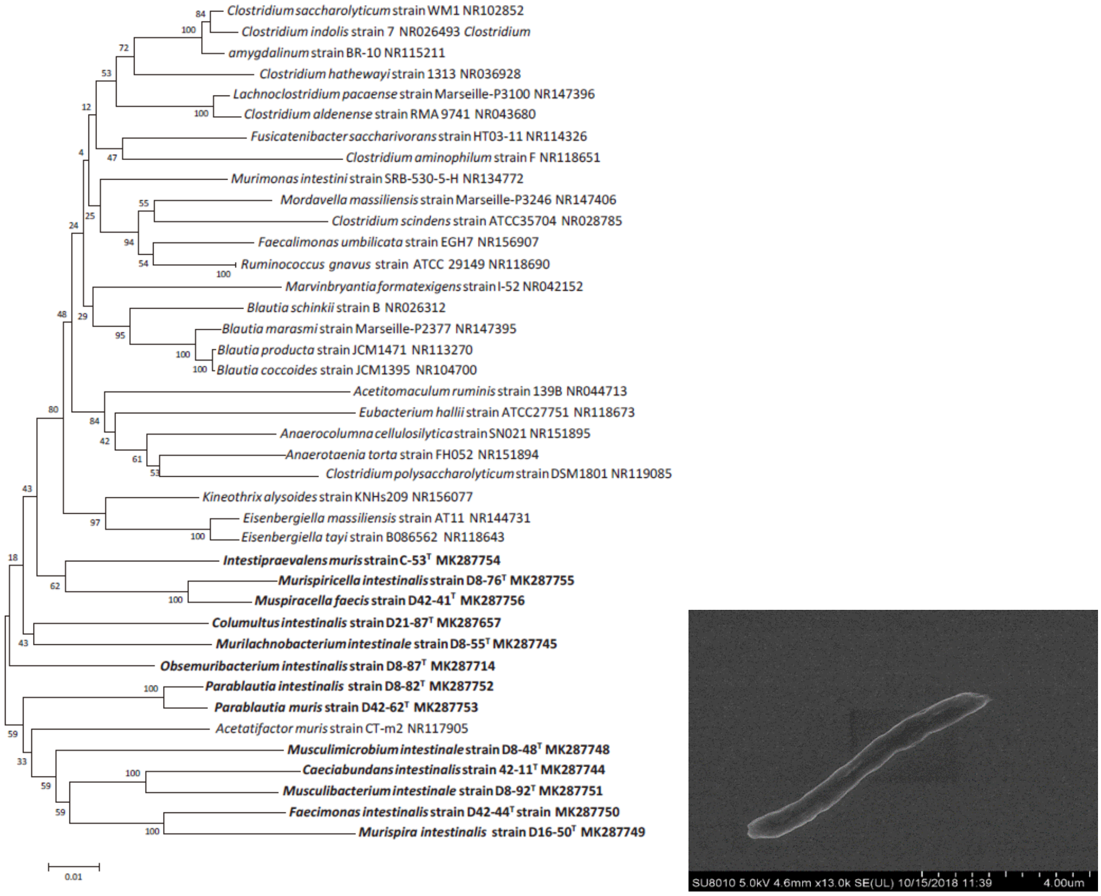


Supplementary Figure 53. The Neighbour-joining phylogenetic tree based on 16S rRNA gene sequences (left, a) and the TEM-based cell morphologic images (right, b) of strain D8-82$^{T}$. GenBank accession numbers are given in parentheses. Percentages of bootstrap support are shown at branch nodes. Bootstrap value was 1000. Bar: 0.01 substitutions per nucleotide position.

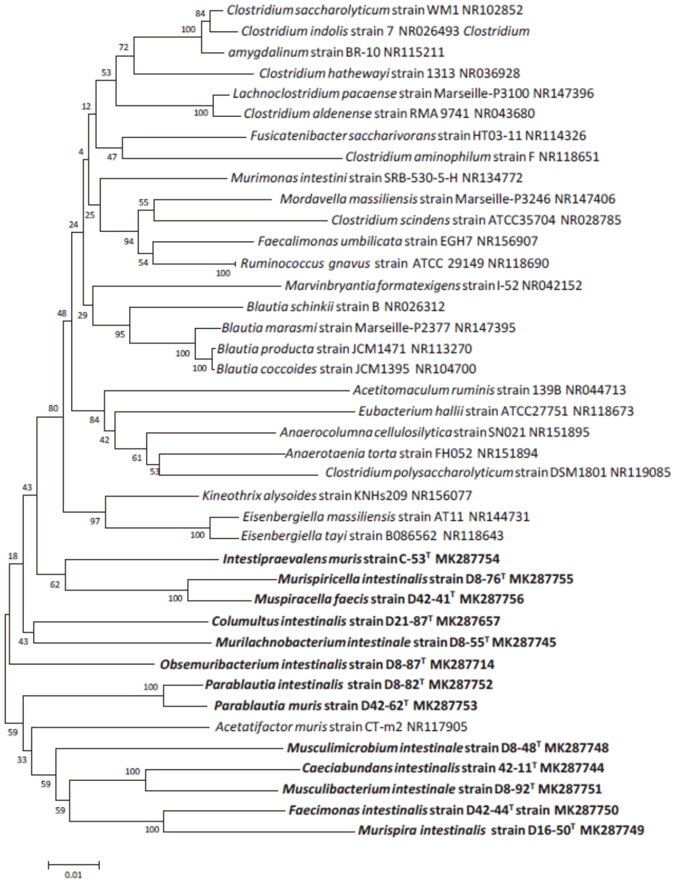

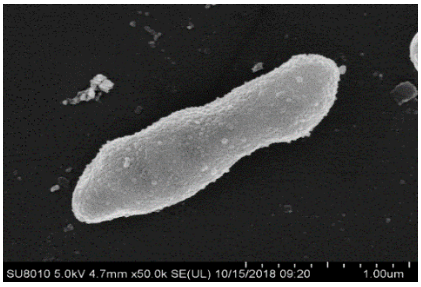


Supplementary Figure 54. The Neighbour-joining phylogenetic tree (left, a) based on 16S rRNA gene sequence and TEM-based cell morphologic images (right, b) of strain D42-62$^{T}$. GenBank accession numbers are given in parentheses. Percentages of bootstrap support are shown at branch nodes. Bootstrap value is 1000. Bar: 0.01 substitutions per nucleotide position.

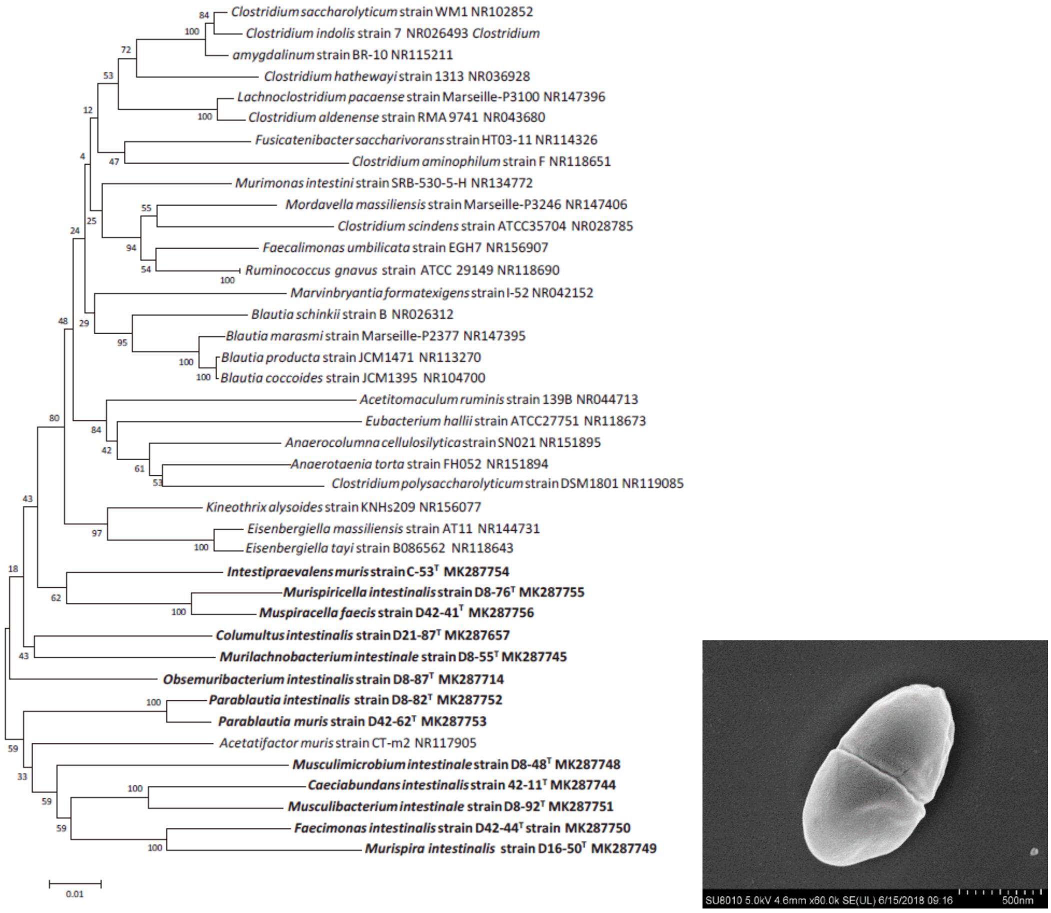


Supplementary Figure 55. The Neighbour-joining phylogenetic tree based on 16S rRNA gene sequences (left, a) and the TEM-based cell morphologic images (right, b) of strain C-53$^{T}$. GenBank accession numbers are given in parentheses. Percentages of bootstrap support are shown at branch nodes. Bootstrap value was 1000. Bar: 0.01 substitutions per nucleotide position.

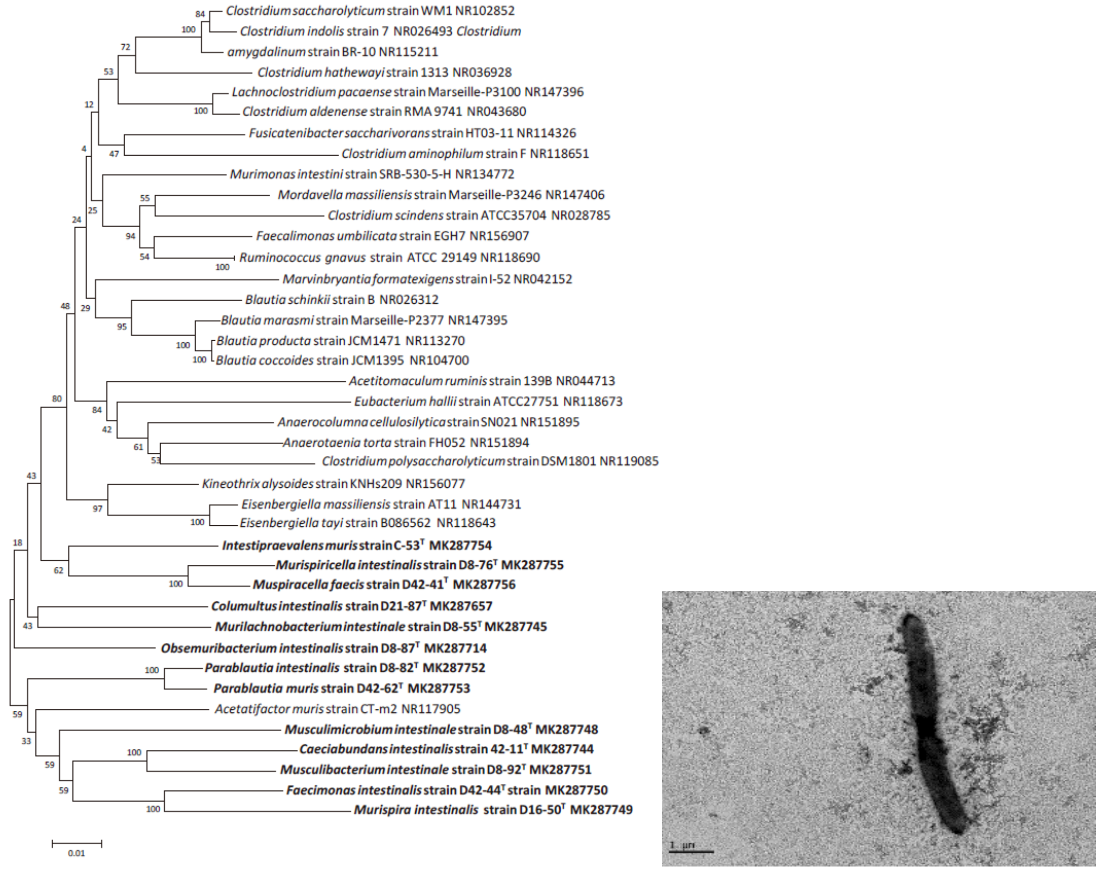


Supplementary Figure 56. The Neighbour-joining phylogenetic tree based on 16S rRNA gene sequences (left, a) and the TEM-based cell morphologic images (right, b) of strain D8-76$^{T}$. GenBank accession numbers are given in parentheses. Percentages of bootstrap support are shown at branch nodes. Bootstrap value was 1000. Bar: 0.01 substitutions per nucleotide position.

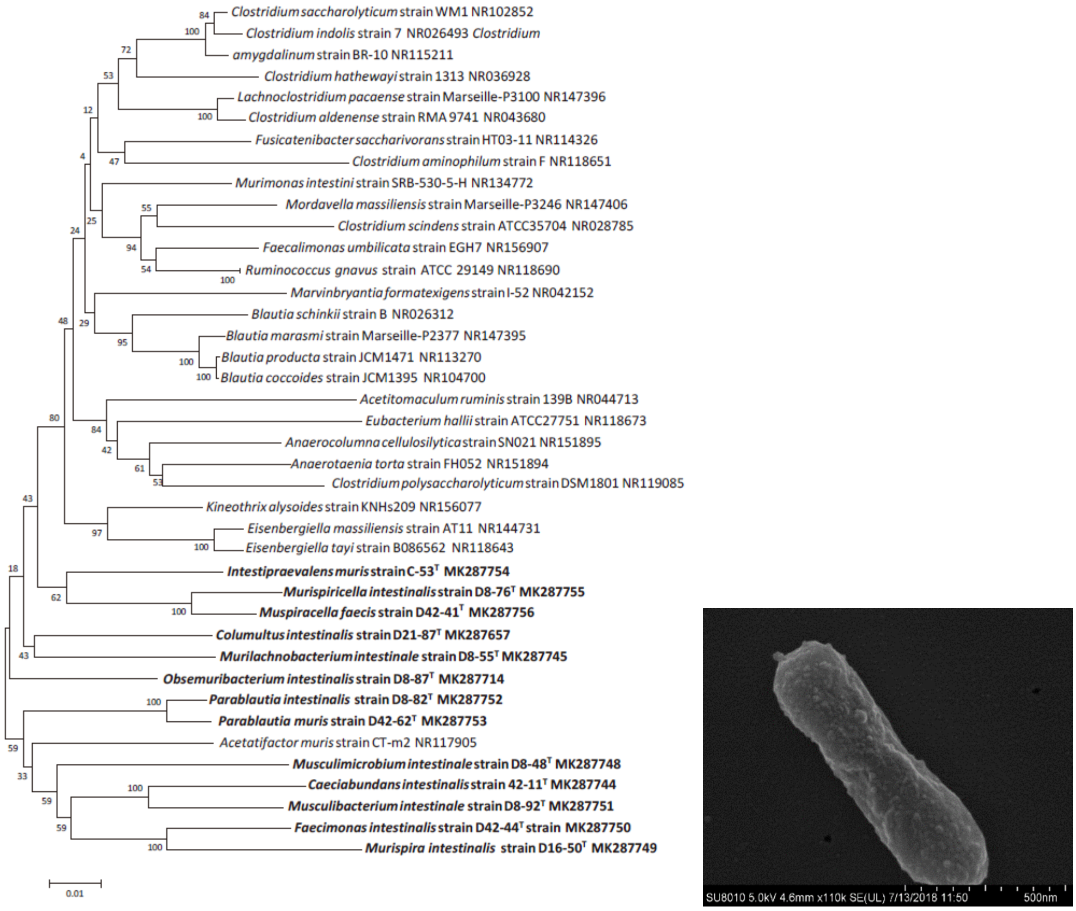


Supplementary Figure 57. The Neighbour-joining phylogenetic tree (left, a) based on 16S rRNA gene sequence and TEM-based cell morphologic images (right, b) of strain D42-41$^{T}$. GenBank accession numbers are given in parentheses. Percentages of bootstrap support are shown at branch nodes. Bootstrap value is 1000. Bar: 0.01 substitutions per nucleotide position.

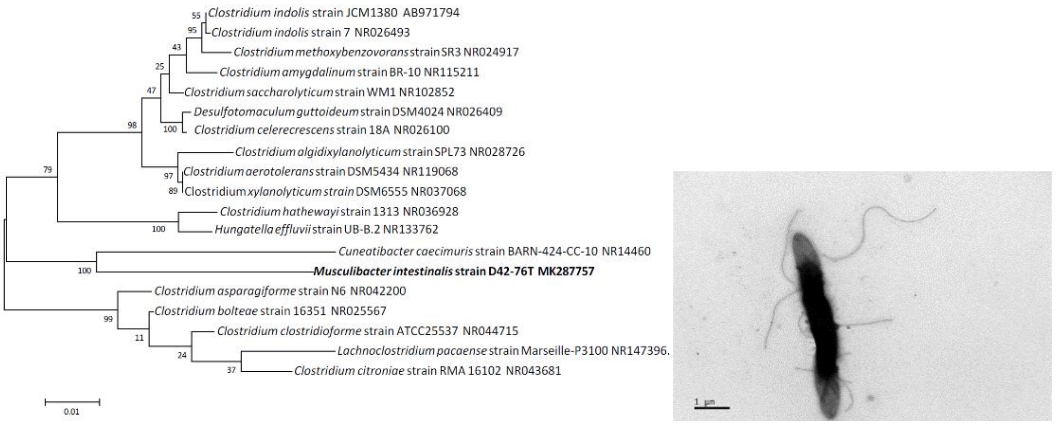


Supplementary Figure 58. The Neighbour-joining phylogenetic tree based on 16S rRNA gene sequences (left, a) and the TEM-based cell morphologic images (right, b) of strain D42-76$^{T}$. GenBank accession numbers are given in parentheses. Percentages of bootstrap support are shown at branch nodes. Bootstrap value was 1000. Bar: 0.01 substitutions per nucleotide position.

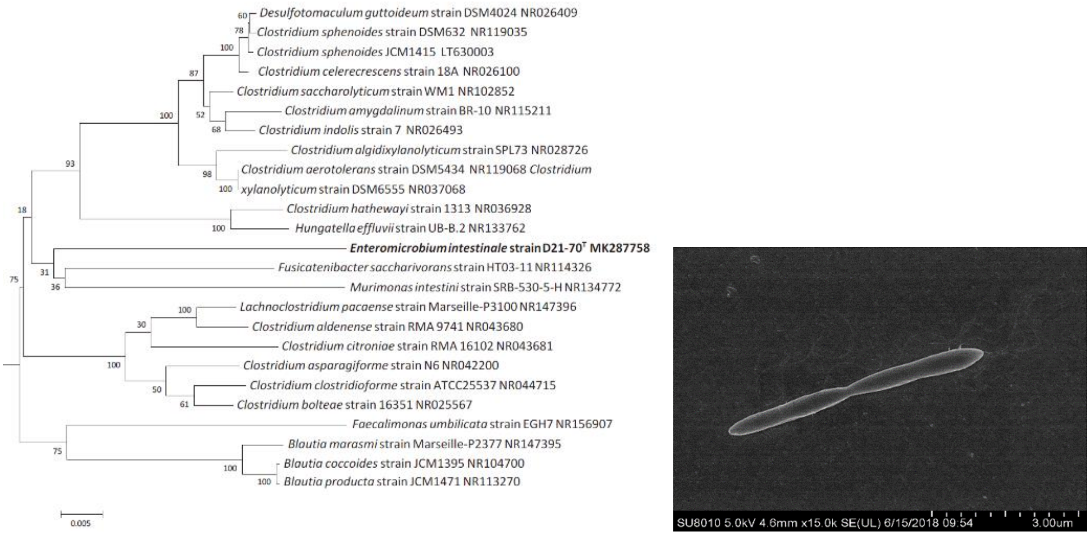


Supplementary Figure 59. The Neighbour-joining phylogenetic tree based on 16S rRNA gene sequences (left, a) and the TEM-based cell morphologic images (right, b) of strain D21-70$^T$. GenBank accession numbers are given in parentheses. Percentages of bootstrap support are shown at branch nodes. Bootstrap value was 1000. Bar: 0.01 substitutions per nucleotide position.

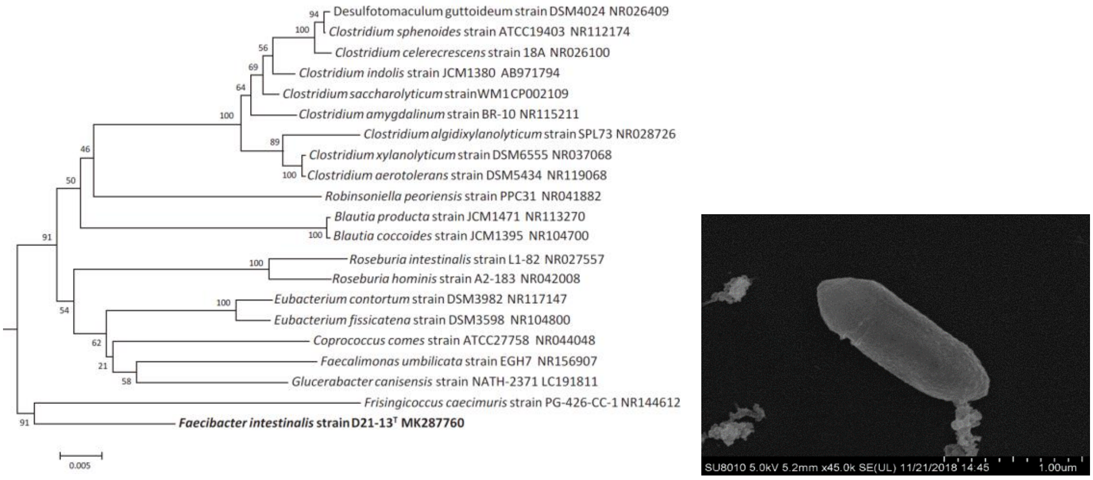


Supplementary Figure 60. The Neighbour-joining phylogenetic tree based on 16S rRNA gene sequences (left, a) and the TEM-based cell morphologic images (right, b) of strain D21-13$^T$. GenBank accession numbers are given in parentheses. Percentages of bootstrap support are shown at branch nodes. Bootstrap value was 1000. Bar: 0.005 substitutions per nucleotide position.

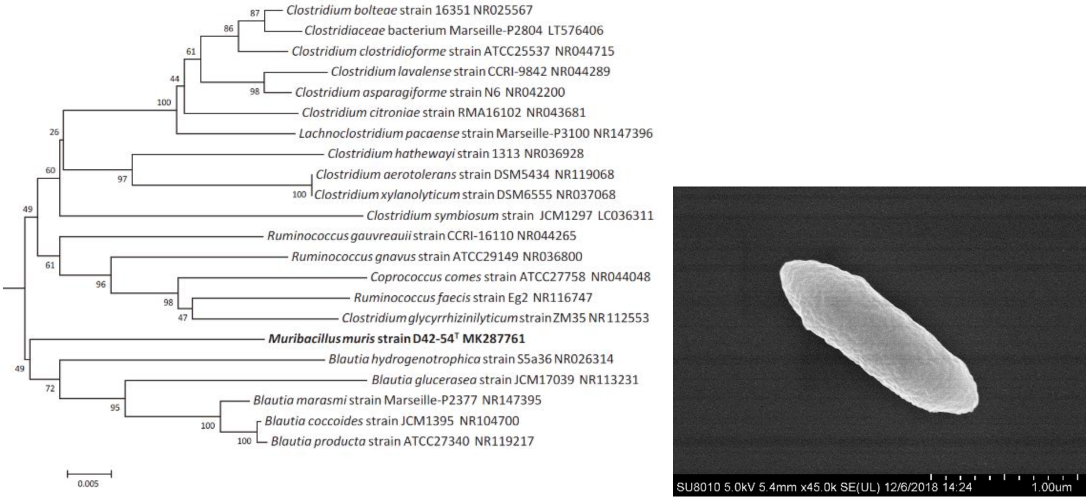


Supplementary Figure 61. The Neighbour-joining phylogenetic tree based on 16S rRNA gene sequences (left, a) and the TEM-based cell morphologic images (right, b) of strain D42-54$^{T}$. GenBank accession numbers are given in parentheses. Percentages of bootstrap support are shown at branch nodes. Bootstrap value was 1000. Bar: 0.005 substitutions per nucleotide position.

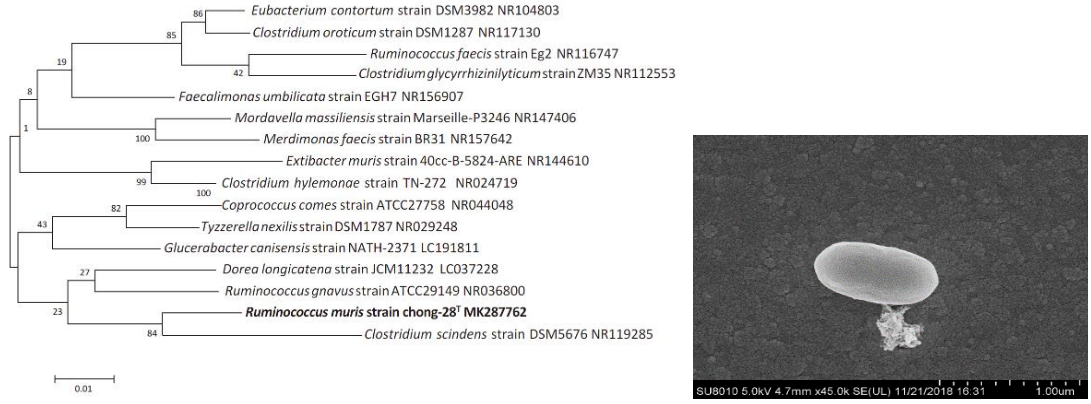


Supplementary Figure 62. The Neighbour-joining phylogenetic tree based on 16S rRNA gene sequences (left, a) and the TEM-based cell morphologic images (right, b) of strain chong-28$^{T}$. GenBank accession numbers are given in parentheses. Percentages of bootstrap support are shown at branch nodes. Bootstrap value was 1000. Bar: 0.005 substitutions per nucleotide position.

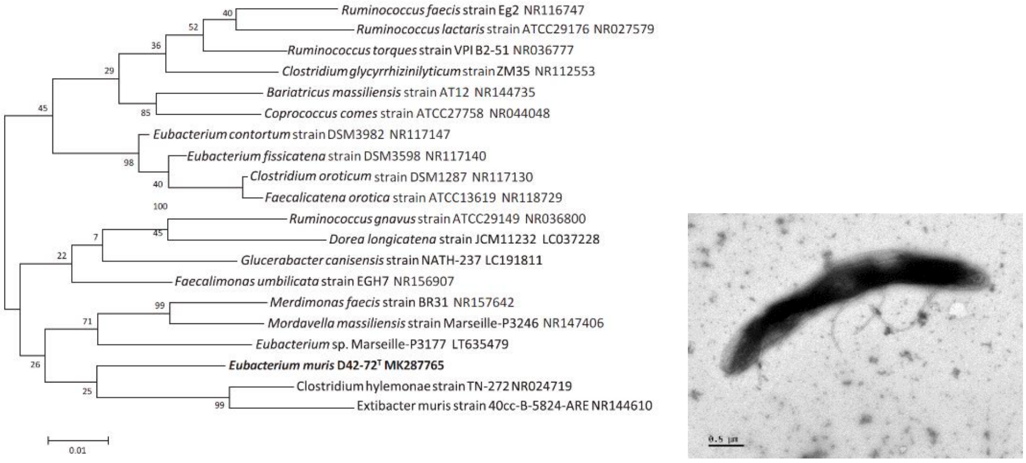


Supplementary Figure 63. The Neighbour-joining phylogenetic tree based on 16S rRNA gene sequences (left, a) and the TEM-based cell morphologic images (right, b) of strain D42-72$^{T}$.

GenBank accession numbers are given in parentheses. Percentages of bootstrap support are shown at branch nodes. Bootstrap value was 1000. Bar: 0.005 substitutions per nucleotide position.

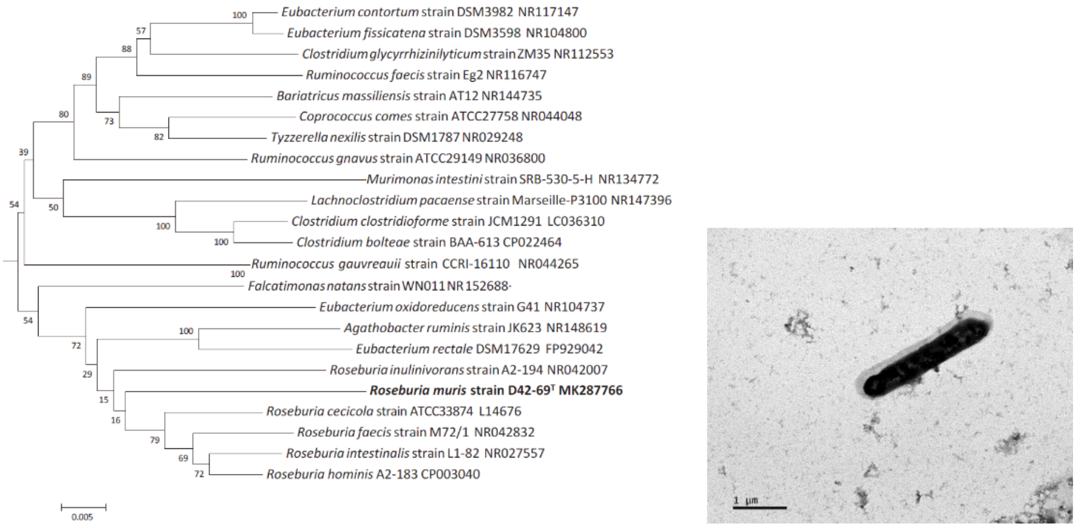


Supplementary Figure 64. The Neighbour-joining phylogenetic tree (left, a) based on 16S rRNA gene sequence and TEM-based cell morphologic images (right, b) of strain D42-69$^{T}$. GenBank accession numbers are given in parentheses. Percentages of bootstrap support are shown at branch nodes. Bootstrap value is 1000. Bar: 0.005 substitutions per nucleotide position.

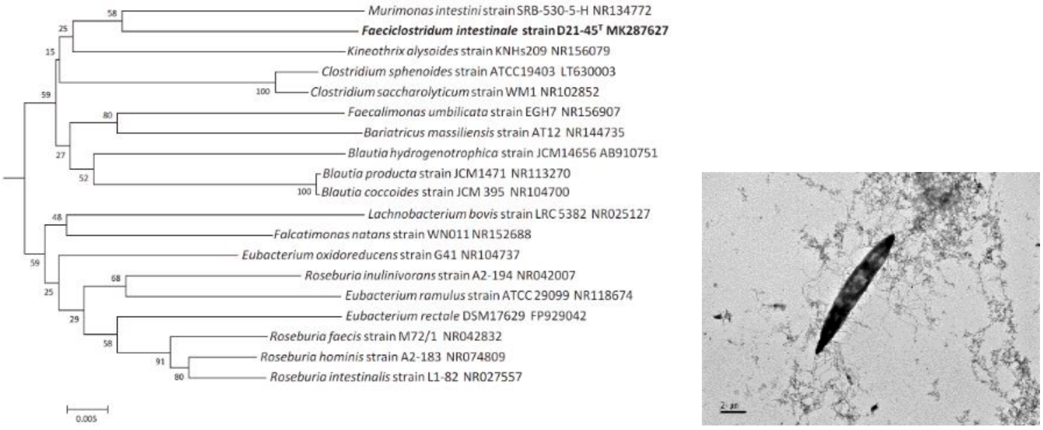


Supplementary Figure 65. The Neighbour-joining phylogenetic tree based on 16S rRNA gene sequences (left, a) and the TEM-based cell morphologic images (right, b) of strain D21-45$^{T}$. GenBank accession numbers are given in parentheses. Percentages of bootstrap support are shown at branch nodes. Bootstrap value was 1000. Bar: 0.005 substitutions per nucleotide position.

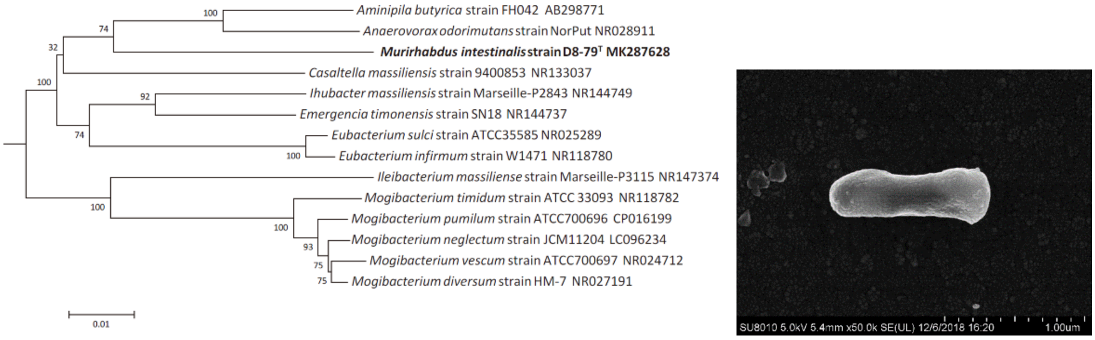

Supplementary Figure 66. The Neighbour-joining phylogenetic tree based on 16S rRNA gene sequences (left, a) and the TEM-based cell morphologic images (right, b) of strain D8-79$^T$. GenBank accession numbers are given in parentheses. Percentages of bootstrap support are shown at branch nodes. Bootstrap value was 1000. Bar: 0.01 substitutions per nucleotide position.

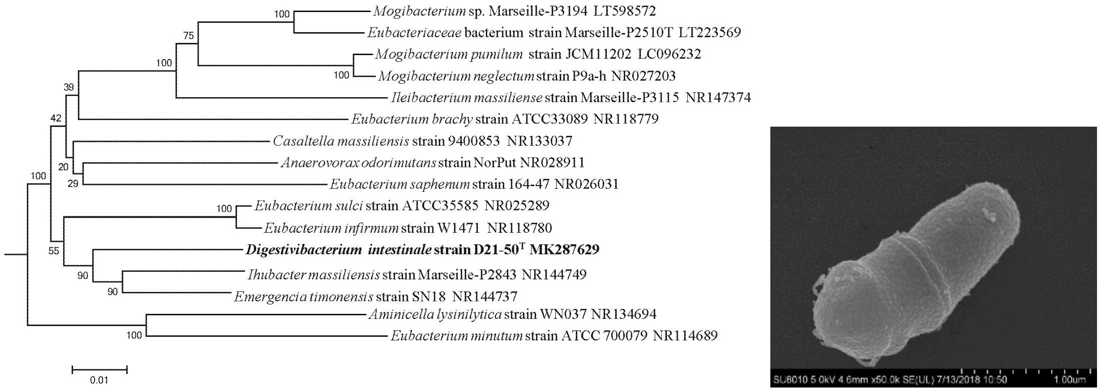


Supplementary Figure 67. The Neighbour-joining phylogenetic tree based on 16S rRNA gene sequences (left, a) and the TEM-based cell morphologic images (right, b) of strain D21-50$^T$. GenBank accession numbers are given in parentheses. Percentages of bootstrap support are shown at branch nodes. Bootstrap value was 1000. Bar: 0.01 substitutions per nucleotide position.

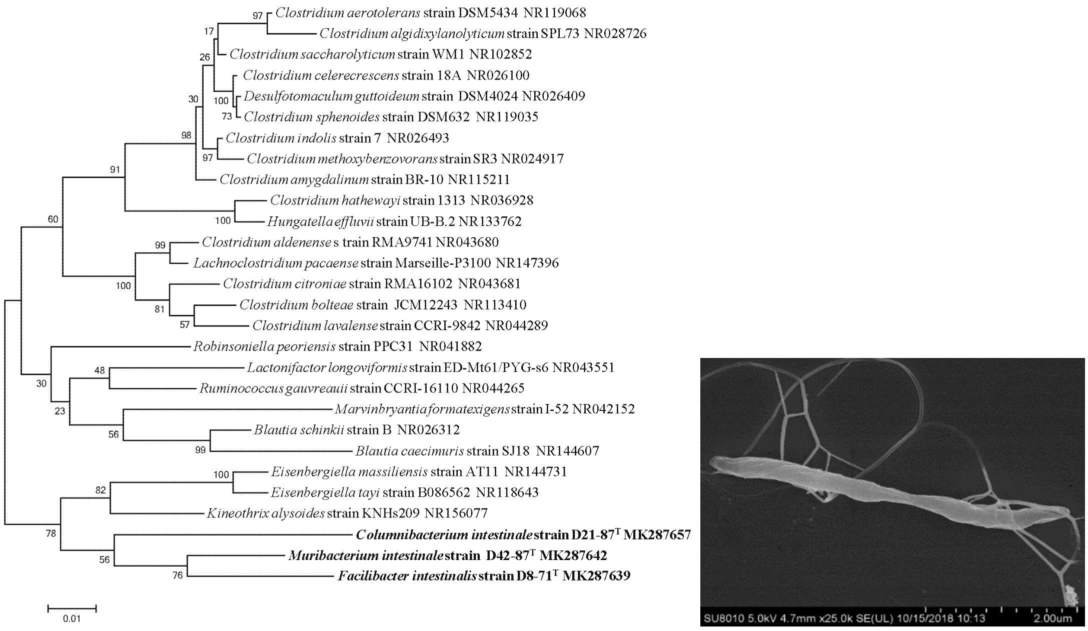


Supplementary Figure 68. The Neighbour-joining phylogenetic tree based on 16S rRNA gene sequences (left, a) and the TEM-based cell morphologic images (right, b) of strain D8-71$^T$. GenBank accession numbers are given in parentheses. Percentages of bootstrap support are shown at branch nodes. Bootstrap value was 1000. Bar: 0.01 substitutions per nucleotide position.

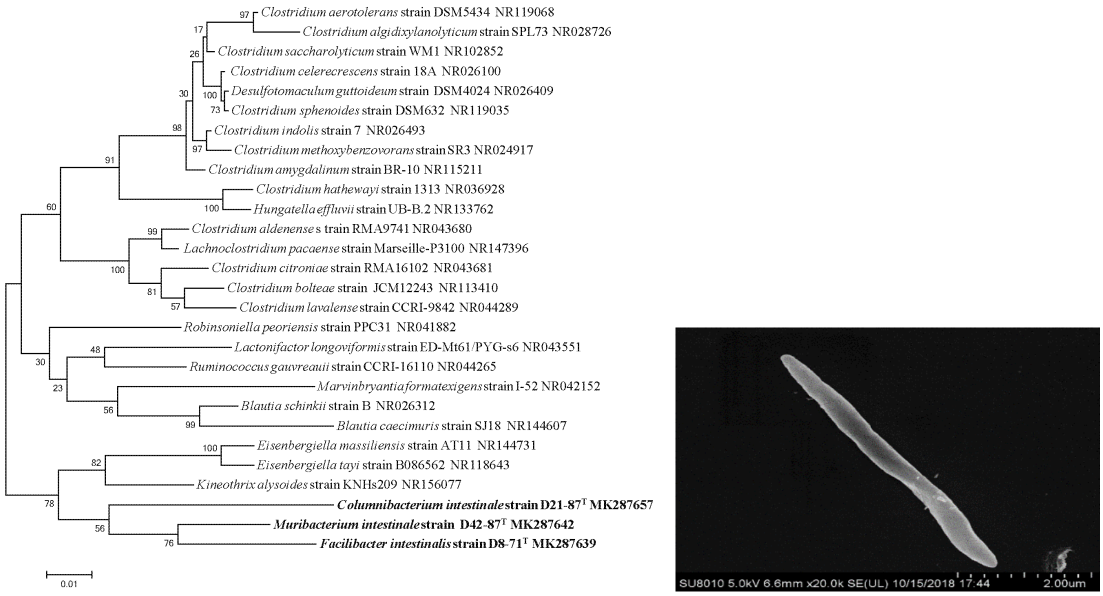


Supplementary Figure 69. The Neighbour-joining phylogenetic tree based on 16S rRNA gene sequences (left, a) and the TEM-based cell morphologic images (right, b) of strain D42-87$^{T}$. GenBank accession numbers are given in parentheses. Percentages of bootstrap support are shown at branch nodes. Bootstrap value was 1000. Bar: 0.005 substitutions per nucleotide position.

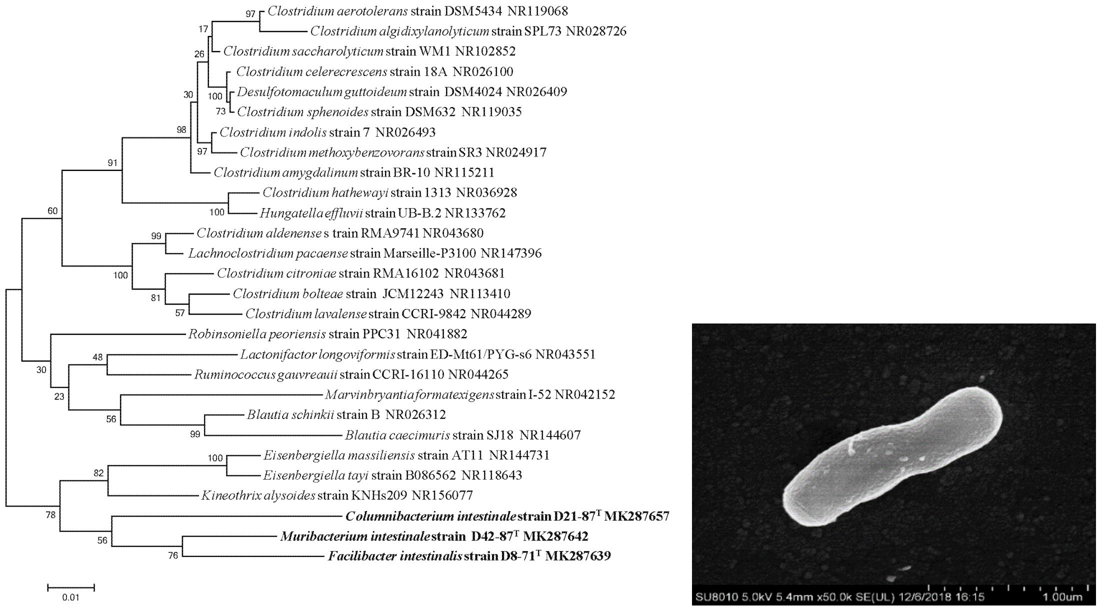


Supplementary Figure 70. The Neighbour-joining phylogenetic tree based on 16S rRNA gene sequences (left, a) and the TEM-based cell morphologic images (right, b) of strain D21-87$^{T}$. GenBank accession numbers are given in parentheses. Percentages of bootstrap support are shown at branch nodes. Bootstrap value was 1000. Bar: 0.005 substitutions per nucleotide position.

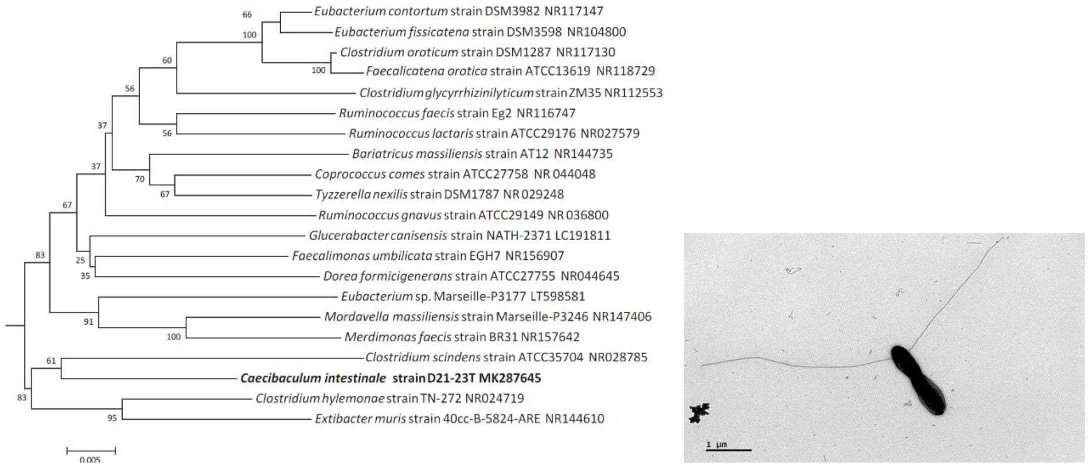


Supplementary Figure 71. The Neighbour-joining phylogenetic tree based on 16S rRNA gene sequences (left, a) and the TEM-based cell morphologic images (right, b) of strain D21-23$^{T}$. GenBank accession numbers are given in parentheses. Percentages of bootstrap support are shown at branch nodes. Bootstrap value was 1000. Bar: 0.005 substitutions per nucleotide position.

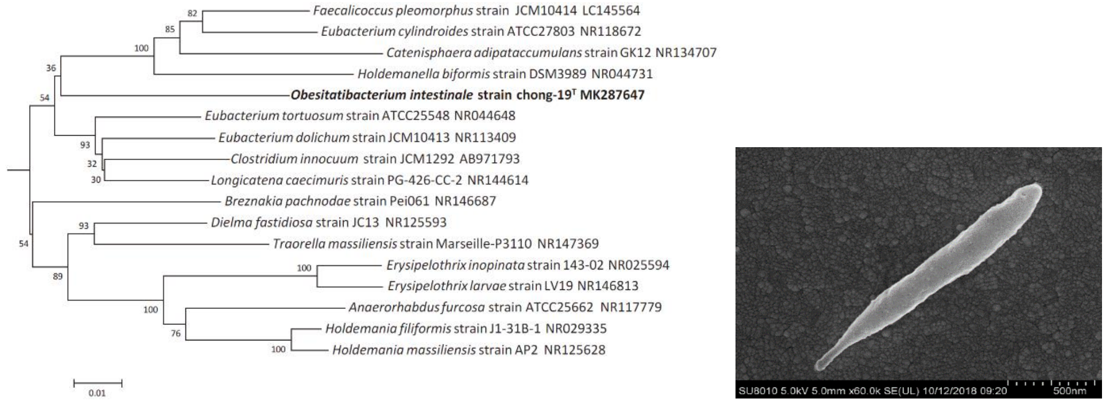


Supplementary Figure 72. The Neighbour-joining phylogenetic tree based on 16S rRNA gene sequences (left, a) and the TEM-based cell morphologic images (right, b) of strain chong-19$^{T}$. GenBank accession numbers are given in parentheses. Percentages of bootstrap support are shown at branch nodes. Bootstrap value was 1000. Bar: 0.01 substitutions per nucleotide position.

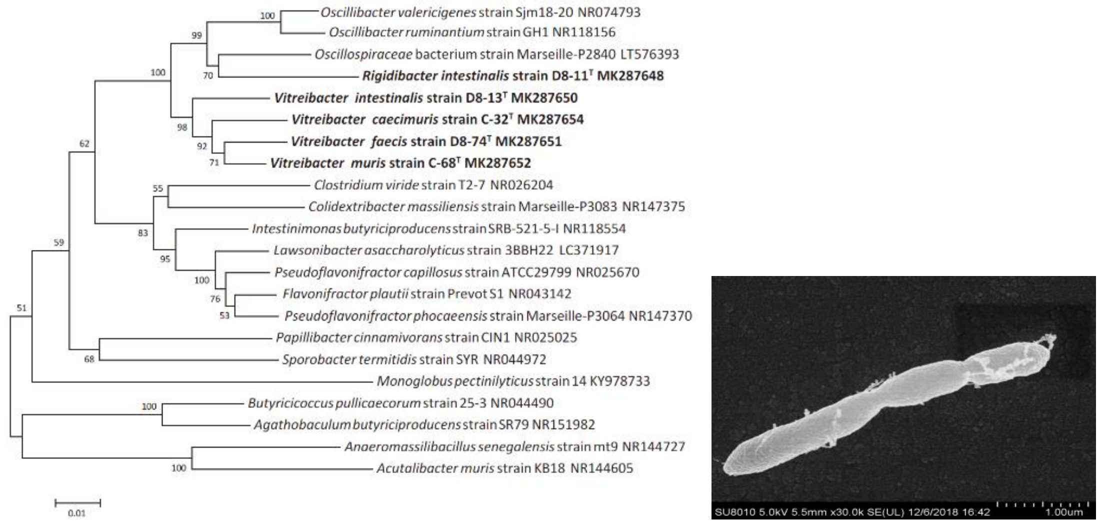


Supplementary Figure 73. The Neighbour-joining phylogenetic tree based on 16S rRNA gene sequences (left, a) and the TEM-based cell morphologic images (right, b) of strain D8-11$^{T}$. GenBank

accession numbers are given in parentheses. Percentages of bootstrap support are shown at branch nodes. Bootstrap value was 1000. Bar: 0.01 substitutions per nucleotide position.

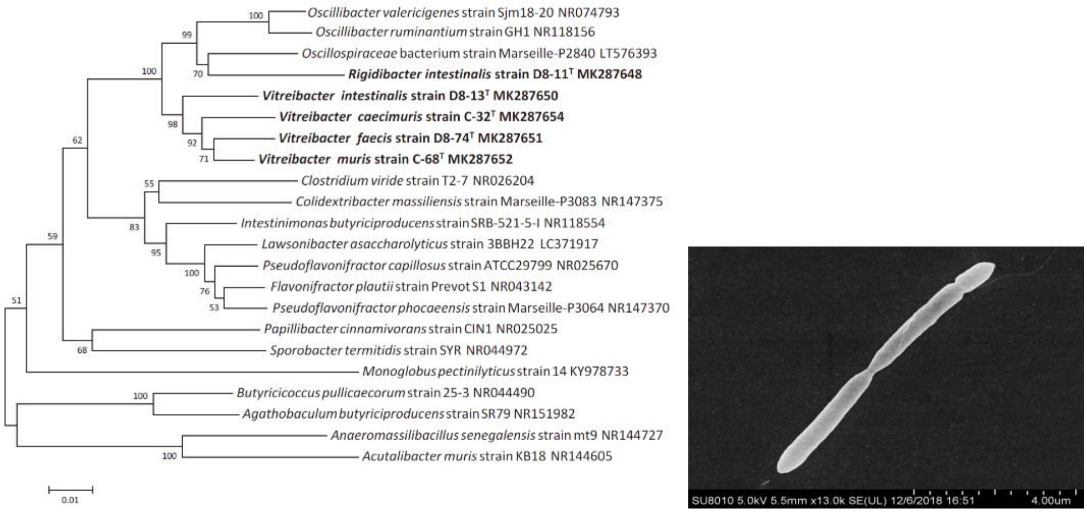


Supplementary Figure 74. The Neighbour-joining phylogenetic tree based on 16S rRNA gene sequences (left, a) and the TEM-based cell morphologic images (right, b) of strain D8-13$^{T}$. GenBank accession numbers are given in parentheses. Percentages of bootstrap support are shown at branch nodes. Bootstrap value was 1000. Bar: 0.01 substitutions per nucleotide position.

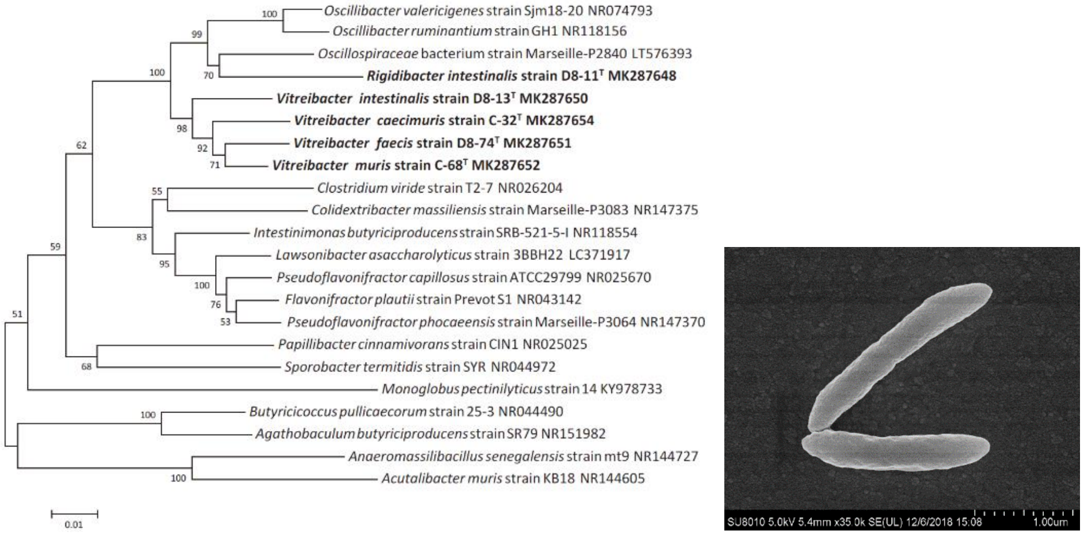


Supplementary Figure 75. The Neighbour-joining phylogenetic tree (left, a) based on 16S rRNA gene sequence and TEM-based cell morphologic images (right, b) of strain D8-74$^{T}$. GenBank accession numbers are given in parentheses. Percentages of bootstrap support are shown at branch nodes. Bootstrap value is 1000. Bar: 0.01 substitutions per nucleotide position.

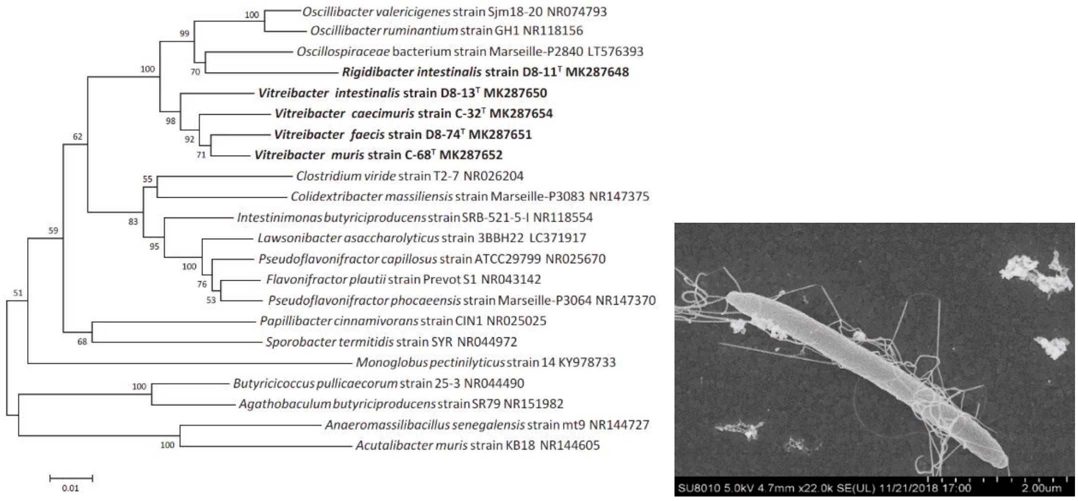


Supplementary Figure 76. The Neighbour-joining phylogenetic tree (left, a) based on 16S rRNA gene sequence and TEM-based cell morphologic images (right, b) of strain C-32$^T$. GenBank accession numbers are given in parentheses. Percentages of bootstrap support are shown at branch nodes. Bootstrap value is 1000. Bar: 0.01 substitutions per nucleotide position.

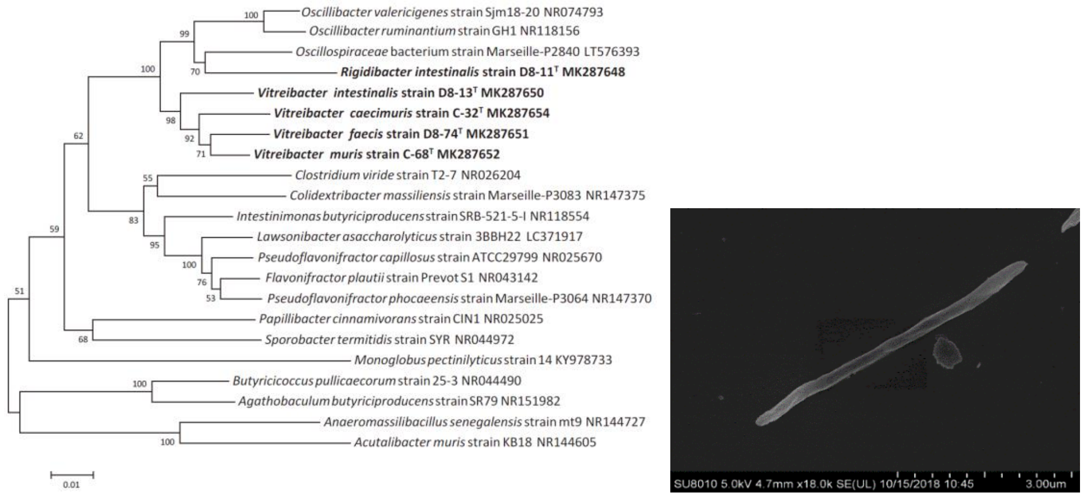


Supplementary Figure 77. The Neighbour-joining phylogenetic tree (left, a) based on 16S rRNA gene sequence and TEM-based cell morphologic images (right, b) of strain C-68$^T$. GenBank accession numbers are given in parentheses. Percentages of bootstrap support are shown at branch nodes. Bootstrap value is 1000. Bar: 0.01 substitutions per nucleotide position.

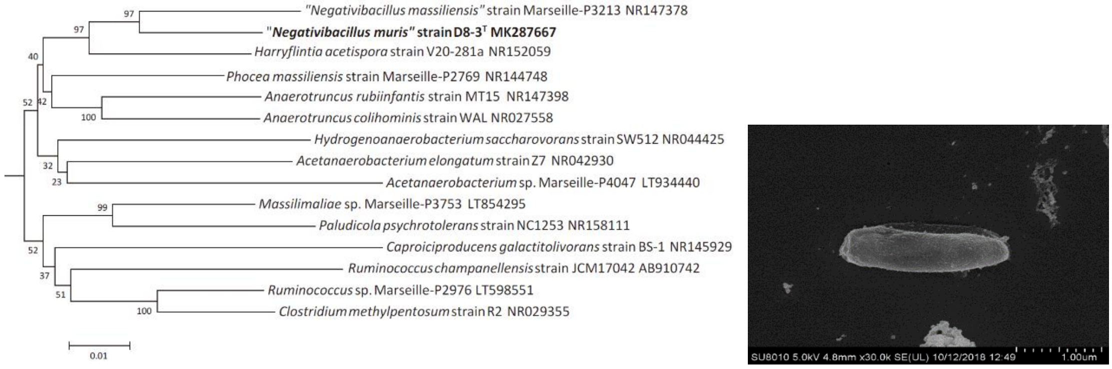


Supplementary Figure 78. The Neighbour-joining phylogenetic tree (left, a) based on 16S rRNA gene sequence and TEM-based cell morphologic images (right, b) of strain D8-3$^T$. GenBank

accession numbers are given in parentheses. Percentages of bootstrap support are shown at branch nodes. Bootstrap value is 1000. Bar: 0.01 substitutions per nucleotide position.

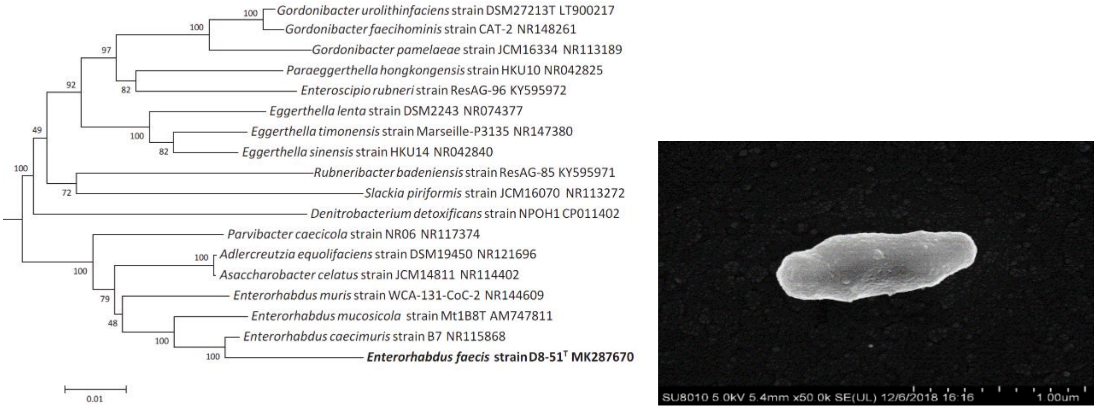


Supplementary Figure 79. The Neighbour-joining phylogenetic tree (left, a) based on 16S rRNA gene sequence and TEM-based cell morphologic images (right, b) of strain D8-51$^{T}$. GenBank accession numbers are given in parentheses. Percentages of bootstrap support are shown at branch nodes. Bootstrap value is 1000. Bar: 0.01 substitutions per nucleotide position.

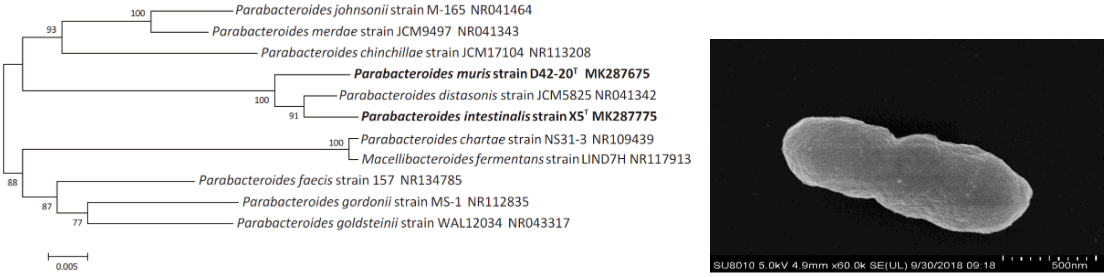


Supplementary Figure 80. The Neighbour-joining phylogenetic tree (left, a) based on 16S rRNA gene sequence and TEM-based cell morphologic images (right, b) of strain D42-20$^{T}$. GenBank accession numbers are given in parentheses. Percentages of bootstrap support are shown at branch nodes. Bootstrap value is 1000. Bar: 0.01 substitutions per nucleotide position.

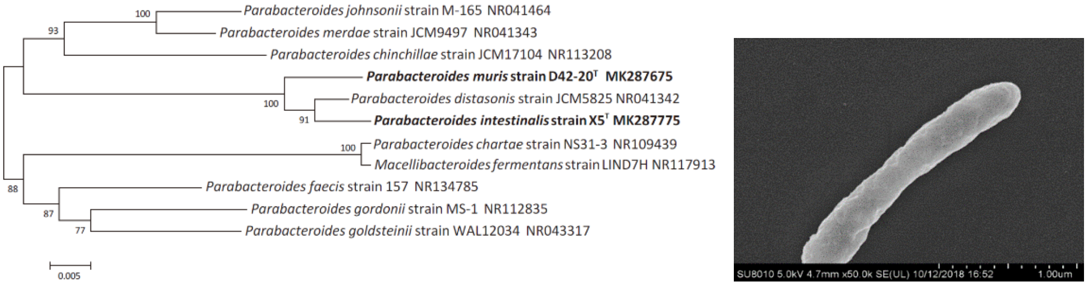


Supplementary Figure 81. The Neighbour-joining phylogenetic tree (left, a) based on 16S rRNA gene sequence and TEM-based cell morphologic images (right, b) of strain X5$^{T}$. GenBank accession numbers are given in parentheses. Percentages of bootstrap support are shown at branch nodes. Bootstrap value is 1000. Bar: 0.005 substitutions per nucleotide position.
