# Supplementary material for: The Mouse Gut Microbial Biobank expands the coverage of cultured bacteria
Source: Nat Commun. 2020 Jan 7;11:79. doi: 10.1038/s41467-019-13836-5 (PMC6946648; doi:10.1038/s41467-019-13836-5)
Supplement: Supplementary file 2 — Description of Additional Supplementary Files [file 41467_2019_13836_MOESM2_ESM.docx]

**Description of Additional Supplementary Files**

**File Name: Supplementary Data 1**

**Description:** The 16S rRNA sequences and identities of all isolates.

**File Name: Supplementary Data 2**

**Description:** Description of novel taxa in mGMB.

**File Name: Supplementary Data 3**

**Description:** The basic information of the strains in mGMB.

**File Name: Supplementary Data 4**

**Description:** The prevalence of 77 novel taxa in the host-associated microbiota from diverse hosts.

**File Name: Supplementary Data 5**

**Description:** The KO profiles of the metagenomes of ob/ob mice and C57BL/6, the gene catalog of mouse gut microbiota and the genomes in mGMB.
