# Supplementary material for: The Mouse Gut Microbial Biobank expands the coverage of cultured bacteria
Source: Nat Commun. 2020 Jan 7;11:79. doi: 10.1038/s41467-019-13836-5 (PMC6946648; doi:10.1038/s41467-019-13836-5)
Supplement: Supplementary file 8 — Reporting Summary [file 41467_2019_13836_MOESM8_ESM.pdf]

nature research

Corresponding author(s): *Double-blind peer review submissions: write DBPR and your manuscript number here instead of author names.*

Last updated by author(s): *YYYY-MM-DD*

Reporting Summary

Nature Research wishes to improve the reproducibility of the work that we publish. This form provides structure for consistency and transparency in reporting. For further information on Nature Research policies, see Authors & Referees and the Editorial Policy Checklist .

Statistics

For all statistical analyses, confirm that the following items are present in the figure legend, table legend, main text, or Methods section.

n/a	Confirmed	
[ ]	[x]	The exact sample size (*n*) for each experimental group/condition, given as a discrete number and unit of measurement
[x]	[ ]	A statement on whether measurements were taken from distinct samples or whether the same sample was measured repeatedly
[x]	[ ]	The statistical test(s) used AND whether they are one- or two-sided<br>*Only common tests should be described solely by name; describe more complex techniques in the Methods section.*
[x]	[ ]	A description of all covariates tested
[x]	[ ]	A description of any assumptions or corrections, such as tests of normality and adjustment for multiple comparisons
[ ]	[x]	A full description of the statistical parameters including central tendency (e.g. means) or other basic estimates (e.g. regression coefficient) AND variation (e.g. standard deviation) or associated estimates of uncertainty (e.g. confidence intervals)
[ ]	[x]	For null hypothesis testing, the test statistic (e.g. *F*, *t*, *r*) with confidence intervals, effect sizes, degrees of freedom and *P* value noted<br>*Give P values as exact values whenever suitable.*
[x]	[ ]	For Bayesian analysis, information on the choice of priors and Markov chain Monte Carlo settings
[x]	[ ]	For hierarchical and complex designs, identification of the appropriate level for tests and full reporting of outcomes
[x]	[ ]	Estimates of effect sizes (e.g. Cohen's *d*, Pearson's *r*), indicating how they were calculated

Our web collection on statistics for biologists contains articles on many of the points above.

Software and code

Policy information about availability of computer code

Data collection

Sratoolkit v2.8.0, NCBI (Jul, 2018), Ezbiocloud (28 Jun, 2019), KEGG (version 76), iMGMC (https://github.com/tillrobin/iMGMC)

Data analysis

BLAST+ v2.7.1, SOAPdenovo v2.04, glimmer v3, Genome-to-Genome Distance Calculator (GGDC) v2.1, JSpeciesWS v3.2.1, Anvi'o v5.0, Usearch v11, Cutadapt v1.18, Readfq v8, Bowtie v2.2.4, MEGAHIT v1.1.2, CD-HIT v4.5.8, SAMtools v0.1.19, IBM SPSS Statistics v20, RStudio (v1.1.383), R (v.3.4.4), Graph Pad Prism v6, MetaGeneMark, Prokka v1.13.3,

Customized code for generating the cumulative curve in R was as following:

```
library("vegan")
test <- read.table(file="rare.txt",header = T)
test1 <- test[,-1]
for (i in 1:10) { test3 <- test1[,sample(1:ncol(test1),1)];
ratio <- (nrow(test1)-sum(test3==0))/nrow(test1)*100;
write.table(ratio,file="temp.txt",sep=" ",append = T, col.names=F, row.names = F);
for ( j in 2:ncol(test1)) { test2 <- test1[,sample(1:ncol(test1),j)];
ratio <- (nrow(test1)-sum(rowSums(test2)==0))/nrow(test1)*100;
write.table(ratio,file="temp.txt",sep=" ",append = T, col.names=F, row.names=F) } }
allratio <- read.table(file="temp.all different KO.txt")
allratio <- as.matrix(allratio)
allratio <- matrix(allratio, nrow=10, byrow=T, dimnames=NULL)
write.table(allratio,file="statistic.txt",sep="\t",append = T, col.names=T, row.names=T)
```

For manuscripts utilizing custom algorithms or software that are central to the research but not yet described in published literature, software must be made available to editors/reviewers. We strongly encourage code deposition in a community repository (e.g. GitHub). See the Nature Research guidelines for submitting code & software for further information.

Data

Policy information about availability of data

All manuscripts must include a data availability statement. This statement should provide the following information, where applicable:

- Accession codes, unique identifiers, or web links for publicly available datasets
- A list of figures that have associated raw data
- A description of any restrictions on data availability

The datasets generated an analyzed in this study were available as following: The raw data of 16S rRNA gene amplicons was deposited in NCBI SRA (Accession: SRR8077557-80). All the genomic and metagenomic data obtained in this study are available at NODE with the project accession OEP000211 [https://www.biosino.org/node/project/detail/ OEP000211], NCBI under Project PRJNA486904 [https://www.ncbi.nlm.nih.gov/bioproject/PRJNA486904] and gcMeta under Project NMDC10010898 [https://gcmeta.wdcm.org/signin/?next=/%20NMDC10010898]. The GeneBank ID of the 16S rRNA gene sequences of all taxa in mGMB are MK287622-MK287775 and MN081616-MN081733. The other datasets analyzed in this study were available at NCBI with accessions of PRJNA486904 [https://www.ncbi.nlm.nih.gov/bioproject/ PRJNA486904], PRJNA400789 [https://www.ncbi.nlm.nih.gov/bioproject/PRJNA400789], PRJNA418420 [https://www.ncbi.nlm.nih.gov/bioproject/PRJNA418420], PRJNA417284 [https://www.ncbi.nlm.nih.gov/bioproject/PRJNA417284], PRJNA474117 [https://www.ncbi.nlm.nih.gov/bioproject/PRJNA474117], PRJEB11650 [https://www.ncbi.nlm.nih.gov/bioproject/PRJEB11650], PRJNA393083 [https://www.ncbi.nlm.nih.gov/ioproject/PRJNA393083], PRJNA388263 [https://www.ncbi.nlm.nih.gov/bioproject/PRJNA388263], PRJNA508548 [https://www.ncbi.nlm.nih.gov/bioproject/PRJNA508548], PRJNA453406 [https://www.ncbi.nlm.nih.gov/bioproject/PRJNA453406], PRJDB4202 [https://www.ncbi.nlm.nih.gov/bioproject/PRJDB4202] and at iMGMC ([https://github.com/tillrobin/iMGMC)]. The source data underlying Figures 1, 2, 3, 4 and Table 1 are provided as a Source Data file.

Field-specific reporting

Please select the one below that is the best fit for your research. If you are not sure, read the appropriate sections before making your selection.

- [x] Life sciences
- [ ] Behavioural & social sciences
- [ ] Ecological, evolutionary & environmental sciences

For a reference copy of the document with all sections, see nature.com/documents/nr-reporting-summary-flat.pdf

Life sciences study design

All studies must disclose on these points even when the disclosure is negative.

Sample size	This study collected 12 cecal content from ob/ob mice for large-scale bacterial isolation and cultivation. As a result, 77novel species were firstly characterized, and the largest-to-date mouse gut microbial collection containing 126 different species were constructed.
Data exclusions	No data were excluded from analyses.
Replication	not applicable
Randomization	not applicable
Blinding	not applicable

Reporting for specific materials, systems and methods

We require information from authors about some types of materials, experimental systems and methods used in many studies. Here, indicate whether each material, system or method listed is relevant to your study. If you are not sure if a list item applies to your research, read the appropriate section before selecting a response.

Materials & experimental systems

n/a	Involved in the study	
x		Antibodies
x		Eukaryotic cell lines
x		Palaeontology
	x	Animals and other organisms
x		Human research participants
x		Clinical data

Methods

n/a	Involved in the study	
x		ChIP-seq
x		Flow cytometry
x		MRI-based neuroimaging

Animals and other organisms

Policy information about studies involving animals; ARRIVE guidelines recommended for reporting animal research

Laboratory animals	Eight-week male wild-type C57BL/6J and ob/ob C57BL/6J mice.

Wild animals	Not involved
Field-collected samples	Not involved
Ethics oversight	All procedures were performed in accordance with the Guide for the Care and Use of Laboratory Animals and approved by the Institute of Microbiology, Chinese Academy of Sciences (IMCAS) Ethics Committee.

Note that full information on the approval of the study protocol must also be provided in the manuscript.
